# Comparing the performance of time series models with or without meteorological factors in predicting incident pulmonary tuberculosis in eastern China

**DOI:** 10.1186/s40249-020-00771-7

**Published:** 2020-11-05

**Authors:** Zhong-Qi Li, Hong-Qiu Pan, Qiao Liu, Huan Song, Jian-Ming Wang

**Affiliations:** 1 Department of Epidemiology, Center for Global Health, School of Public Health, Nanjing Medical University, 101 Longmian Ave., Nanjing, 211166 China; 2 Department of Tuberculosis, The Third Hospital of Zhenjiang City, Zhenjiang, 212005 China

**Keywords:** Pulmonary tuberculosis, Meteorological factor, Time series, Predicting

## Abstract

**Background:**

Many studies have compared the performance of time series models in predicting pulmonary tuberculosis (PTB), but few have considered the role of meteorological factors in their prediction models. This study aims to explore whether incorporating meteorological factors can improve the performance of time series models in predicting PTB.

**Methods:**

We collected the monthly reported number of PTB cases and records of six meteorological factors in three cities of China from 2005 to 2018. Based on this data, we constructed three time series models, including an autoregressive integrated moving average (ARIMA) model, the ARIMA with exogenous variables (ARIMAX) model, and a recurrent neural network (RNN) model. The ARIMAX and RNN models incorporated meteorological factors, while the ARIMA model did not. The mean absolute percentage error (MAPE) and root mean square error (RMSE) were used to evaluate the performance of the models in predicting PTB cases in 2018.

**Results:**

Both the cross-correlation analysis and Spearman rank correlation test showed that PTB cases reported in the study areas were related to meteorological factors. The predictive performance of both the ARIMA and RNN models was improved after incorporating meteorological factors. The MAPEs of the ARIMA, ARIMAX, and RNN models were 12.54%, 11.96%, and 12.36% in Xuzhou, 15.57%, 11.16%, and 14.09% in Nantong, and 9.70%, 9.66%, and 12.50% in Wuxi, respectively. The RMSEs of the three models were 36.194, 33.956, and 34.785 in Xuzhou, 34.073, 25.884, and 31.828 in Nantong, and 19.545, 19.026, and 26.019 in Wuxi, respectively.

**Conclusions:**

Our study revealed a possible link between PTB and meteorological factors. Taking meteorological factors into consideration increased the accuracy of time series models in predicting PTB, and the ARIMAX model was superior to the ARIMA and RNN models in study settings.

## Background

Tuberculosis (TB) is a chronic communicable disease that severely threatens human health, ranking among the top ten causes of death worldwide. The World Health Organization (WHO) estimated that approximately 10 million people fell ill with TB around the world in 2019. Furthermore, there were an estimated 1.2 million TB deaths among HIV-negative people and 208 000 TB deaths among HIV-positive people [1]. To curb the TB epidemic, the WHO set a goal of reducing the morbidity and mortality of TB by 90% and 95%, respectively, between 2015 and 2035. Accurately predicting the trend of this epidemic can help foresee the possible peaks and provide a reference for the prevention and control of TB [2].

A time series is formed by recording the development process of a random event over time. Time series analysis plays a vital role in predicting trends by identifying the way in which health-related events change with time. The autoregressive integrated moving average (ARIMA) model is the most classic time series analysis model and has been widely applied to predict various infectious diseases, such as hepatitis B [3], hemorrhagic fever with renal syndrome [4], coronavirus disease 2019 [5], and hand, foot and mouth disease [6]. The ARIMA with exogenous variables (ARIMAX) model exhibits superior prediction performance by adding other event-related factors as input variables. Another commonly used time series analysis model is based on an artificial neural network (ANN), which is designed to simulate the way the human brain analyzes and processes information. The ANN has been applied to construct time series models to forecast human diseases [7, 8]. The recurrent neural network (RNN) is a specific ANN with the ability to transfer information across time steps, as it can remember previous information and apply it to the current output calculation. The ability to model temporal dependencies makes it particularly appropriate to analyze a time series, which consists of a sequence of points that are not independent [9, 10].

Time series analyses have been used to predict TB morbidity or mortality, but most were conducted in one city or one region and based on one or two models that did not incorporate meteorological factors [11, 12]. Our previous study has revealed that the incidence of TB exhibits seasonal fluctuations, indicating a potential relationship with meteorological factors [13]. Thus, in the current study, we performed a time series analysis in three cities of Jiangsu Province, China, and applied different models (ARIMA, ARIMAX, and RNN) to explore whether the inclusion of meteorological factors can improve the performance of prediction modeling.

## Methods

### Study areas

**Fig. 1:**
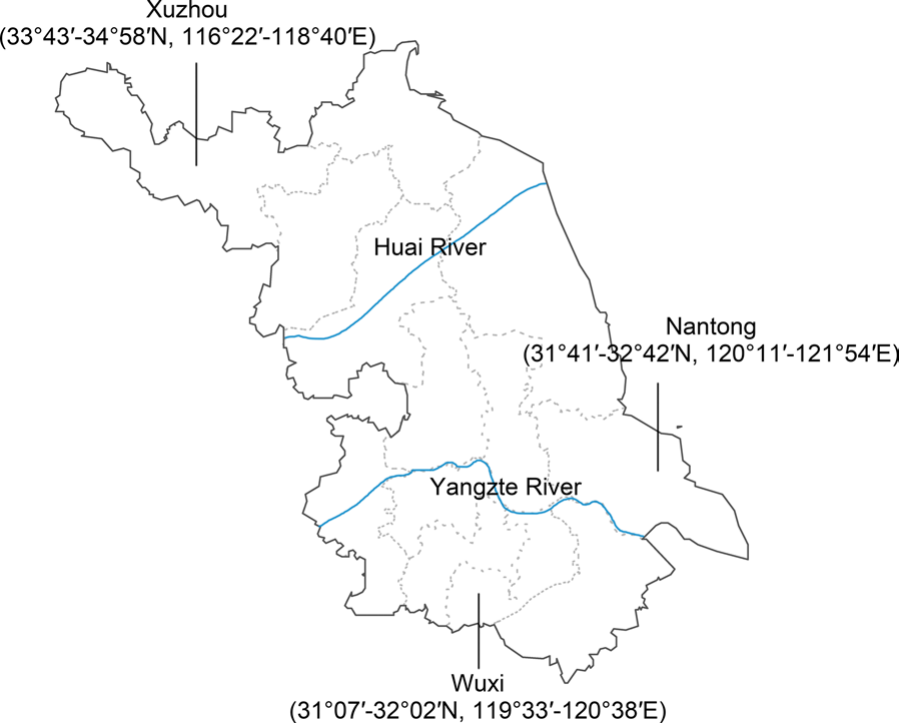
Geographical locations of the three cities in Jiangsu Province

Jiangsu Province is located on the eastern coast of China, with an area of 107 200 square kilometers. It governed 13 cities and had a permanent population of 80.7 million at the end of 2019. We randomly selected one city from northern, central, and southern Jiangsu and finally included Xuzhou, Nantong, and Wuxi as the study sites. The geographical locations of the three cities in Jiangsu Province are shown in Fig. 1. The ranking of the gross domestic product (GDP) per capita within the province in 2019 was 9 for Xuzhou, 7 for Nantong, and 1 for Wuxi, and the population density in 2019 was 750.16 people/m^2^ for Xuzhou, 914.64 people/m^2^ for Nantong and 1424.43 people/m^2^ for Wuxi.

### Data collection

All newly diagnosed PTB cases in China are registered in an online surveillance system (https://10.249.6.18:8880/) operated by the Center for Disease Control and Prevention. The registry system is a particular virtual private network. For confidentiality, only authorized organizations can log in. We extracted the monthly reported number of pulmonary TB (PTB) cases in the study sites between 2005 and 2018. We also collected local meteorological factors at the same time from the China Meteorological Data Network (https://www.nmic.cn/). These meteorological factors included monthly average temperature (MAT, °C), monthly average atmospheric pressure (MAP, hPa), monthly average wind speed (MAS, m/s), monthly average relative humidity (MAH, %), monthly precipitation (MP, mm), and monthly sunshine time (MST, h).

### Construction of the ARIMA model

As described in our previous study [13], we constructed a seasonal ARIMA model, which was expressed as ARIMA (p, d, q)(P, D, Q)_s_. The variables p, d, and q represent the autoregressive model order, the number of ordinary differences, and the moving-average model order, respectively. The variables P, D, and Q represent the seasonal autoregressive model order, the number of seasonal differences, and the seasonal moving-average model order, respectively. Variables represent the length of a periodic pattern (s = 12 in this study). The number of PTB cases predicted at time t (${Y}_{t}$) was determined by the formula:$Y_{t} = \frac{{{\uptheta }_{q} \left( B \right){\Theta }_{Q} \left( {B^{s} } \right)a_{t} }}{{{\Phi }_{P} \left( {B^{s} } \right)\phi_{p} \left( B \right)\left( {1 - B} \right)^{d} \left( {1 - B^{s} } \right)^{D} }}$, where ${\uptheta }_{q}(B)$ is the operator of the moving-average model, ${\Theta }_{Q}({B}^{s})$ is the operator of the seasonal-moving average model, ${\phi }_{p}(B)$ is the operator of the autoregressive model, ${\Phi }_{P}({B}^{s})$ is the operator of the seasonal autoregressive model, ${(1-B)}^{d}$ is the component of the ordinary differences, ${(1-{B}^{s})}^{D}$ is the component of the seasonal differences, ${a}_{t}$ is white noise and ${Y}_{t}$ is the predicted variable [14, 15]. Based on the monthly number of PTBs, we constructed an ARIMA model for each city. First, we applied the ordinary differences and seasonal differences to make the series stationary. Second, by referring to the autocorrelation function (ACF) and partial autocorrelation function (PACF) plots of the stationary series, we initially identified the values of the parameters (p, q, P, and Q) to establish alternative ARIMA models. Third, we determined the optimal ARIMA model according to three criteria: (a) the normalized value of Bayesian information criterion (BIC; smaller values indicated better models); (b) the degree to which the residual series of the model was demonstrated to be white noise by the Ljung-Box test; (c) the presence of significant parameters according to the parameter estimation. Finally, we selected the optimal ARIMA model to predict PTB cases in 2018.

### Construction of the ARIMAX model

The ARIMAX model adds exogenous variables based on the ARIMA model and can be described by the formula:${Y}_{t}=\frac{{\uptheta }_{q}(B){\Theta }_{Q}({B}^{s}){a}_{t}}{{\Phi }_{P}({B}^{s}){\phi }_{p}(B){(1-B)}^{d}{(1-{B}^{s})}^{D}}+X$, where $X$ represents the external regressor, which can be univariate or multivariate. The other parameters are consistent with the ARIMA model [14]. Based on the monthly number of PTB cases and six meteorological factors, we constructed an ARIMAX model for each city. First, we constructed the optimal ARIMA model for each meteorological factor and obtained the residual series of the optimal ARIMA models, ensuring that they were all white noise. Second, we used the cross-correlation function (CCF) to analyze the residual series of PTB cases and meteorological factors to evaluate the correlation between them at different lag times. Third, we included different combinations of significant meteorological factors as external variables into the optimal ARIMA model to construct alternative ARIMAX models. Finally, we determined the optimal ARIMAX model according to three criteria: (a) a normalized BIC value smaller than the optimal value; (b) the degree to which the residual series of the model was demonstrated to be white noise by the Ljung-Box test; (c) the performance of the model in predicting PTB cases in 2018.

### Construction of the RNN model

**Fig. 2:**
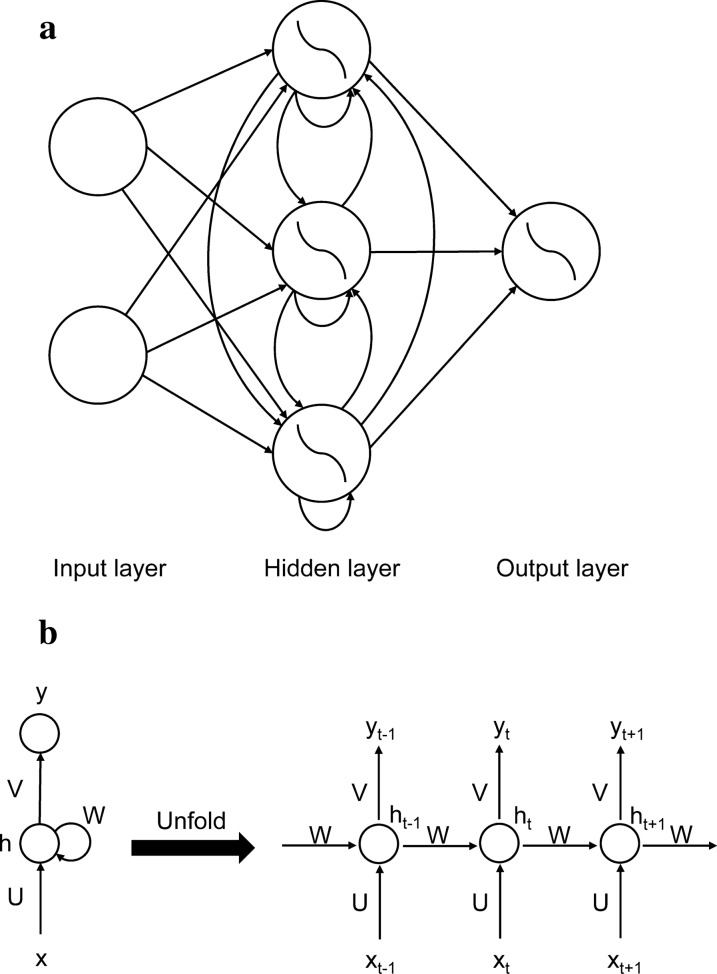
The recurrent neural network (RNN). **a** The structure diagram of the RNN; **b** The unfolding diagram of the forward propagation of the RNN. ${X}_{t}$: input at time $t$; ${h}_{t}$: hidden state at time $t$; ${y}_{t}$: output at time $t$; $W$: weight of the input; $U$: weight of the input at the moment; $V$: weight of the output

The ANN usually consists of an input layer, a hidden layer, and an output layer. The layers of the traditional ANN are fully connected, but the neurons in each layer are not connected. The RNN is different from the ANN in that it adds connections between the neurons in the hidden layer (Fig. 2a). Figure 2b shows the unfolding diagram of the forward propagation of the RNN [16], where ${x}_{t}$ represents the input at time $t$, ${h}_{t}$ represents the hidden state at time $t$ and is modeled as ${h}_{t}=sigmoid(W*{h}_{t-1}+U*{x}_{t})$, $W$ represents the weight of the input, $U$ represents the weight of the input at the moment, ${y}_{t}$ represents the output at time $t$, ${y}_{t}=softmax(V*{h}_{t})$, and $V$ represents the weight of the output. Therefore, the input of the hidden layer of the RNN includes not only the output of the input layer but also the previous output of the hidden layer, granting the model memory. We divided the data into a training set, testing set, and predicting set. We trained each RNN model three times and compared their performance on the testing set to determine the optimal RNN model. For each RNN model, we set the learning rate to 0.05, 0.1, and 0.2 and the dimensions of the hidden layer to 3, 5, and 10, respectively, and identified the appropriate training epochs through an epoch-error plot. By comparing the performance of the model with the testing set, we determined the most suitable parameters for each RNN model. First, we normalized the original data to convert all values to intervals [0, 1], using the formula: ${\mathrm{X}}^{\prime}=\frac{X-{X}_{min}}{{X}_{max}-{X}_{min}}$, where $\mathrm{X}$ is the original value, ${X}_{max}$ is the maximum value of the original data, ${X}_{min}$ is the minimum value of the original data, and ${\mathrm{X}}{^{\prime}}$ is the normalized value after conversion. Second, we used the number of PTB cases in the previous month and the previous two, three, six, and twelve months as sequential inputs of the training set and the number of PTB cases in the current month as the output of the training set to construct five different RNN models (RNN1–RNN5), which did not incorporate meteorological factors. We compared the performance of five RNN models on the testing set and selected the best model to incorporate meteorological factors into it. Third, we used the Spearman rank correlation test to evaluate the correlation between PTB cases in the current month and meteorological factors one, two, and three months prior. Fourth, we incorporated the significant meteorological factors into the best model of RNN1-RNN5 to construct another four RNN models (RNN6–RNN9). Finally, we compared the performance of nine RNN models on the testing set to determine the optimal model and applied it to predict PTB cases in 2018.

### Evaluating the performance of the three models

Considering that the mean absolute percentage error (MAPE) and root mean square error (RMSE) have been widely used to compare the performance of time series models [3, 17], they were used here to evaluate the performance of the three models: $\mathrm{MAPE}=\frac{1}{n}\sum_{i=1}^{n}\frac{\left|{X}_{i}-{\widehat{X}}_{i}\right|*100}{{X}_{i}}$ and $\mathrm{RMSE}=\sqrt{\frac{1}{n}\sum_{i=1}^{n} {({X}_{i}-{\widehat{X}}_{i})}^{2}}$, where ${X}_{i}$ is the actual value at time $i$, ${\widehat{X}}_{i}$ is the output value of the model at time $i$ and $n$ is the number of samples.

### Statistical software

We used SPSS 25.0 (IBM Corp., Armonk, NY, USA) to construct the ARIMA and ARIMAX models and the package “rnn” in R 3.6.3 (https://www.r-project.org/) to construct the RNN model. The significance level was set at 0.05.

## Results

### Description of the PTB notification rate and meteorological factors

The annual PTB notification rates between 2005 and 2017 of Xuzhou, Nantong, and Wuxi was 56.41/100 000, 59.93/100 000, and 57.10/100 000, respectively. The range of annual notification rates for PTB was 31.54/100 000 to 78.96/100 000 in Xuzhou, 35.42/100 000 to 92.63/100 000 in Nantong, and 43.65/100 000 to 87.13/100 000 in Wuxi. The description of the monthly meteorological factors in the three cities between 2005 and 2017 is listed in Additional file 1: Table S1.

### The ARIMA model

**Fig. 3:**
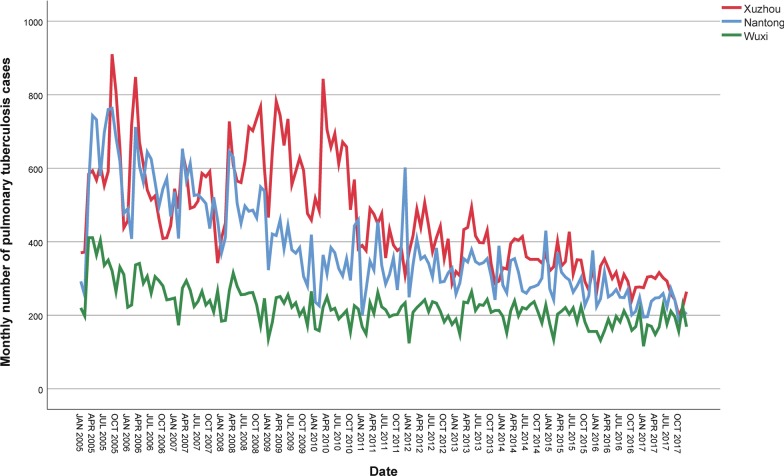
Monthly pulmonary tuberculosis cases in the three cities between 2005 and 2017

The monthly number of PTB cases showed a long-term downward trend and seasonal fluctuations, with a peak in March to April and a trough in December to January (in Xuzhou) or January to February (in Nantong and Wuxi) (Fig. 3). Therefore, we applied one ordinary difference and one seasonal difference to make the series stationary (d = D = 1). Then, we initially identified the parameters of the ARIMA model (p, q, P, and Q) to construct alternative models for each city according to the ACF and PACF plots of the stationary series (Additional file 1: Figure S1, a1–a3, and b1–b3). We determined the optimal ARIMA model to be ARIMA (1,1,1)(0,1,1)_12_ for Xuzhou and ARIMA (0,1,1)(0,1,1)_12_ for Nantong and Wuxi because (1) they had the smallest normalized BIC, (2) their residual series were demonstrated to be white noise, and (3) the parameters were all significant (*P* < 0.05) (Additional file 1: Table S2, c1–c3, and d1–d3 of Additional file 1: Figure S1). PTB cases in 2018 were predicted by the optimal ARIMA model and are listed in Table 1.

**Table 1:** The monthly number of pulmonary tuberculosis cases in the three cities in 2018 predicted by the ARIMA, ARIMAX, and RNN models

Month	Xuzhou city				Nantong city				Wuxi city			
	Observation	ARIMA	ARIMAX	RNN	Observation	ARIMA	ARIMAX	RNN	Observation	ARIMA	ARIMAX	RNN
January	237	239	227	258	193	145	177	194	158	137	135	141
February	188	259	249	249	177	149	163	202	112	143	141	170
March	300	323	304	227	261	216	258	209	194	174	175	177
April	273	291	277	285	221	195	202	219	188	177	184	139
May	271	288	283	282	251	196	212	206	206	178	181	194
June	273	293	286	285	230	196	204	224	183	202	204	188
July	229	242	237	250	204	167	186	200	218	183	187	193
August	248	257	237	242	166	195	205	218	206	198	200	214
September	169	255	247	215	170	189	170	207	203	195	199	192
October	208	206	238	192	151	134	125	178	150	160	170	177
November	193	209	243	180	149	146	150	183	191	183	181	199
December	241	206	240	201	162	195	212	190	177	178	181	179

*ARIMA* autoregressive integrated moving average, *ARIMAX* autoregressive integrated moving average with exogenous variables, *RNN* recurrent neural network

### The ARIMAX model

**Fig. 4:**
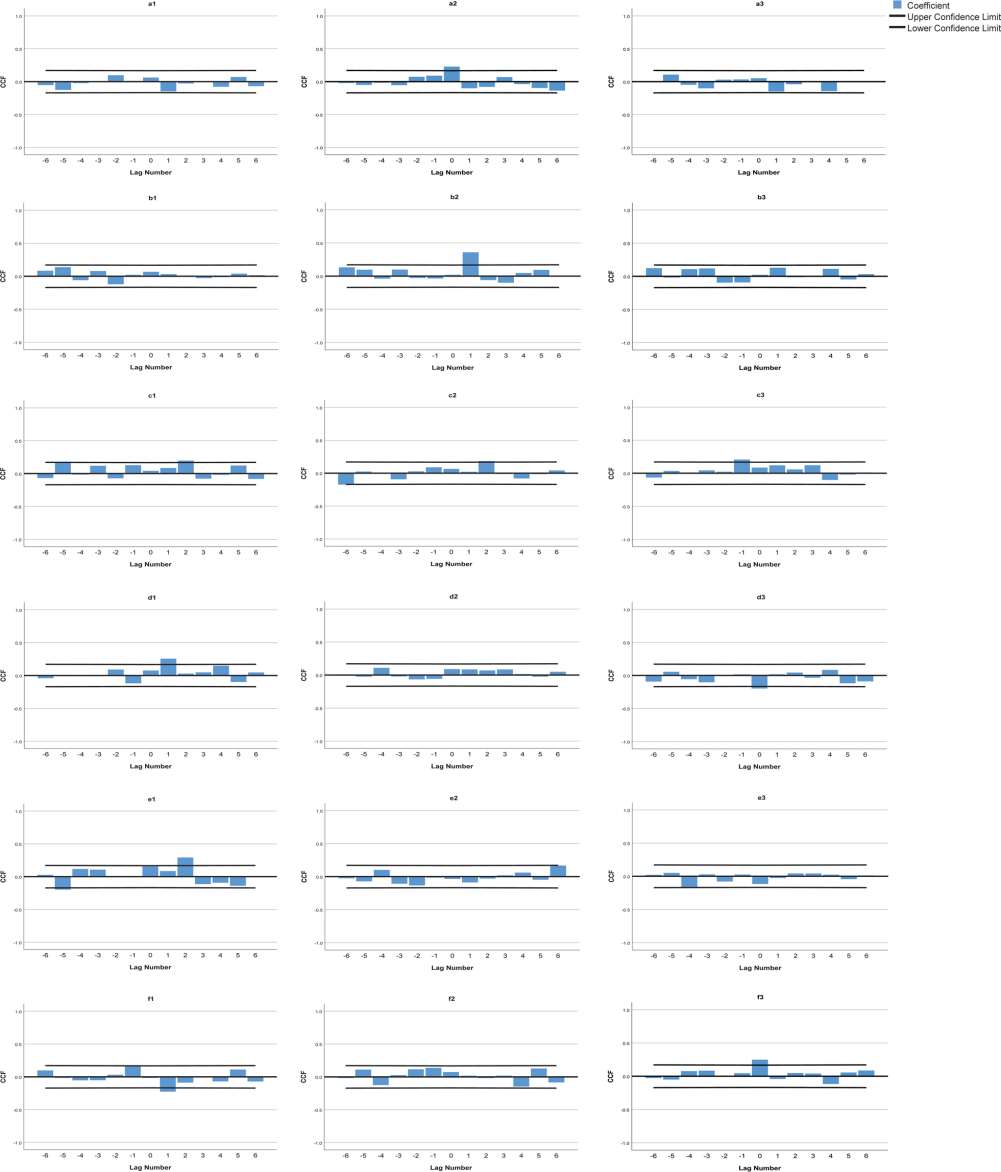
Cross-correlation function plots of the residual series of pulmonary tuberculosis and meteorological factors. **a**: PTB and MAT; **b** PTB and MAP; **c** PTB and MAS; **d** PTB and MAH; **e** PTB and MP; **f** PTB and MST; 1: Xuzhou; 2: Nantong; 3: Wuxi. PTB: Pulmonary tuberculosis; MAT: Monthly average temperature; MAP: Monthly average atmospheric pressure; MAS: Monthly average wind speed; MAH: Monthly average relative humidity; MP: Monthly precipitation; MST: Monthly sunshine time

The time series plots of the six meteorological factors in the three cities between 2005 and 2017 are shown in Additional file 1: Figure S2. The optimal ARIMA models for the MAT, MAP, MAS, MAH, MP and MST were ARIMA (0,0,0)(0,1,1)_12_, ARIMA (0,0,0)(0,1,1)_12_, ARIMA (0,1,1)(0,1,1)_12_, ARIMA (1,0,0)(2,1,0)_12_, ARIMA (0,0,0)(0,1,1)_12_, and ARIMA (0,1,1)(0,1,1)_12_ for Xuzhou, ARIMA (1,0,1)(0,1,1)_12_, ARIMA (0,0,1)(0,1,1)_12_, ARIMA (0,1,1)(1,1,0)_12_, ARIMA (1,1,1)(1,1,0)_12_, ARIMA (0,0,0)(0,1,1)_12_, and ARIMA (1,0,1)(0,1,1)_12_ for Nantong, and ARIMA (0,0,0)(2,1,0)_12_, ARIMA (0,0,1)(0,1,1)_12_, ARIMA (0,1,1)(0,1,1)_12_, ARIMA (0,1,2)(0,1,1)_12_, ARIMA (0,0,0)(0,1,1)_12_, and ARIMA (1,1,1)(0,1,1)_12_ for Wuxi, respectively. We then estimated the correlation between PTB and each meteorological factor at different lag times. The CCF plots showed that PTB was positively correlated with MAS (2-month lag), MAH (1-month lag) and MP (2-month lag) and negatively correlated with MST (1-month lag) in Xuzhou. PTB was positively correlated with MAT (0-month lag), MAP (1-month lag) and MAS (2-month lag) in Nantong and was positively correlated with MST (0-month lag) and negatively correlated with MAH (0-month lag) (*P* < 0.05) in Wuxi (Fig. 4). We incorporated different combinations of significant meteorological factors as external variables into the optimal ARIMA model to construct alternative ARIMAX models (Table 2). Finally, we determined the optimal ARIMAX model to be ARIMA (1,1,1)(0,1,1)_12_ with MP (2-month lag) for Xuzhou, ARIMA (0,1,1)(0,1,1)_12_ with MAP (1-month lag) for Nantong and ARIMA (0,1,1)(0,1,1)_12_ with MAH (0-month lag) for Wuxi. PTB cases in 2018 were predicted by the optimal ARIMAX model and are listed in Table 1.

**Table 2:** Alternative ARIMAX models for the three cities

City	Model	Normalized BIC value	P*	MAPE (%)^a^
Xuzhou	ARIMA (1,1,1)(0,1,1)_12_	8.857	0.861	12.54
	ARIMA (1,1,1)(0,1,1)_12_ + MAS2	8.595	0.714	14.05
	ARIMA (1,1,1)(0,1,1)_12_ + MAH1	8.467	0.399	24.09
	ARIMA (1,1,1)(0,1,1)_12_ + MP2	8.617	0.356	11.96
	ARIMA (1,1,1)(0,1,1)_12_ + MST1	8.593	0.767	17.62
	ARIMA (1,1,1)(0,1,1)_12_ + MAS2 + MAH1	8.609	0.338	25.02
	ARIMA (1,1,1)(0,1,1)_12_ + MAS2 + MP2	8.658	0.691	17.22
	ARIMA (1,1,1)(0,1,1)_12_ + MAS2 + MST1	8.679	0.902	17.34
	ARIMA (1,1,1)(0,1,1)_12_ + MAH1 + MP2	8.560	0.431	20.68
	ARIMA (1,1,1)(0,1,1)_12_ + MAH1 + MST1	8.604	0.416	24.30
	ARIMA (1,1,1)(0,1,1)_12_ + MP2 + MST1	8.674	0.751	17.55
	ARIMA (1,1,1)(0,1,1)_12_ + MAS2 + MAH1 + MP2	8.700	0.371	20.71
	ARIMA (1,1,1)(0,1,1)_12_ + MAS2 + MAH1 + MST1	8.755	0.427	23.01
	ARIMA (1,1,1)(0,1,1)_12_ + MAS2 + MP2 + MST1	8.755	0.851	17.21
	ARIMA (1,1,1)(0,1,1)_12_ + MAH1 + MP2 + MST1	8.692	0.241	39.17
	ARIMA (1,1,1)(0,1,1)_12_ + MAS2 + MAH1 + MP2 + MST1	8.831	0.581	17.44
Nantong	ARIMA (0,1,1)(0,1,1)_12_	8.609	0.433	15.57
	ARIMA (0,1,1)(0,1,1)_12_ + MAT0	8.288	0.981	16.77
	ARIMA (0,1,1)(0,1,1)_12_ + MAP1	8.183	0.777	11.16
	ARIMA (0,1,1)(0,1,1)_12_ + MAS2	8.323	0.730	16.29
	ARIMA (0,1,1)(0,1,1)_12_ + MAT0 + MAP1	8.340	0.836	14.99
	ARIMA (0,1,1)(0,1,1)_12_ + MAT0 + MAS2	8.419	0.965	16.97
	ARIMA (0,1,1)(0,1,1)_12_ + MAP1 + MAS2	8.314	0.766	11.90
	ARIMA (0,1,1)(0,1,1)_12_ + MAT0 + MAP1 + MAS2	8.470	0.892	13.06
Wuxi	ARIMA (0,1,1)(0,1,1)_12_	6.933	0.176	9.70
	ARIMA (0,1,1)(0,1,1)_12_ + MAH0	6.845	0.119	9.66
	ARIMA (0,1,1)(0,1,1)_12_ + MST0	6.818	0.068	10.51
	ARIMA (0,1,1)(0,1,1)_12_ + MAH0 + MST0	7.003	0.088	9.74

*BIC* Bayesian information criterion, *MAPE* mean absolute percentage error, *MAT* monthly average temperature; *MAP* monthly average atmospheric pressure, *MAS* monthly average wind speed, *MAH* monthly average relative humidity, *MP* Monthly precipitation, *MST* monthly sunshine time, *0* 0-month lag, *1* 1-month lag, *2* 2-month lag

^*^Ljung-Box test

^a^ MAPE of the model in predicting the monthly number of PTB cases in 2018

### The RNN model

We compared the MAPE of each RNN model with different parameters using the testing set to identify the appropriate parameters. The RNN5 model had the smallest MAPE with the testing set in each city (Table 3). The number of PTB cases in the current month in Xuzhou was positively correlated with MAS one month prior (*P* < 0.01), with MAS two months prior (*P* < 0.01), and with MAS three months prior (*P* < 0.01) and negatively correlated with MST two months prior (*P* < 0.01), with MAT three months prior (*P* < 0.01), with MP three months prior (*P* < 0.05), and with MST three months prior (*P* < 0.01). The number of PTB cases in the current month in Nantong was negatively correlated with MAS one month prior (*P* < 0.05), MAH one month prior (*P* < 0.05), MAS two months prior (*P* < 0.01), MAH two months prior (*P* < 0.01), MAS three months prior (*P* < 0.01), and MAH three months prior (*P* < 0.05). The number of PTB cases in the current month in Wuxi was positively correlated with MAT one month prior (*P* < 0.01), MAS one month prior (*P* < 0.01), MST one month prior (*P* < 0.05), MAS two months prior (*P* < 0.01), and MAS three months prior (*P* < 0.01) and negatively correlated with MAP one month prior (*P* < 0.01), MAH one month prior (*P* < 0.05), MAT three months prior (*P* < 0.05), and MAH three months prior (*P* < 0.05) (Additional file 1: Table S3). Then, we constructed the RNN6-RNN9 models by incorporating significant meteorological factors into the RNN5 model. The detailed composition of the nine RNN models is listed in Additional file 1: Table S4. We determined the optimal RNN model to be RNN8 for Xuzhou and RNN7 for Nantong and Wuxi since they had the smallest MAPE with the testing set after three training cycles (Table 3). Additional file 1: Figure S3 shows the epoch-error plots of the optimal RNN models after three training cycles. The downward trend in the error of the models with the training set was no longer significant after reaching the set number of epochs, indicating that the training epochs were appropriate. Finally, we chose the RNN8 model after the first training in Xuzhou and the RNN7 model after the second training in Nantong and Wuxi (Table 3). PTB cases in 2018 were predicted by the optimal RNN model and are listed in Table 1.

**Table 3:** Alternative recurrent neural network models for the three cities

City	Model	Learning rate	Dimensions of hidden layer	Number of epochs	MAPE (%)^a^	MAPE (%)^b^	MAPE (%)^c^
Xuzhou	RNN1	0.05	3	500	16.14	15.99	16.46
	RNN2	0.05	3	500	13.42	13.30	14.41
	RNN3	0.2	3	150	13.08	11.95	12.07
	RNN4	0.05	3	600	10.33	10.33	10.40
	RNN5	0.05	5	600	8.45	8.25	8.54
	RNN6 (RNN5 + MAS1)	0.05	3	1000	7.36	7.33	7.33
	RNN7 (RNN5 + MAS2 + MST2)	0.05	3	800	6.38	6.31	6.42
	RNN8 (RNN5 + MAT3 + MAS3 + MP3 + MST3)	0.05	5	600	4.78	4.89	4.97
	RNN9 (RNN5 + MAS1 + MAS2 + MST2 + MAT3 + MAS3 + MP3 + MST3)	0.05	10	600	5.75	5.40	5.90
Nantong	RNN1	0.05	3	500	21.91	21.99	21.78
	RNN2	0.2	5	80	16.92	17.81	16.31
	RNN3	0.2	3	150	13.82	14.26	13.86
	RNN4	0.2	3	150	12.78	12.84	12.80
	RNN5	0.2	5	100	11.38	11.44	11.24
	RNN6 (RNN5 + MAS1 + MAH1)	0.05	5	1000	9.19	8.82	8.84
	RNN7 (RNN5 + MAS2 + MAH2)	0.05	5	1000	8.58	8.26	8.52
	RNN8 (RNN5 + MAS3 + MAH3)	0.05	10	800	8.87	8.79	8.69
	RNN9 (RNN5 + MAS1 + MAH1 + MAS2 + MAH2 + MAS3 + MAH3)	0.05	5	800	8.79	9.21	9.19
Wuxi	RNN1	0.1	10	150	23.76	23.81	23.77
	RNN2	0.05	5	400	19.93	19.54	20.17
	RNN3	0.05	10	250	18.23	17.84	18.59
	RNN4	0.05	10	400	17.15	17.40	17.31
	RNN5	0.05	5	600	14.10	13.93	13.95
	RNN6 (RNN5 + MAT1 + MAP1 + MAS1 + MAH1 + MST1)	0.05	3	1500	13.01	13.39	13.04
	RNN7 (RNN5 + MAS2)	0.1	5	800	12.62	12.36	12.80
	RNN8 (RNN5 + MAT3 + MAS3 + MAH3)	0.05	10	1000	12.71	13.06	12.94
	RNN9 (RNN5 + MAT1 + MAP1 + MAS1 + MAH1 + MST1 + MAS2 + MAT3 + MAS3 + MAH3)	0.1	3	1000	12.81	12.80	13.46

*RNN* recurrent neural network, *MAPE* mean absolute percentage error, *MAT* monthly average temperature, *MAP* monthly average atmospheric pressure, *MAS* monthly average wind speed, *MAH* monthly average relative humidity, *MP* monthly precipitation, *MST* monthly sunshine time, *1* 1 month prior, *2* 2 months prior, *3* 3 months prior

^a^ MAPE of the model with the testing set after the first training

^b^ MAPE of the model with the testing set after the second training

^c^ MAPE of the model with the testing set after the third training

### Evaluating the performance of three models

As shown in Table 4, the ARIMAX model is slightly superior to the ARIMA and RNN models in Xuzhou, significantly superior to the ARIMA and RNN models in Nantong, and slightly superior to the ARIMA and significantly superior to the RNN models in Wuxi. Generally, the ARIMAX model showed the best performance.

**Table 4:** Evaluation of the performance of the ARIMA, ARIMAX, and RNN models in predicting the monthly number of pulmonary tuberculosis cases in the three cities in 2018

City	Diagnostic indicator	Model		
		ARIMA	ARIMAX	RNN
Xuzhou	MAPE (%)	12.54	11.96	12.36
	RMSE	36.194	33.956	34.785
Nantong	MAPE (%)	15.57	11.16	14.09
	RMSE	34.073	25.884	31.828
Wuxi	MAPE (%)	9.70	9.66	12.50
	RMSE	19.545	19.026	26.019

*ARIMA* autoregressive integrated moving average, *ARIMAX* autoregressive integrated moving average with exogenous variables, *RNN* recurrent neural network, *MAPE* mean absolute percentage error, *RMSE* root mean square error

## Discussion

In this study, we explored the role of meteorological factors in predicting PTB in three cities of China by constructing ARIMA, ARIMAX, and RNN models. The prediction ability of the models was improved by adding meteorological factors. The ARIMAX model (ARIMA with meteorological factors) showed the best performance. To our knowledge, this is the first time series study to construct different models in different cities to explore the role of meteorological factors in predicting PTB.

Although the notification rate of TB has declined at an annual rate of 3% between 2005 and 2017 [11], approximately 866 000 new cases were identified in China in 2018, second only to India [1]. Accurately forecasting the future trend of the TB epidemic can help policymakers implement effective interventions and distribute healthcare resources appropriately. Previous studies have explored various models, such as ARIMA [11, 18], X12-ARIMA [18], and ARIMA-generalized regression neural network (GRNN), in predicting TB [11]. However, few models have considered seasonal variation characteristics, socioeconomic levels, and meteorological factors [12, 19, 20]. Therefore, we divided the study areas into three regions according to geographical location and economic level and then compared the performance of different models with or without adding meteorological factors in predicting PTB in the Chinese population.

The ARIMA model, also known as the Box-Jenkins model, can analyze various types of time series data and is a commonly used model in time series analysis [3–6]. Unlike the ARIMA model, which is a univariate time series model, the ARIMAX model can deal with multivariate time series data. It adds other variables related to the target series as input variables to improve the prediction accuracy. A time series study in Guangzhou, China, showed that an ARIMA model with imported cases and minimum temperature as input variables was superior to a single ARIMA model in forecasting dengue transmission [14]. Another time series study in Abidjan, Cote d’Ivoire, also indicated that including rainfall as an input variable can increase the accuracy of the ARIMA model in predicting influenza [21]. However, when we incorporated two or more meteorological factors into the ARIMA model, its prediction performance did not continuously increase, which may be attributed to the high collinearity between the meteorological factors.

Considering that both ARIMA and ARIMAX are linear regression models, we also applied the RNN model, which has a strong nonlinear fitting ability. It can recognize the relationship between variables without any restrictions and has memory. This means that the RNN model uses as input not only current data but also its long-term experience. When constructing an RNN model, some parameters need to be determined artificially. In addition, since the initial weights and thresholds are random when training the RNN model, even for the same training set, the output of the model with the testing set will not be precisely the same. Therefore, we trained each RNN model with different parameters and compositions three times and compared their performance when using the testing set to determine the optimal RNN model. Finally, we found that the prediction performance of the RNN model was improved after incorporating meteorological factors.

The possible link between PTB and meteorological factors may be attributable to the following reasons. First, the temperature can affect the indoor and outdoor activities of TB patients and other susceptible people. For example, during hot summers and cold winters, people tend to stay indoors, which will increase the probability of *Mycobacterium tuberculosis* transmission [22]. Second, high wind speeds can dilute the concentration of environmental *M. tuberculosis*, thereby reducing the risk of infection. Airflow usually occurs from high-pressure areas to low-pressure areas, so the correlation between PTB and atmospheric pressure may be related to wind speed, but further exploration is needed [23]. Third, high relative humidity and abundant precipitation can provide an appropriate living environment for *M. tuberculosis *[23, 24]. Continuous exposure to dry air may decrease the production of protective mucus on the respiratory tract surface, thereby weakening its resistance to the pathogen [25]. Fourth, the large amount of ultraviolet light provided by long-term sunshine not only restricts the growth of *M. tuberculosis* but also promotes the synthesis of vitamin D, which can protect people from TB to some extent [23].

The association between PTB and meteorological factors varied across regions [23], which may be partially attributed to socioeconomic differences or analytic methods. TB is a poverty-related infectious disease [1]. Differences in economic level may lead to an uneven distribution of socioeconomic factors that affect the risk of TB, such as food and nutrition security, living condition, community environment, and medical resources [20, 26]. The inconsistency between analytical methods may be due to their different requirements for the data. The Spearman rank correlation test has no special requirements for the distribution of variables and has a wide range of applications. However, if there is a long-term trend in both time series, the Spearman test will yield a biased correlation. The cross-correlation analysis can evaluate the correlation between time series at different lag times without the influence of long-term trends. In addition, the exposure–response relationship between TB and meteorological factors might be nonlinear. For example, as mentioned earlier, TB may benefit from extremely high or extremely low temperatures and relative humidity. Both the Spearman rank correlation test and the cross-correlation analysis can perform linear correlation analyses between time series but have limitations in quantifying nonlinear relationships. Moreover, considering that most PTB cases are transmitted in dense indoor places, the effects of outdoor meteorological factors may be limited, resulting in inconsistency.

Our study has several limitations. First, the ARIMA, ARIMAX, and RNN models are all short-term prediction models; continuous data collection to update the models is essential for maintaining their prediction performance. Second, we incorporated all combinations of significant meteorological factors into the ARIMA model to construct the ARIMAX model, but we only incorporated four combinations of meteorological factors into the RNN model. In addition, the construction of the RNN model was based on monthly data, which may be insufficient for the RNN to reflect its predictive value. As the performance of the RNN model in this study was inferior to that of the ARIMAX model, its prediction performance needs further exploration. Third, we qualitatively evaluated only the linear correlation between PTB and meteorological factors based on monthly data. Considering that this relationship may be nonlinear and possess the lag time, we intend to apply the distributed lag nonlinear model to quantitatively evaluate it based on weekly or daily data in future studies. Fourth, most PTB cases are typically transmitted in dense indoor places, while all meteorological data in this study were derived from outdoor measurements, and indoor microclimates were not considered.

## Conclusions

The prediction performance of both the ARIMA and RNN models was improved after incorporating meteorological factors, and the ARIMAX model (ARIMA with meteorological factors) had the best performance, indicating a potential link between PTB and meteorological factors. Taking meteorological factors into consideration may increase the accuracy of time series models in predicting the trend of PTB.

## Supplementary information

**Additional file 1: Table S1.** Description of monthly meteorological factors in the three cities between 2005 and 2017. **Table S2.** Alternative ARIMA models for the three cities. **Table S3.** The Spearman rank correlation coefficients between the monthly number of PTB cases and meteorological factors in the three cities. **Table S4.** The detailed composition of the nine RNN models. **Figure S1.** ACF and PACF plots. **Figure S2.** Time series plots of the six meteorological factors in the three cities between 2005 and 2017. **Figure S3.** Epoch-error plots of the optimal RNN models of the three cities after three training cycles. **File S1.** R code.

## Data Availability

Please contact the author for the original data. The R codes can be found in Additional file 1: File S1.

## Abbreviations

TB: Tuberculosis
PTB: Pulmonary tuberculosis
ARIMA: Autoregressive integrated moving average
ARIMAX: Autoregressive integrated moving average with exogenous variables
ANN: Artificial neural network
RNN: Recurrent neural network
MAPE: Mean absolute percentage error
RMSE: Root mean square error
MAT: Monthly average temperature
MAP: Monthly average atmospheric pressure
MAS: Monthly average wind speed
MAH: Monthly average relative humidity
MP: Monthly precipitation
MST: Monthly sunshine time
ACF: Autocorrelation function
PACF: Partial autocorrelation function
BIC: Bayesian information criterion
CCF: Cross-correlation function
