# Supplementary material for: Comparing the performance of time series models with or without meteorological factors in predicting incident pulmonary tuberculosis in eastern China
Source: Infect Dis Poverty. 2020 Nov 5;9:151. doi: 10.1186/s40249-020-00771-7 (PMC7641658; doi:10.1186/s40249-020-00771-7)
Supplement: Supplementary file 1 — Additional file 1: Table S1. Description of monthly meteorological factors in the three cities between 2005 and 2017. Table S2. Alternative ARIMA models for the three cities. Table S3. The Spearman rank correlation coefficients between the monthly number of PTB cases and meteorological factors in the three cities. Table S4. The detailed composition of the nine RNN models. Figure S1. ACF and PACF plots. Figure S2. Time series plots of the six meteorological factors in the three cities between 2005 and 2017. Figure S3. Epoch-error plots of the optimal RNN models of the three cities after three training cycles. File S1. R code. [file 40249_2020_771_MOESM1_ESM.doc]

**Additional files**

**Additional Table S1. Description of monthly meteorological factors in the three cities between 2005 and 2017.**

**Additional Table S2. Alternative ARIMA models for the three cities.**

**Additional Table S3. The spearman rank correlation coefficients between the monthly number of PTB cases and meteorological factors in the three cities.**

**Additional Table S4. The detailed composition of the nine RNN models.**

**Additional Figure S1. ACF and PACF plots.**

a: ACF plots of the monthly number of PTB cases after one ordinary difference and one seasonal difference in the three cities; b: PACF plots of the monthly number of PTB cases after one ordinary difference and one seasonal difference in the three cities; c: ACF plots of the residual series of the optimal ARIMA models of the three cities; d: PACF plots of the residual series of the optimal ARIMA models of the three cities; 1: Xuzhou; 2: Nantong; 3: Wuxi.

ACF: autocorrelation function; PACF: partial autocorrelation function.

**Additional Figure S2. Time series plots of the six meteorological factors in the three cities between 2005 and 2017.**

**Additional Figure S3. Epoch-error plots of the optimal RNN models of the three cities after three training cycles.**

a: Xuzhou; b: Nantong; c: Wuxi; 1: first training cycle; 2: second training cycle; 3: third training cycle.

**Additional File 1. R code.**

**Additional Table S1. Description of monthly meteorological factors in the three cities between 2005 and 2017.**

| City | Variables | Mean | SD | Min | P25 | P50 | P75 | Max |
| --- | --- | --- | --- | --- | --- | --- | --- | --- |
| Xuzhou | Average temperature (℃) | 15.39 | 9.53 | -2.00 | 6.38 | 16.65 | 24.05 | 30.00 |
|  | Average atmospheric pressure (hPa) | 1011.77 | 8.48 | 997.10 | 1003.35 | 1012.80 | 1019.40 | 1027.10 |
|  | Average relative humidity (%) | 1.89 | 0.40 | 1.00 | 1.60 | 1.80 | 2.20 | 2.90 |
|  | Average wind speed (m/s) | 66.47 | 9.49 | 41.00 | 59.00 | 67.00 | 74.00 | 86.00 |
|  | Precipitation (mm) | 67.79 | 76.55 | 0.00 | 14.93 | 38.90 | 97.40 | 526.90 |
|  | Sunshine time (h) | 177.83 | 48.52 | 50.70 | 142.55 | 180.45 | 213.33 | 293.50 |
| Nantong | Average temperature (℃) | 16.21 | 8.76 | -0.40 | 7.80 | 17.00 | 23.58 | 31.20 |
|  | Average atmospheric pressure (hPa) | 1016.02 | 8.08 | 1002.90 | 1008.08 | 1017.45 | 1023.15 | 1030.60 |
|  | Average relative humidity (%) | 2.69 | 0.54 | 1.00 | 2.40 | 2.75 | 3.10 | 4.00 |
|  | Average wind speed (m/s) | 76.34 | 5.90 | 59.00 | 73.00 | 76.50 | 81.00 | 90.00 |
|  | Precipitation (mm) | 104.08 | 101.57 | 1.10 | 35.20 | 69.90 | 134.95 | 475.80 |
|  | Sunshine time (h) | 157.11 | 46.55 | 35.20 | 123.85 | 154.15 | 185.78 | 301.10 |
| Wuxi | Average temperature (℃) | 17.08 | 8.83 | 0.60 | 9.03 | 18.10 | 24.50 | 32.20 |
|  | Average atmospheric pressure (hPa) | 1016.07 | 8.21 | 1002.50 | 1007.98 | 1017.55 | 1023.25 | 1031.00 |
|  | Average relative humidity (%) | 2.44 | 0.38 | 1.60 | 2.16 | 2.40 | 2.70 | 3.60 |
|  | Average wind speed (m/s) | 71.43 | 6.44 | 54.00 | 67.25 | 72.00 | 76.00 | 86.00 |
|  | Precipitation (mm) | 103.41 | 92.20 | 4.00 | 42.68 | 75.85 | 133.68 | 598.50 |
|  | Sunshine time (h) | 152.64 | 45.14 | 53.00 | 118.00 | 147.25 | 180.70 | 291.10 |

**Additional Table S2. Alternative ARIMA models for the three cities.**

| City | Model | Normalized BIC value | *P*^*^ | The parameters were all significant |
| --- | --- | --- | --- | --- |
| Xuzhou | ARIMA (0,1,1)(0,1,1)_12_ | 8.863 | 0.316 | Yes |
|  | ARIMA (1,1,0)(0,1,1)_12_ | 8.882 | 0.276 | Yes |
|  | ARIMA (1,1,1)(0,1,1)_12_ | 8.857 | 0.861 | Yes |
|  | ARIMA (0,1,1)(1,1,0)_12_ | 8.936 | 0.653 | Yes |
|  | ARIMA (1,1,0)(1,1,0)_12_ | 8.955 | 0.498 | Yes |
|  | ARIMA (1,1,1)(1,1,0)_12_ | 8.952 | 0.804 | No |
|  | ARIMA (0,1,1)(1,1,1)_12_ | 8.912 | 0.146 | Yes |
|  | ARIMA (1,1,0)(1,1,1)_12_ | 8.928 | 0.106 | Yes |
|  | ARIMA (1,1,1)(1,1,1)_12_ | 8.910 | 0.549 | Yes |
| Nantong | ARIMA (0,1,1)(0,1,1)_12_ | 8.609 | 0.433 | Yes |
|  | ARIMA (1,1,0)(0,1,1)_12_ | 8.679 | 0.106 | Yes |
|  | ARIMA (1,1,1)(0,1,1)_12_ | 8.632 | 0.508 | Yes |
|  | ARIMA (2,1,0)(0,1,1)_12_ | 8.661 | 0.519 | Yes |
|  | ARIMA (2,1,1)(0,1,1)_12_ | 8.668 | 0.325 | No |
|  | ARIMA (0,1,1)(1,1,0)_12_ | 8.643 | 0.739 | Yes |
|  | ARIMA (1,1,0)(1,1,0)_12_ | 8.719 | 0.125 | Yes |
|  | ARIMA (1,1,1)(1,1,0)_12_ | 8.667 | 0.300 | Yes |
|  | ARIMA (2,1,0)(1,1,0)_12_ | 8.701 | 0.669 | Yes |
|  | ARIMA (2,1,1)(1,1,0)_12_ | 8.696 | 0.313 | No |
|  | ARIMA (0,1,1)(1,1,1)_12_ | 8.649 | 0.367 | No |
|  | ARIMA (1,1,0)(1,1,1)_12_ | 8.721 | 0.078 | No |
|  | ARIMA (1,1,1)(1,1,1)_12_ | 8.666 | 0.452 | No |
|  | ARIMA (2,1,0)(1,1,1)_12_ | 8.702 | 0.455 | No |
|  | ARIMA (2,1,1)(1,1,1)_12_ | 8.703 | 0.305 | No |
| Wuxi | ARIMA (0,1,1)(0,1,1)_12_ | 6.933 | 0.176 | Yes |
|  | ARIMA (1,1,0)(0,1,1)_12_ | 7.088 | 0.005 | Yes |
|  | ARIMA (1,1,1)(0,1,1)_12_ | 6.958 | 0.256 | No |

BIC: Bayesian information criterion.

^*^: The Ljung-Box test.

**Additional Table S3. The spearman rank correlation coefficients between the monthly number of PTB cases and meteorological factors in the three cities.**

| Variable | MAT1 | MAP1 | MAS1 | MAH1 | MP1 | MST1 | MAT2 | MAP2 | MAS2 | MAH2 | MP2 | MST2 | MAT3 | MAP3 | MAS3 | MAH3 | MP3 | MST3 |
| --- | --- | --- | --- | --- | --- | --- | --- | --- | --- | --- | --- | --- | --- | --- | --- | --- | --- | --- |
| PTB^α^ | 0.035 | -0.115 | 0.536^#^ | -0.025 | 0.004 | -0.153 | -0.115 | 0.017 | 0.519^#^ | -0.083 | -0.075 | -0.244^#^ | -0.227^#^ | 0.137 | 0.450^#^ | -0.133 | -0.178^*^ | -0.262^#^ |
| PTB^β^ | 0.068 | -0.090 | -0.177^*^ | -0.199^*^ | -0.054 | 0.114 | -0.043 | -0.007 | -0.219^#^ | -0.229^#^ | -0.117 | 0.098 | -0.103 | 0.061 | -0.240^#^ | -0.184^*^ | -0.114 | 0.042 |
| PTB^γ^ | 0.216^#^ | -0.297^#^ | 0.461^#^ | -0.164^*^ | 0.099 | 0.168^*^ | 0.011 | -0.132 | 0.398^#^ | -0.155 | 0.053 | 0.100 | -0.160^*^ | 0.035 | 0.425^#^ | -0.193^*^ | -0.065 | 0.060 |

MAT: monthly average temperature; MAP: monthly average atmospheric pressure; MAS: monthly average wind speed; MAH: monthly average relative humidity; MP: monthly precipitation; MST: monthly sunshine time; 1: 1 month ago; 2: 2 months ago; 3: 3 months ago.

^α^: the number of PTB cases in the current month in Xuzhou.

^β^: the number of PTB cases in the current month in Nantong.

^γ^: the number of PTB cases in the current month in Wuxi.

^*^: *P* <0.05.

^#^: *P* <0.01.

**Additional Table S4. The detailed composition of the nine RNN models.**

| Model | Training set | | |  | Testing set |  | Predicting set |
| --- | --- | --- | --- | --- | --- | --- | --- |
|  | Input |  | Output |  | Input |  | Input |
| RNN1 | First group: NPTBC in Jan. 2005;  Last group: NPTBC in Nov. 2016. |  | First group: NPTBC in Feb. 2005;  Last group: NPTBC in Dec. 2016. |  | First group: NPTBC in Dec. 2016;  Last group: NPTBC in Nov. 2017. |  | First group: NPTBC in Dec. 2017;  Last group: NPTBC in Nov. 2018. |
| RNN2 | First group: NPTBC from Jan. to Feb. 2005;  Last group: NPTBC from Oct. to Nov. 2016. |  | First group: NPTBC in Mar. 2005;  Last group: NPTBC in Dec. 2016. |  | First group: NPTBC from Nov. to Dec. 2016;  Last group: NPTBC from Oct. to Nov. 2017. |  | First group: NPTBC from Nov. to Dec. 2017;  Last group: NPTBC from Oct. to Nov. 2018. |
| RNN3 | First group: NPTBC from Jan. to Mar. 2005;  Last group: NPTBC from Sep. to Nov. 2016. |  | First group: NPTBC in Apr. 2005;  Last group: NPTBC in Dec. 2016. |  | First group: NPTBC from Oct. to Dec. 2016;  Last group: NPTBC from Sep. to Nov. 2017. |  | First group: NPTBC from Oct. to Dec. 2017;  Last group: NPTBC from Sep. to Nov. 2018. |
| RNN4 | First group: NPTBC from Jan. to Jun. 2005;  Last group: NPTBC from Jun. to Nov. 2016. |  | First group: NPTBC in Jul. 2005;  Last group: NPTBC in Dec. 2016. |  | First group: NPTBC from Jul. to Dec. 2016;  Last group: NPTBC from Jun. to Nov. 2017. |  | First group: NPTBC from Jul. to Dec. 2017;  Last group: NPTBC from Jun. to Nov. 2018. |
| RNN5 | First group: NPTBC from Jan. to Dec. 2005;  Last group: NPTBC from Dec. 2015 to Nov. 2016. |  | First group: NPTBC in Jan. 2006;  Last group: NPTBC in Dec. 2016. |  | First group: NPTBC from Jan. to Dec. 2016;  Last group: NPTBC from Dec. 2016 to Nov. 2017. |  | First group: NPTBC from Jan. to Dec. 2017;  Last group: NPTBC from Dec. 2017 to Nov. 2018. |
| RNN6 | First group: NPTBC from Jan. to Dec. 2005 and MF in Dec. 2005;  Last group: NPTBC from Dec. 2015 to Nov. 2016 and MF in Nov. 2016. |  | First group: NPTBC in Jan. 2006;  Last group: NPTBC in Dec. 2016. |  | First group: NPTBC from Jan. to Dec. 2016 and MF in Dec. 2016;  Last group: NPTBC from Dec. 2016 to Nov. 2017 and MF in Nov. 2017. |  | First group: NPTBC from Jan. to Dec. 2017 and MF in Dec. 2017;  Last group: NPTBC from Dec. 2017 to Nov. 2018 and MF in Nov. 2018. |
| RNN7 | First group: NPTBC from Jan. to Dec. 2005 and MF in Nov. 2005;  Last group: NPTBC from Dec. 2015 to Nov. 2016 and MF in Oct. 2016. |  | First group: NPTBC in Jan. 2006;  Last group: NPTBC in Dec. 2016. |  | First group: NPTBC from Jan. to Dec. 2016 and MF in Nov. 2016;  Last group: NPTBC from Dec. 2016 to Nov. 2017 and MF in Oct. 2017. |  | First group: NPTBC from Jan. to Dec. 2017 and MF in Nov. 2017;  Last group: NPTBC from Dec. 2017 to Nov. 2018 and MF in Oct. 2018. |
| RNN8 | First group: NPTBC from Jan. to Dec. 2005 and MF in Oct. 2005;  Last group: NPTBC from Dec. 2015 to Nov. 2016 and MF in Sep. 2016. |  | First group: NPTBC in Jan. 2006;  Last group: NPTBC in Dec. 2016. |  | First group: NPTBC from Jan. to Dec. 2016 and MF in Oct. 2016;  Last group: NPTBC from Dec. 2016 to Nov. 2017 and MF in Sep. 2017. |  | First group: NPTBC from Jan. to Dec. 2017 and MF in Oct. 2017;  Last group: NPTBC from Dec. 2017 to Nov. 2018 and MF in Sep. 2018. |
| RNN9 | First group: NPTBC from Jan. to Dec. 2005 and MF from Oct. to Dec. 2005;  Last group: NPTBC from Dec. 2015 to Nov. 2016 and MF from Sep. to Nov. 2016. |  | First group: NPTBC in Jan. 2006;  Last group: NPTBC in Dec. 2016. |  | First group: NPTBC from Jan. to Dec. 2016 and MF from Oct. to Dec. 2016;  Last group: NPTBC from Dec. 2016 to Nov. 2017 and MF from Sep. to Nov. 2017. |  | First group: NPTBC from Jan. to Dec. 2017 and MF from Oct. to Dec. 2017;  Last group: NPTBC from Dec. 2017 to Nov. 2018 and MF from Sep. to Nov. 2018. |

NPTBC: number of PTB cases; MF: meteorological factors significantly correlated with PTB.





**Additional Figure S1. ACF and PACF plots.**

a: ACF plots of the monthly number of PTB cases after one ordinary difference and one seasonal difference in the three cities; b: PACF plots of the monthly number of PTB cases after one ordinary difference and one seasonal difference in the three cities; c: ACF plots of the residual series of the optimal ARIMA models of the three cities; d: PACF plots of the residual series of the optimal ARIMA models of the three cities; 1: Xuzhou; 2: Nantong; 3: Wuxi.

ACF: autocorrelation function; PACF: partial autocorrelation function.





**Additional Figure S2. Time series plots of the six meteorological factors in the three cities between 2005 and 2017.**





**Additional Figure S3. Epoch-error plots of the optimal RNN models of the three cities after three training cycles.**

a: Xuzhou; b: Nantong; c: Wuxi; 1: first training cycle; 2: second training cycle; 3: third training cycle.

**Additional File 1. R code.**

library(rnn)

##Xuzhou

#1

x1<-xzrnn1$x1

y<-xzrnn1$y

max<-max(x1,y)

min<-min(x1,y)

x1n<-((x1-min)/(max-min))

yn<-((y-min)/(max-min))

xf1n<-x1n[1:143]

yfn<-yn[1:143]

xt1n<-x1n[144:155]

yta<-y[144:155]

xp1n<-x1n[156:167]

ypa<-y[156:167]

xf<-array(xf1n,dim = c(143,1,1))

yf<-array(yfn,dim = c(143,1,1))

xt<-array(xt1n,dim = c(12,1,1))

xp<-array(xp1n,dim = c(12,1,1))

model1.1<-trainr(yf,xf,learningrate = 0.05,hidden_dim = 3,numepochs = 500,batch_size = 10,network_type = "rnn")

par(mfrow=c(1,1))

plot(colMeans(model1.1$error),type = 'l',xlab = 'Epoch',ylab = 'Errors')

ytn<-(predictr(model1.1,xt))

yt<-round(ytn*(max-min)+min)

yt

mapet<-mean(abs((yta-yt)/yta*100))

mapet

ypn<-(predictr(model1.1,xp))

yp<-round(ypn*(max-min)+min)

yp

mapep<-mean(abs((ypa-yp)/ypa*100))

mapep

model1.2<-trainr(yf,xf,learningrate = 0.05,hidden_dim = 5,numepochs = 400,batch_size = 10,network_type = "rnn")

par(mfrow=c(1,1))

plot(colMeans(model1.2$error),type = 'l',xlab = 'Epoch',ylab = 'Errors')

ytn<-(predictr(model1.2,xt))

yt<-round(ytn*(max-min)+min)

yt

mapet<-mean(abs((yta-yt)/yta*100))

mapet

ypn<-(predictr(model1.2,xp))

yp<-round(ypn*(max-min)+min)

yp

mapep<-mean(abs((ypa-yp)/ypa*100))

mapep

model1.3<-trainr(yf,xf,learningrate = 0.05,hidden_dim = 10,numepochs = 250,batch_size = 10,network_type = "rnn")

par(mfrow=c(1,1))

plot(colMeans(model1.3$error),type = 'l',xlab = 'Epoch',ylab = 'Errors')

ytn<-(predictr(model1.3,xt))

yt<-round(ytn*(max-min)+min)

yt

mapet<-mean(abs((yta-yt)/yta*100))

mapet

ypn<-(predictr(model1.3,xp))

yp<-round(ypn*(max-min)+min)

yp

mapep<-mean(abs((ypa-yp)/ypa*100))

mapep

model1.4<-trainr(yf,xf,learningrate = 0.1,hidden_dim = 3,numepochs = 200,batch_size = 10,network_type = "rnn")

par(mfrow=c(1,1))

plot(colMeans(model1.4$error),type = 'l',xlab = 'Epoch',ylab = 'Errors')

ytn<-(predictr(model1.4,xt))

yt<-round(ytn*(max-min)+min)

yt

mapet<-mean(abs((yta-yt)/yta*100))

mapet

ypn<-(predictr(model1.4,xp))

yp<-round(ypn*(max-min)+min)

yp

mapep<-mean(abs((ypa-yp)/ypa*100))

mapep

model1.5<-trainr(yf,xf,learningrate = 0.1,hidden_dim = 5,numepochs = 150,batch_size = 10,network_type = "rnn")

par(mfrow=c(1,1))

plot(colMeans(model1.5$error),type = 'l',xlab = 'Epoch',ylab = 'Errors')

ytn<-(predictr(model1.5,xt))

yt<-round(ytn*(max-min)+min)

yt

mapet<-mean(abs((yta-yt)/yta*100))

mapet

ypn<-(predictr(model1.5,xp))

yp<-round(ypn*(max-min)+min)

yp

mapep<-mean(abs((ypa-yp)/ypa*100))

mapep

model1.6<-trainr(yf,xf,learningrate = 0.1,hidden_dim = 10,numepochs = 100,batch_size = 10,network_type = "rnn")

par(mfrow=c(1,1))

plot(colMeans(model1.6$error),type = 'l',xlab = 'Epoch',ylab = 'Errors')

ytn<-(predictr(model1.6,xt))

yt<-round(ytn*(max-min)+min)

yt

mapet<-mean(abs((yta-yt)/yta*100))

mapet

ypn<-(predictr(model1.6,xp))

yp<-round(ypn*(max-min)+min)

yp

mapep<-mean(abs((ypa-yp)/ypa*100))

mapep

model1.7<-trainr(yf,xf,learningrate = 0.2,hidden_dim = 3,numepochs = 100,batch_size = 10,network_type = "rnn")

par(mfrow=c(1,1))

plot(colMeans(model1.7$error),type = 'l',xlab = 'Epoch',ylab = 'Errors')

ytn<-(predictr(model1.7,xt))

yt<-round(ytn*(max-min)+min)

yt

mapet<-mean(abs((yta-yt)/yta*100))

mapet

ypn<-(predictr(model1.7,xp))

yp<-round(ypn*(max-min)+min)

yp

mapep<-mean(abs((ypa-yp)/ypa*100))

mapep

model1.8<-trainr(yf,xf,learningrate = 0.2,hidden_dim = 5,numepochs = 80,batch_size = 10,network_type = "rnn")

par(mfrow=c(1,1))

plot(colMeans(model1.8$error),type = 'l',xlab = 'Epoch',ylab = 'Errors')

ytn<-(predictr(model1.8,xt))

yt<-round(ytn*(max-min)+min)

yt

mapet<-mean(abs((yta-yt)/yta*100))

mapet

ypn<-(predictr(model1.8,xp))

yp<-round(ypn*(max-min)+min)

yp

mapep<-mean(abs((ypa-yp)/ypa*100))

mapep

model1.9<-trainr(yf,xf,learningrate = 0.2,hidden_dim = 10,numepochs = 60,batch_size = 10,network_type = "rnn")

par(mfrow=c(1,1))

plot(colMeans(model1.9$error),type = 'l',xlab = 'Epoch',ylab = 'Errors')

ytn<-(predictr(model1.9,xt))

yt<-round(ytn*(max-min)+min)

yt

mapet<-mean(abs((yta-yt)/yta*100))

mapet

ypn<-(predictr(model1.9,xp))

yp<-round(ypn*(max-min)+min)

yp

mapep<-mean(abs((ypa-yp)/ypa*100))

mapep

#2

x1<-xzrnn2$x1

x2<-xzrnn2$x2

y<-xzrnn2$y

max<-max(x1,x2,y)

min<-min(x1,x2,y)

x1n<-((x1-min)/(max-min))

x2n<-((x2-min)/(max-min))

yn<-((y-min)/(max-min))

xf1n<-x1n[1:142]

xf2n<-x2n[1:142]

yfn<-yn[1:142]

xt1n<-x1n[143:154]

xt2n<-x2n[143:154]

yta<-y[143:154]

xp1n<-x1n[155:166]

xp2n<-x2n[155:166]

ypa<-y[155:166]

xf<-array(c(xf1n,xf2n),dim = c(142,1,2))

yf<-array(yfn,dim = c(142,1,1))

xt<-array(c(xt1n,xt2n),dim = c(12,1,2))

xp<-array(c(xp1n,xp2n),dim = c(12,1,2))

model2.1<-trainr(yf,xf,learningrate = 0.05,hidden_dim = 3,numepochs = 500,batch_size = 10,network_type = "rnn")

par(mfrow=c(1,1))

plot(colMeans(model2.1$error),type = 'l',xlab = 'Epoch',ylab = 'Errors')

ytn<-(predictr(model2.1,xt))

yt<-round(ytn*(max-min)+min)

yt

mapet<-mean(abs((yta-yt)/yta*100))

mapet

ypn<-(predictr(model2.1,xp))

yp<-round(ypn*(max-min)+min)

yp

mapep<-mean(abs((ypa-yp)/ypa*100))

mapep

model2.2<-trainr(yf,xf,learningrate = 0.05,hidden_dim = 5,numepochs = 400,batch_size = 10,network_type = "rnn")

par(mfrow=c(1,1))

plot(colMeans(model2.2$error),type = 'l',xlab = 'Epoch',ylab = 'Errors')

ytn<-(predictr(model2.2,xt))

yt<-round(ytn*(max-min)+min)

yt

mapet<-mean(abs((yta-yt)/yta*100))

mapet

ypn<-(predictr(model2.2,xp))

yp<-round(ypn*(max-min)+min)

yp

mapep<-mean(abs((ypa-yp)/ypa*100))

mapep

model2.3<-trainr(yf,xf,learningrate = 0.05,hidden_dim = 10,numepochs = 250,batch_size = 10,network_type = "rnn")

par(mfrow=c(1,1))

plot(colMeans(model2.3$error),type = 'l',xlab = 'Epoch',ylab = 'Errors')

ytn<-(predictr(model2.3,xt))

yt<-round(ytn*(max-min)+min)

yt

mapet<-mean(abs((yta-yt)/yta*100))

mapet

ypn<-(predictr(model2.3,xp))

yp<-round(ypn*(max-min)+min)

yp

mapep<-mean(abs((ypa-yp)/ypa*100))

mapep

model2.4<-trainr(yf,xf,learningrate = 0.1,hidden_dim = 3,numepochs = 200,batch_size = 10,network_type = "rnn")

par(mfrow=c(1,1))

plot(colMeans(model2.4$error),type = 'l',xlab = 'Epoch',ylab = 'Errors')

ytn<-(predictr(model2.4,xt))

yt<-round(ytn*(max-min)+min)

yt

mapet<-mean(abs((yta-yt)/yta*100))

mapet

ypn<-(predictr(model2.4,xp))

yp<-round(ypn*(max-min)+min)

yp

mapep<-mean(abs((ypa-yp)/ypa*100))

mapep

model2.5<-trainr(yf,xf,learningrate = 0.1,hidden_dim = 5,numepochs = 150,batch_size = 10,network_type = "rnn")

par(mfrow=c(1,1))

plot(colMeans(model2.5$error),type = 'l',xlab = 'Epoch',ylab = 'Errors')

ytn<-(predictr(model2.5,xt))

yt<-round(ytn*(max-min)+min)

yt

mapet<-mean(abs((yta-yt)/yta*100))

mapet

ypn<-(predictr(model2.5,xp))

yp<-round(ypn*(max-min)+min)

yp

mapep<-mean(abs((ypa-yp)/ypa*100))

mapep

model2.6<-trainr(yf,xf,learningrate = 0.1,hidden_dim = 10,numepochs = 100,batch_size = 10,network_type = "rnn")

par(mfrow=c(1,1))

plot(colMeans(model2.6$error),type = 'l',xlab = 'Epoch',ylab = 'Errors')

ytn<-(predictr(model2.6,xt))

yt<-round(ytn*(max-min)+min)

yt

mapet<-mean(abs((yta-yt)/yta*100))

mapet

ypn<-(predictr(model2.6,xp))

yp<-round(ypn*(max-min)+min)

yp

mapep<-mean(abs((ypa-yp)/ypa*100))

mapep

model2.7<-trainr(yf,xf,learningrate = 0.2,hidden_dim = 3,numepochs = 100,batch_size = 10,network_type = "rnn")

par(mfrow=c(1,1))

plot(colMeans(model2.7$error),type = 'l',xlab = 'Epoch',ylab = 'Errors')

ytn<-(predictr(model2.7,xt))

yt<-round(ytn*(max-min)+min)

yt

mapet<-mean(abs((yta-yt)/yta*100))

mapet

ypn<-(predictr(model2.7,xp))

yp<-round(ypn*(max-min)+min)

yp

mapep<-mean(abs((ypa-yp)/ypa*100))

mapep

model2.8<-trainr(yf,xf,learningrate = 0.2,hidden_dim = 5,numepochs = 80,batch_size = 10,network_type = "rnn")

par(mfrow=c(1,1))

plot(colMeans(model2.8$error),type = 'l',xlab = 'Epoch',ylab = 'Errors')

ytn<-(predictr(model2.8,xt))

yt<-round(ytn*(max-min)+min)

yt

mapet<-mean(abs((yta-yt)/yta*100))

mapet

ypn<-(predictr(model2.8,xp))

yp<-round(ypn*(max-min)+min)

yp

mapep<-mean(abs((ypa-yp)/ypa*100))

mapep

model2.9<-trainr(yf,xf,learningrate = 0.2,hidden_dim = 10,numepochs = 60,batch_size = 10,network_type = "rnn")

par(mfrow=c(1,1))

plot(colMeans(model2.9$error),type = 'l',xlab = 'Epoch',ylab = 'Errors')

ytn<-(predictr(model2.9,xt))

yt<-round(ytn*(max-min)+min)

yt

mapet<-mean(abs((yta-yt)/yta*100))

mapet

ypn<-(predictr(model2.9,xp))

yp<-round(ypn*(max-min)+min)

yp

mapep<-mean(abs((ypa-yp)/ypa*100))

mapep

#3

x1<-xzrnn3$x1

x2<-xzrnn3$x2

x3<-xzrnn3$x3

y<-xzrnn3$y

max<-max(x1,x2,x3,y)

min<-min(x1,x2,x3,y)

x1n<-((x1-min)/(max-min))

x2n<-((x2-min)/(max-min))

x3n<-((x3-min)/(max-min))

yn<-((y-min)/(max-min))

xf1n<-x1n[1:141]

xf2n<-x2n[1:141]

xf3n<-x3n[1:141]

yfn<-yn[1:141]

xt1n<-x1n[142:153]

xt2n<-x2n[142:153]

xt3n<-x3n[142:153]

yta<-y[142:153]

xp1n<-x1n[154:165]

xp2n<-x2n[154:165]

xp3n<-x3n[154:165]

ypa<-y[154:165]

xf<-array(c(xf1n,xf2n,xf3n),dim = c(141,1,3))

yf<-array(yfn,dim = c(141,1,1))

xt<-array(c(xt1n,xt2n,xt3n),dim = c(12,1,3))

xp<-array(c(xp1n,xp2n,xp3n),dim = c(12,1,3))

model3.1<-trainr(yf,xf,learningrate = 0.05,hidden_dim = 3,numepochs = 500,batch_size = 10,network_type = "rnn")

par(mfrow=c(1,1))

plot(colMeans(model3.1$error),type = 'l',xlab = 'Epoch',ylab = 'Errors')

ytn<-(predictr(model3.1,xt))

yt<-round(ytn*(max-min)+min)

yt

mapet<-mean(abs((yta-yt)/yta*100))

mapet

ypn<-(predictr(model3.1,xp))

yp<-round(ypn*(max-min)+min)

yp

mapep<-mean(abs((ypa-yp)/ypa*100))

mapep

model3.2<-trainr(yf,xf,learningrate = 0.05,hidden_dim = 5,numepochs = 400,batch_size = 10,network_type = "rnn")

par(mfrow=c(1,1))

plot(colMeans(model3.2$error),type = 'l',xlab = 'Epoch',ylab = 'Errors')

ytn<-(predictr(model3.2,xt))

yt<-round(ytn*(max-min)+min)

yt

mapet<-mean(abs((yta-yt)/yta*100))

mapet

ypn<-(predictr(model3.2,xp))

yp<-round(ypn*(max-min)+min)

yp

mapep<-mean(abs((ypa-yp)/ypa*100))

mapep

model3.3<-trainr(yf,xf,learningrate = 0.05,hidden_dim = 10,numepochs = 300,batch_size = 10,network_type = "rnn")

par(mfrow=c(1,1))

plot(colMeans(model3.3$error),type = 'l',xlab = 'Epoch',ylab = 'Errors')

ytn<-(predictr(model3.3,xt))

yt<-round(ytn*(max-min)+min)

yt

mapet<-mean(abs((yta-yt)/yta*100))

mapet

ypn<-(predictr(model3.3,xp))

yp<-round(ypn*(max-min)+min)

yp

mapep<-mean(abs((ypa-yp)/ypa*100))

mapep

model3.4<-trainr(yf,xf,learningrate = 0.1,hidden_dim = 3,numepochs = 250,batch_size = 10,network_type = "rnn")

par(mfrow=c(1,1))

plot(colMeans(model3.4$error),type = 'l',xlab = 'Epoch',ylab = 'Errors')

ytn<-(predictr(model3.4,xt))

yt<-round(ytn*(max-min)+min)

yt

mapet<-mean(abs((yta-yt)/yta*100))

mapet

ypn<-(predictr(model3.4,xp))

yp<-round(ypn*(max-min)+min)

yp

mapep<-mean(abs((ypa-yp)/ypa*100))

mapep

model3.5<-trainr(yf,xf,learningrate = 0.1,hidden_dim = 5,numepochs = 200,batch_size = 10,network_type = "rnn")

par(mfrow=c(1,1))

plot(colMeans(model3.5$error),type = 'l',xlab = 'Epoch',ylab = 'Errors')

ytn<-(predictr(model3.5,xt))

yt<-round(ytn*(max-min)+min)

yt

mapet<-mean(abs((yta-yt)/yta*100))

mapet

ypn<-(predictr(model3.5,xp))

yp<-round(ypn*(max-min)+min)

yp

mapep<-mean(abs((ypa-yp)/ypa*100))

mapep

model3.6<-trainr(yf,xf,learningrate = 0.1,hidden_dim = 10,numepochs = 150,batch_size = 10,network_type = "rnn")

par(mfrow=c(1,1))

plot(colMeans(model3.6$error),type = 'l',xlab = 'Epoch',ylab = 'Errors')

ytn<-(predictr(model3.6,xt))

yt<-round(ytn*(max-min)+min)

yt

mapet<-mean(abs((yta-yt)/yta*100))

mapet

ypn<-(predictr(model3.6,xp))

yp<-round(ypn*(max-min)+min)

yp

mapep<-mean(abs((ypa-yp)/ypa*100))

mapep

model3.7<-trainr(yf,xf,learningrate = 0.2,hidden_dim = 3,numepochs = 150,batch_size = 10,network_type = "rnn")

par(mfrow=c(1,1))

plot(colMeans(model3.7$error),type = 'l',xlab = 'Epoch',ylab = 'Errors')

ytn<-(predictr(model3.7,xt))

yt<-round(ytn*(max-min)+min)

yt

mapet<-mean(abs((yta-yt)/yta*100))

mapet

ypn<-(predictr(model3.7,xp))

yp<-round(ypn*(max-min)+min)

yp

mapep<-mean(abs((ypa-yp)/ypa*100))

mapep

model3.8<-trainr(yf,xf,learningrate = 0.2,hidden_dim = 5,numepochs = 100,batch_size = 10,network_type = "rnn")

par(mfrow=c(1,1))

plot(colMeans(model3.8$error),type = 'l',xlab = 'Epoch',ylab = 'Errors')

ytn<-(predictr(model3.8,xt))

yt<-round(ytn*(max-min)+min)

yt

mapet<-mean(abs((yta-yt)/yta*100))

mapet

ypn<-(predictr(model3.8,xp))

yp<-round(ypn*(max-min)+min)

yp

mapep<-mean(abs((ypa-yp)/ypa*100))

mapep

model3.9<-trainr(yf,xf,learningrate = 0.2,hidden_dim = 10,numepochs = 80,batch_size = 10,network_type = "rnn")

par(mfrow=c(1,1))

plot(colMeans(model3.9$error),type = 'l',xlab = 'Epoch',ylab = 'Errors')

ytn<-(predictr(model3.9,xt))

yt<-round(ytn*(max-min)+min)

yt

mapet<-mean(abs((yta-yt)/yta*100))

mapet

ypn<-(predictr(model3.9,xp))

yp<-round(ypn*(max-min)+min)

yp

mapep<-mean(abs((ypa-yp)/ypa*100))

mapep

#4

x1<-xzrnn4$x1

x2<-xzrnn4$x2

x3<-xzrnn4$x3

x4<-xzrnn4$x4

x5<-xzrnn4$x5

x6<-xzrnn4$x6

y<-xzrnn4$y

max<-max(x1,x2,x3,x4,x5,x6,y)

min<-min(x1,x2,x3,x4,x5,x6,y)

x1n<-((x1-min)/(max-min))

x2n<-((x2-min)/(max-min))

x3n<-((x3-min)/(max-min))

x4n<-((x4-min)/(max-min))

x5n<-((x5-min)/(max-min))

x6n<-((x6-min)/(max-min))

yn<-((y-min)/(max-min))

xf1n<-x1n[1:138]

xf2n<-x2n[1:138]

xf3n<-x3n[1:138]

xf4n<-x4n[1:138]

xf5n<-x5n[1:138]

xf6n<-x6n[1:138]

yfn<-yn[1:138]

xt1n<-x1n[139:150]

xt2n<-x2n[139:150]

xt3n<-x3n[139:150]

xt4n<-x4n[139:150]

xt5n<-x5n[139:150]

xt6n<-x6n[139:150]

yta<-y[139:150]

xp1n<-x1n[151:162]

xp2n<-x2n[151:162]

xp3n<-x3n[151:162]

xp4n<-x4n[151:162]

xp5n<-x5n[151:162]

xp6n<-x6n[151:162]

ypa<-y[151:162]

xf<-array(c(xf1n,xf2n,xf3n,xf4n,xf5n,xf6n),dim = c(138,1,6))

yf<-array(yfn,dim = c(138,1,1))

xt<-array(c(xt1n,xt2n,xt3n,xt4n,xt5n,xt6n),dim = c(12,1,6))

xp<-array(c(xp1n,xp2n,xp3n,xp4n,xp5n,xp6n),dim = c(12,1,6))

model4.1<-trainr(yf,xf,learningrate = 0.05,hidden_dim = 3,numepochs = 600,batch_size = 10,network_type = "rnn")

par(mfrow=c(1,1))

plot(colMeans(model4.1$error),type = 'l',xlab = 'Epoch',ylab = 'Errors')

ytn<-(predictr(model4.1,xt))

yt<-round(ytn*(max-min)+min)

yt

mapet<-mean(abs((yta-yt)/yta*100))

mapet

ypn<-(predictr(model4.1,xp))

yp<-round(ypn*(max-min)+min)

yp

mapep<-mean(abs((ypa-yp)/ypa*100))

mapep

model4.2<-trainr(yf,xf,learningrate = 0.05,hidden_dim = 5,numepochs = 500,batch_size = 10,network_type = "rnn")

par(mfrow=c(1,1))

plot(colMeans(model4.2$error),type = 'l',xlab = 'Epoch',ylab = 'Errors')

ytn<-(predictr(model4.2,xt))

yt<-round(ytn*(max-min)+min)

yt

mapet<-mean(abs((yta-yt)/yta*100))

mapet

ypn<-(predictr(model4.2,xp))

yp<-round(ypn*(max-min)+min)

yp

mapep<-mean(abs((ypa-yp)/ypa*100))

mapep

model4.3<-trainr(yf,xf,learningrate = 0.05,hidden_dim = 10,numepochs = 400,batch_size = 10,network_type = "rnn")

par(mfrow=c(1,1))

plot(colMeans(model4.3$error),type = 'l',xlab = 'Epoch',ylab = 'Errors')

ytn<-(predictr(model4.3,xt))

yt<-round(ytn*(max-min)+min)

yt

mapet<-mean(abs((yta-yt)/yta*100))

mapet

ypn<-(predictr(model4.3,xp))

yp<-round(ypn*(max-min)+min)

yp

mapep<-mean(abs((ypa-yp)/ypa*100))

mapep

model4.4<-trainr(yf,xf,learningrate = 0.1,hidden_dim = 3,numepochs = 250,batch_size = 10,network_type = "rnn")

par(mfrow=c(1,1))

plot(colMeans(model4.4$error),type = 'l',xlab = 'Epoch',ylab = 'Errors')

ytn<-(predictr(model4.4,xt))

yt<-round(ytn*(max-min)+min)

yt

mapet<-mean(abs((yta-yt)/yta*100))

mapet

ypn<-(predictr(model4.4,xp))

yp<-round(ypn*(max-min)+min)

yp

mapep<-mean(abs((ypa-yp)/ypa*100))

mapep

model4.5<-trainr(yf,xf,learningrate = 0.1,hidden_dim = 5,numepochs = 200,batch_size = 10,network_type = "rnn")

par(mfrow=c(1,1))

plot(colMeans(model4.5$error),type = 'l',xlab = 'Epoch',ylab = 'Errors')

ytn<-(predictr(model4.5,xt))

yt<-round(ytn*(max-min)+min)

yt

mapet<-mean(abs((yta-yt)/yta*100))

mapet

ypn<-(predictr(model4.5,xp))

yp<-round(ypn*(max-min)+min)

yp

mapep<-mean(abs((ypa-yp)/ypa*100))

mapep

model4.6<-trainr(yf,xf,learningrate = 0.1,hidden_dim = 10,numepochs = 150,batch_size = 10,network_type = "rnn")

par(mfrow=c(1,1))

plot(colMeans(model4.6$error),type = 'l',xlab = 'Epoch',ylab = 'Errors')

ytn<-(predictr(model4.6,xt))

yt<-round(ytn*(max-min)+min)

yt

mapet<-mean(abs((yta-yt)/yta*100))

mapet

ypn<-(predictr(model4.6,xp))

yp<-round(ypn*(max-min)+min)

yp

mapep<-mean(abs((ypa-yp)/ypa*100))

mapep

model4.7<-trainr(yf,xf,learningrate = 0.2,hidden_dim = 3,numepochs = 150,batch_size = 10,network_type = "rnn")

par(mfrow=c(1,1))

plot(colMeans(model4.7$error),type = 'l',xlab = 'Epoch',ylab = 'Errors')

ytn<-(predictr(model4.7,xt))

yt<-round(ytn*(max-min)+min)

yt

mapet<-mean(abs((yta-yt)/yta*100))

mapet

ypn<-(predictr(model4.7,xp))

yp<-round(ypn*(max-min)+min)

yp

mapep<-mean(abs((ypa-yp)/ypa*100))

mapep

model4.8<-trainr(yf,xf,learningrate = 0.2,hidden_dim = 5,numepochs = 100,batch_size = 10,network_type = "rnn")

par(mfrow=c(1,1))

plot(colMeans(model4.8$error),type = 'l',xlab = 'Epoch',ylab = 'Errors')

ytn<-(predictr(model4.8,xt))

yt<-round(ytn*(max-min)+min)

yt

mapet<-mean(abs((yta-yt)/yta*100))

mapet

ypn<-(predictr(model4.8,xp))

yp<-round(ypn*(max-min)+min)

yp

mapep<-mean(abs((ypa-yp)/ypa*100))

mapep

model4.9<-trainr(yf,xf,learningrate = 0.2,hidden_dim = 10,numepochs = 80,batch_size = 10,network_type = "rnn")

par(mfrow=c(1,1))

plot(colMeans(model4.9$error),type = 'l',xlab = 'Epoch',ylab = 'Errors')

ytn<-(predictr(model4.9,xt))

yt<-round(ytn*(max-min)+min)

yt

mapet<-mean(abs((yta-yt)/yta*100))

mapet

ypn<-(predictr(model4.9,xp))

yp<-round(ypn*(max-min)+min)

yp

mapep<-mean(abs((ypa-yp)/ypa*100))

mapep

#5

x1<-xzrnn5$x1

x2<-xzrnn5$x2

x3<-xzrnn5$x3

x4<-xzrnn5$x4

x5<-xzrnn5$x5

x6<-xzrnn5$x6

x7<-xzrnn5$x7

x8<-xzrnn5$x8

x9<-xzrnn5$x9

x10<-xzrnn5$x10

x11<-xzrnn5$x11

x12<-xzrnn5$x12

y<-xzrnn5$y

max<-max(x1,x2,x3,x4,x5,x6,x7,x8,x9,x10,x11,x12,y)

min<-min(x1,x2,x3,x4,x5,x6,x7,x8,x9,x10,x11,x12,y)

x1n<-((x1-min)/(max-min))

x2n<-((x2-min)/(max-min))

x3n<-((x3-min)/(max-min))

x4n<-((x4-min)/(max-min))

x5n<-((x5-min)/(max-min))

x6n<-((x6-min)/(max-min))

x7n<-((x7-min)/(max-min))

x8n<-((x8-min)/(max-min))

x9n<-((x9-min)/(max-min))

x10n<-((x10-min)/(max-min))

x11n<-((x11-min)/(max-min))

x12n<-((x12-min)/(max-min))

yn<-((y-min)/(max-min))

xf1n<-x1n[1:132]

xf2n<-x2n[1:132]

xf3n<-x3n[1:132]

xf4n<-x4n[1:132]

xf5n<-x5n[1:132]

xf6n<-x6n[1:132]

xf7n<-x7n[1:132]

xf8n<-x8n[1:132]

xf9n<-x9n[1:132]

xf10n<-x10n[1:132]

xf11n<-x11n[1:132]

xf12n<-x12n[1:132]

yfn<-yn[1:132]

xt1n<-x1n[133:144]

xt2n<-x2n[133:144]

xt3n<-x3n[133:144]

xt4n<-x4n[133:144]

xt5n<-x5n[133:144]

xt6n<-x6n[133:144]

xt7n<-x7n[133:144]

xt8n<-x8n[133:144]

xt9n<-x9n[133:144]

xt10n<-x10n[133:144]

xt11n<-x11n[133:144]

xt12n<-x12n[133:144]

yta<-y[133:144]

xp1n<-x1n[145:156]

xp2n<-x2n[145:156]

xp3n<-x3n[145:156]

xp4n<-x4n[145:156]

xp5n<-x5n[145:156]

xp6n<-x6n[145:156]

xp7n<-x7n[145:156]

xp8n<-x8n[145:156]

xp9n<-x9n[145:156]

xp10n<-x10n[145:156]

xp11n<-x11n[145:156]

xp12n<-x12n[145:156]

ypa<-y[145:156]

xf<-array(c(xf1n,xf2n,xf3n,xf4n,xf5n,xf6n,xf7n,xf8n,xf9n,xf10n,xf11n,xf12n),dim = c(132,1,12))

yf<-array(yfn,dim = c(132,1,1))

xt<-array(c(xt1n,xt2n,xt3n,xt4n,xt5n,xt6n,xt7n,xt8n,xt9n,xt10n,xt11n,xt12n),dim = c(12,1,12))

xp<-array(c(xp1n,xp2n,xp3n,xp4n,xp5n,xp6n,xp7n,xp8n,xp9n,xp10n,xp11n,xp12n),dim = c(12,1,12))

model5.1<-trainr(yf,xf,learningrate = 0.05,hidden_dim = 3,numepochs = 800,batch_size = 10,network_type = "rnn")

par(mfrow=c(1,1))

plot(colMeans(model5.1$error),type = 'l',xlab = 'Epoch',ylab = 'Errors')

ytn<-(predictr(model5.1,xt))

yt<-round(ytn*(max-min)+min)

yt

mapet<-mean(abs((yta-yt)/yta*100))

mapet

ypn<-(predictr(model5.1,xp))

yp<-round(ypn*(max-min)+min)

yp

mapep<-mean(abs((ypa-yp)/ypa*100))

mapep

model5.2<-trainr(yf,xf,learningrate = 0.05,hidden_dim = 5,numepochs = 600,batch_size = 10,network_type = "rnn")

par(mfrow=c(1,1))

plot(colMeans(model5.2$error),type = 'l',xlab = 'Epoch',ylab = 'Errors')

ytn<-(predictr(model5.2,xt))

yt<-round(ytn*(max-min)+min)

yt

mapet<-mean(abs((yta-yt)/yta*100))

mapet

ypn<-(predictr(model5.2,xp))

yp<-round(ypn*(max-min)+min)

yp

mapep<-mean(abs((ypa-yp)/ypa*100))

mapep

model5.3<-trainr(yf,xf,learningrate = 0.05,hidden_dim = 10,numepochs = 400,batch_size = 10,network_type = "rnn")

par(mfrow=c(1,1))

plot(colMeans(model5.3$error),type = 'l',xlab = 'Epoch',ylab = 'Errors')

ytn<-(predictr(model5.3,xt))

yt<-round(ytn*(max-min)+min)

yt

mapet<-mean(abs((yta-yt)/yta*100))

mapet

ypn<-(predictr(model5.3,xp))

yp<-round(ypn*(max-min)+min)

yp

mapep<-mean(abs((ypa-yp)/ypa*100))

mapep

model5.4<-trainr(yf,xf,learningrate = 0.1,hidden_dim = 3,numepochs = 400,batch_size = 10,network_type = "rnn")

par(mfrow=c(1,1))

plot(colMeans(model5.4$error),type = 'l',xlab = 'Epoch',ylab = 'Errors')

ytn<-(predictr(model5.4,xt))

yt<-round(ytn*(max-min)+min)

yt

mapet<-mean(abs((yta-yt)/yta*100))

mapet

ypn<-(predictr(model5.4,xp))

yp<-round(ypn*(max-min)+min)

yp

mapep<-mean(abs((ypa-yp)/ypa*100))

mapep

model5.5<-trainr(yf,xf,learningrate = 0.1,hidden_dim = 5,numepochs = 300,batch_size = 10,network_type = "rnn")

par(mfrow=c(1,1))

plot(colMeans(model5.5$error),type = 'l',xlab = 'Epoch',ylab = 'Errors')

ytn<-(predictr(model5.5,xt))

yt<-round(ytn*(max-min)+min)

yt

mapet<-mean(abs((yta-yt)/yta*100))

mapet

ypn<-(predictr(model5.5,xp))

yp<-round(ypn*(max-min)+min)

yp

mapep<-mean(abs((ypa-yp)/ypa*100))

mapep

model5.6<-trainr(yf,xf,learningrate = 0.1,hidden_dim = 10,numepochs = 200,batch_size = 10,network_type = "rnn")

par(mfrow=c(1,1))

plot(colMeans(model5.6$error),type = 'l',xlab = 'Epoch',ylab = 'Errors')

ytn<-(predictr(model5.6,xt))

yt<-round(ytn*(max-min)+min)

yt

mapet<-mean(abs((yta-yt)/yta*100))

mapet

ypn<-(predictr(model5.6,xp))

yp<-round(ypn*(max-min)+min)

yp

mapep<-mean(abs((ypa-yp)/ypa*100))

mapep

model5.7<-trainr(yf,xf,learningrate = 0.2,hidden_dim = 3,numepochs = 150,batch_size = 10,network_type = "rnn")

par(mfrow=c(1,1))

plot(colMeans(model5.7$error),type = 'l',xlab = 'Epoch',ylab = 'Errors')

ytn<-(predictr(model5.7,xt))

yt<-round(ytn*(max-min)+min)

yt

mapet<-mean(abs((yta-yt)/yta*100))

mapet

ypn<-(predictr(model5.7,xp))

yp<-round(ypn*(max-min)+min)

yp

mapep<-mean(abs((ypa-yp)/ypa*100))

mapep

model5.8<-trainr(yf,xf,learningrate = 0.2,hidden_dim = 5,numepochs = 100,batch_size = 10,network_type = "rnn")

par(mfrow=c(1,1))

plot(colMeans(model5.8$error),type = 'l',xlab = 'Epoch',ylab = 'Errors')

ytn<-(predictr(model5.8,xt))

yt<-round(ytn*(max-min)+min)

yt

mapet<-mean(abs((yta-yt)/yta*100))

mapet

ypn<-(predictr(model5.8,xp))

yp<-round(ypn*(max-min)+min)

yp

mapep<-mean(abs((ypa-yp)/ypa*100))

mapep

model5.9<-trainr(yf,xf,learningrate = 0.2,hidden_dim = 10,numepochs = 80,batch_size = 10,network_type = "rnn")

par(mfrow=c(1,1))

plot(colMeans(model5.9$error),type = 'l',xlab = 'Epoch',ylab = 'Errors')

ytn<-(predictr(model5.9,xt))

yt<-round(ytn*(max-min)+min)

yt

mapet<-mean(abs((yta-yt)/yta*100))

mapet

ypn<-(predictr(model5.9,xp))

yp<-round(ypn*(max-min)+min)

yp

mapep<-mean(abs((ypa-yp)/ypa*100))

mapep

#6

x1<-xzrnn6$x1

x2<-xzrnn6$x2

x3<-xzrnn6$x3

x4<-xzrnn6$x4

x5<-xzrnn6$x5

x6<-xzrnn6$x6

x7<-xzrnn6$x7

x8<-xzrnn6$x8

x9<-xzrnn6$x9

x10<-xzrnn6$x10

x11<-xzrnn6$x11

x12<-xzrnn6$x12

x13<-xzrnn6$x13

y<-xzrnn6$y

max<-max(x1,x2,x3,x4,x5,x6,x7,x8,x9,x10,x11,x12,x13,y)

min<-min(x1,x2,x3,x4,x5,x6,x7,x8,x9,x10,x11,x12,x13,y)

x1n<-((x1-min)/(max-min))

x2n<-((x2-min)/(max-min))

x3n<-((x3-min)/(max-min))

x4n<-((x4-min)/(max-min))

x5n<-((x5-min)/(max-min))

x6n<-((x6-min)/(max-min))

x7n<-((x7-min)/(max-min))

x8n<-((x8-min)/(max-min))

x9n<-((x9-min)/(max-min))

x10n<-((x10-min)/(max-min))

x11n<-((x11-min)/(max-min))

x12n<-((x12-min)/(max-min))

x13n<-((x13-min)/(max-min))

yn<-((y-min)/(max-min))

xf1n<-x1n[1:132]

xf2n<-x2n[1:132]

xf3n<-x3n[1:132]

xf4n<-x4n[1:132]

xf5n<-x5n[1:132]

xf6n<-x6n[1:132]

xf7n<-x7n[1:132]

xf8n<-x8n[1:132]

xf9n<-x9n[1:132]

xf10n<-x10n[1:132]

xf11n<-x11n[1:132]

xf12n<-x12n[1:132]

xf13n<-x13n[1:132]

yfn<-yn[1:132]

xt1n<-x1n[133:144]

xt2n<-x2n[133:144]

xt3n<-x3n[133:144]

xt4n<-x4n[133:144]

xt5n<-x5n[133:144]

xt6n<-x6n[133:144]

xt7n<-x7n[133:144]

xt8n<-x8n[133:144]

xt9n<-x9n[133:144]

xt10n<-x10n[133:144]

xt11n<-x11n[133:144]

xt12n<-x12n[133:144]

xt13n<-x13n[133:144]

yta<-y[133:144]

xp1n<-x1n[145:156]

xp2n<-x2n[145:156]

xp3n<-x3n[145:156]

xp4n<-x4n[145:156]

xp5n<-x5n[145:156]

xp6n<-x6n[145:156]

xp7n<-x7n[145:156]

xp8n<-x8n[145:156]

xp9n<-x9n[145:156]

xp10n<-x10n[145:156]

xp11n<-x11n[145:156]

xp12n<-x12n[145:156]

xp13n<-x13n[145:156]

ypa<-y[145:156]

xf<-array(c(xf1n,xf2n,xf3n,xf4n,xf5n,xf6n,xf7n,xf8n,xf9n,xf10n,xf11n,xf12n,xf13n),dim = c(132,1,13))

yf<-array(yfn,dim = c(132,1,1))

xt<-array(c(xt1n,xt2n,xt3n,xt4n,xt5n,xt6n,xt7n,xt8n,xt9n,xt10n,xt11n,xt12n,xt13n),dim = c(12,1,13))

xp<-array(c(xp1n,xp2n,xp3n,xp4n,xp5n,xp6n,xp7n,xp8n,xp9n,xp10n,xp11n,xp12n,xp13n),dim = c(12,1,13))

model6.1<-trainr(yf,xf,learningrate = 0.05,hidden_dim = 3,numepochs = 1000,batch_size = 10,network_type = "rnn")

par(mfrow=c(1,1))

plot(colMeans(model6.1$error),type = 'l',xlab = 'Epoch',ylab = 'Errors')

ytn<-(predictr(model6.1,xt))

yt<-round(ytn*(max-min)+min)

yt

mapet<-mean(abs((yta-yt)/yta*100))

mapet

ypn<-(predictr(model6.1,xp))

yp<-round(ypn*(max-min)+min)

yp

mapep<-mean(abs((ypa-yp)/ypa*100))

mapep

model6.2<-trainr(yf,xf,learningrate = 0.05,hidden_dim = 5,numepochs = 800,batch_size = 10,network_type = "rnn")

par(mfrow=c(1,1))

plot(colMeans(model6.2$error),type = 'l',xlab = 'Epoch',ylab = 'Errors')

ytn<-(predictr(model6.2,xt))

yt<-round(ytn*(max-min)+min)

yt

mapet<-mean(abs((yta-yt)/yta*100))

mapet

ypn<-(predictr(model6.2,xp))

yp<-round(ypn*(max-min)+min)

yp

mapep<-mean(abs((ypa-yp)/ypa*100))

mapep

model6.3<-trainr(yf,xf,learningrate = 0.05,hidden_dim = 10,numepochs = 600,batch_size = 10,network_type = "rnn")

par(mfrow=c(1,1))

plot(colMeans(model6.3$error),type = 'l',xlab = 'Epoch',ylab = 'Errors')

ytn<-(predictr(model6.3,xt))

yt<-round(ytn*(max-min)+min)

yt

mapet<-mean(abs((yta-yt)/yta*100))

mapet

ypn<-(predictr(model6.3,xp))

yp<-round(ypn*(max-min)+min)

yp

mapep<-mean(abs((ypa-yp)/ypa*100))

mapep

model6.4<-trainr(yf,xf,learningrate = 0.1,hidden_dim = 3,numepochs = 500,batch_size = 10,network_type = "rnn")

par(mfrow=c(1,1))

plot(colMeans(model6.4$error),type = 'l',xlab = 'Epoch',ylab = 'Errors')

ytn<-(predictr(model6.4,xt))

yt<-round(ytn*(max-min)+min)

yt

mapet<-mean(abs((yta-yt)/yta*100))

mapet

ypn<-(predictr(model6.4,xp))

yp<-round(ypn*(max-min)+min)

yp

mapep<-mean(abs((ypa-yp)/ypa*100))

mapep

model6.5<-trainr(yf,xf,learningrate = 0.1,hidden_dim = 5,numepochs = 400,batch_size = 10,network_type = "rnn")

par(mfrow=c(1,1))

plot(colMeans(model6.5$error),type = 'l',xlab = 'Epoch',ylab = 'Errors')

ytn<-(predictr(model6.5,xt))

yt<-round(ytn*(max-min)+min)

yt

mapet<-mean(abs((yta-yt)/yta*100))

mapet

ypn<-(predictr(model6.5,xp))

yp<-round(ypn*(max-min)+min)

yp

mapep<-mean(abs((ypa-yp)/ypa*100))

mapep

model6.6<-trainr(yf,xf,learningrate = 0.1,hidden_dim = 10,numepochs = 250,batch_size = 10,network_type = "rnn")

par(mfrow=c(1,1))

plot(colMeans(model6.6$error),type = 'l',xlab = 'Epoch',ylab = 'Errors')

ytn<-(predictr(model6.6,xt))

yt<-round(ytn*(max-min)+min)

yt

mapet<-mean(abs((yta-yt)/yta*100))

mapet

ypn<-(predictr(model6.6,xp))

yp<-round(ypn*(max-min)+min)

yp

mapep<-mean(abs((ypa-yp)/ypa*100))

mapep

model6.7<-trainr(yf,xf,learningrate = 0.2,hidden_dim = 3,numepochs = 250,batch_size = 10,network_type = "rnn")

par(mfrow=c(1,1))

plot(colMeans(model6.7$error),type = 'l',xlab = 'Epoch',ylab = 'Errors')

ytn<-(predictr(model6.7,xt))

yt<-round(ytn*(max-min)+min)

yt

mapet<-mean(abs((yta-yt)/yta*100))

mapet

ypn<-(predictr(model6.7,xp))

yp<-round(ypn*(max-min)+min)

yp

mapep<-mean(abs((ypa-yp)/ypa*100))

mapep

model6.8<-trainr(yf,xf,learningrate = 0.2,hidden_dim = 5,numepochs = 200,batch_size = 10,network_type = "rnn")

par(mfrow=c(1,1))

plot(colMeans(model6.8$error),type = 'l',xlab = 'Epoch',ylab = 'Errors')

ytn<-(predictr(model6.8,xt))

yt<-round(ytn*(max-min)+min)

yt

mapet<-mean(abs((yta-yt)/yta*100))

mapet

ypn<-(predictr(model6.8,xp))

yp<-round(ypn*(max-min)+min)

yp

mapep<-mean(abs((ypa-yp)/ypa*100))

mapep

model6.9<-trainr(yf,xf,learningrate = 0.2,hidden_dim = 10,numepochs = 150,batch_size = 10,network_type = "rnn")

par(mfrow=c(1,1))

plot(colMeans(model6.9$error),type = 'l',xlab = 'Epoch',ylab = 'Errors')

ytn<-(predictr(model6.9,xt))

yt<-round(ytn*(max-min)+min)

yt

mapet<-mean(abs((yta-yt)/yta*100))

mapet

ypn<-(predictr(model6.9,xp))

yp<-round(ypn*(max-min)+min)

yp

mapep<-mean(abs((ypa-yp)/ypa*100))

mapep

#7

x1<-xzrnn7$x1

x2<-xzrnn7$x2

x3<-xzrnn7$x3

x4<-xzrnn7$x4

x5<-xzrnn7$x5

x6<-xzrnn7$x6

x7<-xzrnn7$x7

x8<-xzrnn7$x8

x9<-xzrnn7$x9

x10<-xzrnn7$x10

x11<-xzrnn7$x11

x12<-xzrnn7$x12

x13<-xzrnn7$x13

x14<-xzrnn7$x14

y<-xzrnn7$y

max<-max(x1,x2,x3,x4,x5,x6,x7,x8,x9,x10,x11,x12,x13,x14,y)

min<-min(x1,x2,x3,x4,x5,x6,x7,x8,x9,x10,x11,x12,x13,x14,y)

x1n<-((x1-min)/(max-min))

x2n<-((x2-min)/(max-min))

x3n<-((x3-min)/(max-min))

x4n<-((x4-min)/(max-min))

x5n<-((x5-min)/(max-min))

x6n<-((x6-min)/(max-min))

x7n<-((x7-min)/(max-min))

x8n<-((x8-min)/(max-min))

x9n<-((x9-min)/(max-min))

x10n<-((x10-min)/(max-min))

x11n<-((x11-min)/(max-min))

x12n<-((x12-min)/(max-min))

x13n<-((x13-min)/(max-min))

x14n<-((x14-min)/(max-min))

yn<-((y-min)/(max-min))

xf1n<-x1n[1:132]

xf2n<-x2n[1:132]

xf3n<-x3n[1:132]

xf4n<-x4n[1:132]

xf5n<-x5n[1:132]

xf6n<-x6n[1:132]

xf7n<-x7n[1:132]

xf8n<-x8n[1:132]

xf9n<-x9n[1:132]

xf10n<-x10n[1:132]

xf11n<-x11n[1:132]

xf12n<-x12n[1:132]

xf13n<-x13n[1:132]

xf14n<-x14n[1:132]

yfn<-yn[1:132]

xt1n<-x1n[133:144]

xt2n<-x2n[133:144]

xt3n<-x3n[133:144]

xt4n<-x4n[133:144]

xt5n<-x5n[133:144]

xt6n<-x6n[133:144]

xt7n<-x7n[133:144]

xt8n<-x8n[133:144]

xt9n<-x9n[133:144]

xt10n<-x10n[133:144]

xt11n<-x11n[133:144]

xt12n<-x12n[133:144]

xt13n<-x13n[133:144]

xt14n<-x14n[133:144]

yta<-y[133:144]

xp1n<-x1n[145:156]

xp2n<-x2n[145:156]

xp3n<-x3n[145:156]

xp4n<-x4n[145:156]

xp5n<-x5n[145:156]

xp6n<-x6n[145:156]

xp7n<-x7n[145:156]

xp8n<-x8n[145:156]

xp9n<-x9n[145:156]

xp10n<-x10n[145:156]

xp11n<-x11n[145:156]

xp12n<-x12n[145:156]

xp13n<-x13n[145:156]

xp14n<-x14n[145:156]

ypa<-y[145:156]

xf<-array(c(xf1n,xf2n,xf3n,xf4n,xf5n,xf6n,xf7n,xf8n,xf9n,xf10n,xf11n,xf12n,xf13n,xf14n),dim = c(132,1,14))

yf<-array(yfn,dim = c(132,1,1))

xt<-array(c(xt1n,xt2n,xt3n,xt4n,xt5n,xt6n,xt7n,xt8n,xt9n,xt10n,xt11n,xt12n,xt13n,xt14n),dim = c(12,1,14))

xp<-array(c(xp1n,xp2n,xp3n,xp4n,xp5n,xp6n,xp7n,xp8n,xp9n,xp10n,xp11n,xp12n,xp13n,xp14n),dim = c(12,1,14))

model7.1<-trainr(yf,xf,learningrate = 0.05,hidden_dim = 3,numepochs = 800,batch_size = 10,network_type = "rnn")

par(mfrow=c(1,1))

plot(colMeans(model7.1$error),type = 'l',xlab = 'Epoch',ylab = 'Errors')

ytn<-(predictr(model7.1,xt))

yt<-round(ytn*(max-min)+min)

yt

mapet<-mean(abs((yta-yt)/yta*100))

mapet

ypn<-(predictr(model7.1,xp))

yp<-round(ypn*(max-min)+min)

yp

mapep<-mean(abs((ypa-yp)/ypa*100))

mapep

model7.2<-trainr(yf,xf,learningrate = 0.05,hidden_dim = 5,numepochs = 700,batch_size = 10,network_type = "rnn")

par(mfrow=c(1,1))

plot(colMeans(model7.2$error),type = 'l',xlab = 'Epoch',ylab = 'Errors')

ytn<-(predictr(model7.2,xt))

yt<-round(ytn*(max-min)+min)

yt

mapet<-mean(abs((yta-yt)/yta*100))

mapet

ypn<-(predictr(model7.2,xp))

yp<-round(ypn*(max-min)+min)

yp

mapep<-mean(abs((ypa-yp)/ypa*100))

mapep

model7.3<-trainr(yf,xf,learningrate = 0.05,hidden_dim = 10,numepochs = 600,batch_size = 10,network_type = "rnn")

par(mfrow=c(1,1))

plot(colMeans(model7.3$error),type = 'l',xlab = 'Epoch',ylab = 'Errors')

ytn<-(predictr(model7.3,xt))

yt<-round(ytn*(max-min)+min)

yt

mapet<-mean(abs((yta-yt)/yta*100))

mapet

ypn<-(predictr(model7.3,xp))

yp<-round(ypn*(max-min)+min)

yp

mapep<-mean(abs((ypa-yp)/ypa*100))

mapep

model7.4<-trainr(yf,xf,learningrate = 0.1,hidden_dim = 3,numepochs = 500,batch_size = 10,network_type = "rnn")

par(mfrow=c(1,1))

plot(colMeans(model7.4$error),type = 'l',xlab = 'Epoch',ylab = 'Errors')

ytn<-(predictr(model7.4,xt))

yt<-round(ytn*(max-min)+min)

yt

mapet<-mean(abs((yta-yt)/yta*100))

mapet

ypn<-(predictr(model7.4,xp))

yp<-round(ypn*(max-min)+min)

yp

mapep<-mean(abs((ypa-yp)/ypa*100))

mapep

model7.5<-trainr(yf,xf,learningrate = 0.1,hidden_dim = 5,numepochs = 400,batch_size = 10,network_type = "rnn")

par(mfrow=c(1,1))

plot(colMeans(model7.5$error),type = 'l',xlab = 'Epoch',ylab = 'Errors')

ytn<-(predictr(model7.5,xt))

yt<-round(ytn*(max-min)+min)

yt

mapet<-mean(abs((yta-yt)/yta*100))

mapet

ypn<-(predictr(model7.5,xp))

yp<-round(ypn*(max-min)+min)

yp

mapep<-mean(abs((ypa-yp)/ypa*100))

mapep

model7.6<-trainr(yf,xf,learningrate = 0.1,hidden_dim = 10,numepochs = 250,batch_size = 10,network_type = "rnn")

par(mfrow=c(1,1))

plot(colMeans(model7.6$error),type = 'l',xlab = 'Epoch',ylab = 'Errors')

ytn<-(predictr(model7.6,xt))

yt<-round(ytn*(max-min)+min)

yt

mapet<-mean(abs((yta-yt)/yta*100))

mapet

ypn<-(predictr(model7.6,xp))

yp<-round(ypn*(max-min)+min)

yp

mapep<-mean(abs((ypa-yp)/ypa*100))

mapep

model7.7<-trainr(yf,xf,learningrate = 0.2,hidden_dim = 3,numepochs = 200,batch_size = 10,network_type = "rnn")

par(mfrow=c(1,1))

plot(colMeans(model7.7$error),type = 'l',xlab = 'Epoch',ylab = 'Errors')

ytn<-(predictr(model7.7,xt))

yt<-round(ytn*(max-min)+min)

yt

mapet<-mean(abs((yta-yt)/yta*100))

mapet

ypn<-(predictr(model7.7,xp))

yp<-round(ypn*(max-min)+min)

yp

mapep<-mean(abs((ypa-yp)/ypa*100))

mapep

model7.8<-trainr(yf,xf,learningrate = 0.2,hidden_dim = 5,numepochs = 150,batch_size = 10,network_type = "rnn")

par(mfrow=c(1,1))

plot(colMeans(model7.8$error),type = 'l',xlab = 'Epoch',ylab = 'Errors')

ytn<-(predictr(model7.8,xt))

yt<-round(ytn*(max-min)+min)

yt

mapet<-mean(abs((yta-yt)/yta*100))

mapet

ypn<-(predictr(model7.8,xp))

yp<-round(ypn*(max-min)+min)

yp

mapep<-mean(abs((ypa-yp)/ypa*100))

mapep

model7.9<-trainr(yf,xf,learningrate = 0.2,hidden_dim = 10,numepochs = 100,batch_size = 10,network_type = "rnn")

par(mfrow=c(1,1))

plot(colMeans(model7.9$error),type = 'l',xlab = 'Epoch',ylab = 'Errors')

ytn<-(predictr(model7.9,xt))

yt<-round(ytn*(max-min)+min)

yt

mapet<-mean(abs((yta-yt)/yta*100))

mapet

ypn<-(predictr(model7.9,xp))

yp<-round(ypn*(max-min)+min)

yp

mapep<-mean(abs((ypa-yp)/ypa*100))

mapep

#8

x1<-xzrnn8$x1

x2<-xzrnn8$x2

x3<-xzrnn8$x3

x4<-xzrnn8$x4

x5<-xzrnn8$x5

x6<-xzrnn8$x6

x7<-xzrnn8$x7

x8<-xzrnn8$x8

x9<-xzrnn8$x9

x10<-xzrnn8$x10

x11<-xzrnn8$x11

x12<-xzrnn8$x12

x13<-xzrnn8$x13

x14<-xzrnn8$x14

x15<-xzrnn8$x15

x16<-xzrnn8$x16

y<-xzrnn8$y

max<-max(x1,x2,x3,x4,x5,x6,x7,x8,x9,x10,x11,x12,x13,x14,x15,x16,y)

min<-min(x1,x2,x3,x4,x5,x6,x7,x8,x9,x10,x11,x12,x13,x14,x15,x16,y)

x1n<-((x1-min)/(max-min))

x2n<-((x2-min)/(max-min))

x3n<-((x3-min)/(max-min))

x4n<-((x4-min)/(max-min))

x5n<-((x5-min)/(max-min))

x6n<-((x6-min)/(max-min))

x7n<-((x7-min)/(max-min))

x8n<-((x8-min)/(max-min))

x9n<-((x9-min)/(max-min))

x10n<-((x10-min)/(max-min))

x11n<-((x11-min)/(max-min))

x12n<-((x12-min)/(max-min))

x13n<-((x13-min)/(max-min))

x14n<-((x14-min)/(max-min))

x15n<-((x15-min)/(max-min))

x16n<-((x16-min)/(max-min))

yn<-((y-min)/(max-min))

xf1n<-x1n[1:132]

xf2n<-x2n[1:132]

xf3n<-x3n[1:132]

xf4n<-x4n[1:132]

xf5n<-x5n[1:132]

xf6n<-x6n[1:132]

xf7n<-x7n[1:132]

xf8n<-x8n[1:132]

xf9n<-x9n[1:132]

xf10n<-x10n[1:132]

xf11n<-x11n[1:132]

xf12n<-x12n[1:132]

xf13n<-x13n[1:132]

xf14n<-x14n[1:132]

xf15n<-x15n[1:132]

xf16n<-x16n[1:132]

yfn<-yn[1:132]

xt1n<-x1n[133:144]

xt2n<-x2n[133:144]

xt3n<-x3n[133:144]

xt4n<-x4n[133:144]

xt5n<-x5n[133:144]

xt6n<-x6n[133:144]

xt7n<-x7n[133:144]

xt8n<-x8n[133:144]

xt9n<-x9n[133:144]

xt10n<-x10n[133:144]

xt11n<-x11n[133:144]

xt12n<-x12n[133:144]

xt13n<-x13n[133:144]

xt14n<-x14n[133:144]

xt15n<-x15n[133:144]

xt16n<-x16n[133:144]

yta<-y[133:144]

xp1n<-x1n[145:156]

xp2n<-x2n[145:156]

xp3n<-x3n[145:156]

xp4n<-x4n[145:156]

xp5n<-x5n[145:156]

xp6n<-x6n[145:156]

xp7n<-x7n[145:156]

xp8n<-x8n[145:156]

xp9n<-x9n[145:156]

xp10n<-x10n[145:156]

xp11n<-x11n[145:156]

xp12n<-x12n[145:156]

xp13n<-x13n[145:156]

xp14n<-x14n[145:156]

xp15n<-x15n[145:156]

xp16n<-x16n[145:156]

ypa<-y[145:156]

xf<-array(c(xf1n,xf2n,xf3n,xf4n,xf5n,xf6n,xf7n,xf8n,xf9n,xf10n,xf11n,xf12n,

xf13n,xf14n,xf15n,xf16n),dim = c(132,1,16))

yf<-array(yfn,dim = c(132,1,1))

xt<-array(c(xt1n,xt2n,xt3n,xt4n,xt5n,xt6n,xt7n,xt8n,xt9n,xt10n,xt11n,xt12n,

xt13n,xt14n,xt15n,xt16n),dim = c(12,1,16))

xp<-array(c(xp1n,xp2n,xp3n,xp4n,xp5n,xp6n,xp7n,xp8n,xp9n,xp10n,xp11n,xp12n,

xp13n,xp14n,xp15n,xp16n),dim = c(12,1,16))

model8.1<-trainr(yf,xf,learningrate = 0.05,hidden_dim = 3,numepochs = 800,batch_size = 10,network_type = "rnn")

par(mfrow=c(1,1))

plot(colMeans(model8.1$error),type = 'l',xlab = 'Epoch',ylab = 'Errors')

ytn<-(predictr(model8.1,xt))

yt<-round(ytn*(max-min)+min)

yt

mapet<-mean(abs((yta-yt)/yta*100))

mapet

ypn<-(predictr(model8.1,xp))

yp<-round(ypn*(max-min)+min)

yp

mapep<-mean(abs((ypa-yp)/ypa*100))

mapep

model8.2.1<-trainr(yf,xf,learningrate = 0.05,hidden_dim = 5,numepochs = 600,batch_size = 10,network_type = "rnn")

par(mfrow=c(1,1))

plot(colMeans(model8.2.1$error),type = 'l',xlab = 'Epoch',ylab = 'Errors',main = 'A1')

ytn1<-(predictr(model8.2.1,xt))

yt1<-round(ytn1*(max-min)+min)

yt1

mapet1<-mean(abs((yta-yt1)/yta*100))

mapet1

ypn1<-(predictr(model8.2.1,xp))

yp1<-round(ypn1*(max-min)+min)

yp1

mapep1<-mean(abs((ypa-yp1)/ypa*100))

mapep1

yyn1<-(predictr(model8.2.1,xf))

yy1<-round(yyn1*(max-min)+min)

yy1

model8.2.2<-trainr(yf,xf,learningrate = 0.05,hidden_dim = 5,numepochs = 600,batch_size = 10,network_type = "rnn")

par(mfrow=c(1,1))

plot(colMeans(model8.2.2$error),type = 'l',xlab = 'Epoch',ylab = 'Errors',main = 'A2')

ytn2<-(predictr(model8.2.2,xt))

yt2<-round(ytn2*(max-min)+min)

yt2

mapet2<-mean(abs((yta-yt2)/yta*100))

mapet2

ypn2<-(predictr(model8.2.2,xp))

yp2<-round(ypn2*(max-min)+min)

yp2

mapep2<-mean(abs((ypa-yp2)/ypa*100))

mapep2

model8.2.3<-trainr(yf,xf,learningrate = 0.05,hidden_dim = 5,numepochs = 600,batch_size = 10,network_type = "rnn")

par(mfrow=c(1,1))

plot(colMeans(model8.2.3$error),type = 'l',xlab = 'Epoch',ylab = 'Errors',main = 'A3')

ytn3<-(predictr(model8.2.3,xt))

yt3<-round(ytn3*(max-min)+min)

yt3

mapet3<-mean(abs((yta-yt3)/yta*100))

mapet3

ypn3<-(predictr(model8.2.3,xp))

yp3<-round(ypn3*(max-min)+min)

yp3

mapep3<-mean(abs((ypa-yp3)/ypa*100))

mapep3

par(mfrow=c(1,3))

plot(colMeans(model8.2.1$error),type = 'l',xlab = 'Epoch',ylab = 'Errors',main = 'A1')

plot(colMeans(model8.2.2$error),type = 'l',xlab = 'Epoch',ylab = 'Errors',main = 'A2')

plot(colMeans(model8.2.3$error),type = 'l',xlab = 'Epoch',ylab = 'Errors',main = 'A3')

model8.3<-trainr(yf,xf,learningrate = 0.05,hidden_dim = 10,numepochs = 500,batch_size = 10,network_type = "rnn")

par(mfrow=c(1,1))

plot(colMeans(model8.3$error),type = 'l',xlab = 'Epoch',ylab = 'Errors')

ytn<-(predictr(model8.3,xt))

yt<-round(ytn*(max-min)+min)

yt

mapet<-mean(abs((yta-yt)/yta*100))

mapet

ypn<-(predictr(model8.3,xp))

yp<-round(ypn*(max-min)+min)

yp

mapep<-mean(abs((ypa-yp)/ypa*100))

mapep

model8.4<-trainr(yf,xf,learningrate = 0.1,hidden_dim = 3,numepochs = 400,batch_size = 10,network_type = "rnn")

par(mfrow=c(1,1))

plot(colMeans(model8.4$error),type = 'l',xlab = 'Epoch',ylab = 'Errors')

ytn<-(predictr(model8.4,xt))

yt<-round(ytn*(max-min)+min)

yt

mapet<-mean(abs((yta-yt)/yta*100))

mapet

ypn<-(predictr(model8.4,xp))

yp<-round(ypn*(max-min)+min)

yp

mapep<-mean(abs((ypa-yp)/ypa*100))

mapep

model8.5<-trainr(yf,xf,learningrate = 0.1,hidden_dim = 5,numepochs = 300,batch_size = 10,network_type = "rnn")

par(mfrow=c(1,1))

plot(colMeans(model8.5$error),type = 'l',xlab = 'Epoch',ylab = 'Errors')

ytn<-(predictr(model8.5,xt))

yt<-round(ytn*(max-min)+min)

yt

mapet<-mean(abs((yta-yt)/yta*100))

mapet

ypn<-(predictr(model8.5,xp))

yp<-round(ypn*(max-min)+min)

yp

mapep<-mean(abs((ypa-yp)/ypa*100))

mapep

model8.6<-trainr(yf,xf,learningrate = 0.1,hidden_dim = 10,numepochs = 250,batch_size = 10,network_type = "rnn")

par(mfrow=c(1,1))

plot(colMeans(model8.6$error),type = 'l',xlab = 'Epoch',ylab = 'Errors')

ytn<-(predictr(model8.6,xt))

yt<-round(ytn*(max-min)+min)

yt

mapet<-mean(abs((yta-yt)/yta*100))

mapet

ypn<-(predictr(model8.6,xp))

yp<-round(ypn*(max-min)+min)

yp

mapep<-mean(abs((ypa-yp)/ypa*100))

mapep

model8.7<-trainr(yf,xf,learningrate = 0.2,hidden_dim = 3,numepochs = 200,batch_size = 10,network_type = "rnn")

par(mfrow=c(1,1))

plot(colMeans(model8.7$error),type = 'l',xlab = 'Epoch',ylab = 'Errors')

ytn<-(predictr(model8.7,xt))

yt<-round(ytn*(max-min)+min)

yt

mapet<-mean(abs((yta-yt)/yta*100))

mapet

ypn<-(predictr(model8.7,xp))

yp<-round(ypn*(max-min)+min)

yp

mapep<-mean(abs((ypa-yp)/ypa*100))

mapep

model8.8<-trainr(yf,xf,learningrate = 0.2,hidden_dim = 5,numepochs = 150,batch_size = 10,network_type = "rnn")

par(mfrow=c(1,1))

plot(colMeans(model8.8$error),type = 'l',xlab = 'Epoch',ylab = 'Errors')

ytn<-(predictr(model8.8,xt))

yt<-round(ytn*(max-min)+min)

yt

mapet<-mean(abs((yta-yt)/yta*100))

mapet

ypn<-(predictr(model8.8,xp))

yp<-round(ypn*(max-min)+min)

yp

mapep<-mean(abs((ypa-yp)/ypa*100))

mapep

model8.9<-trainr(yf,xf,learningrate = 0.2,hidden_dim = 10,numepochs = 100,batch_size = 10,network_type = "rnn")

par(mfrow=c(1,1))

plot(colMeans(model8.9$error),type = 'l',xlab = 'Epoch',ylab = 'Errors')

ytn<-(predictr(model8.9,xt))

yt<-round(ytn*(max-min)+min)

yt

mapet<-mean(abs((yta-yt)/yta*100))

mapet

ypn<-(predictr(model8.9,xp))

yp<-round(ypn*(max-min)+min)

yp

mapep<-mean(abs((ypa-yp)/ypa*100))

mapep

#9

x1<-xzrnn9$x1

x2<-xzrnn9$x2

x3<-xzrnn9$x3

x4<-xzrnn9$x4

x5<-xzrnn9$x5

x6<-xzrnn9$x6

x7<-xzrnn9$x7

x8<-xzrnn9$x8

x9<-xzrnn9$x9

x10<-xzrnn9$x10

x11<-xzrnn9$x11

x12<-xzrnn9$x12

x13<-xzrnn9$x13

x14<-xzrnn9$x14

x15<-xzrnn9$x15

x16<-xzrnn9$x16

x17<-xzrnn9$x17

x18<-xzrnn9$x18

x19<-xzrnn9$x19

y<-xzrnn9$y

max<-max(x1,x2,x3,x4,x5,x6,x7,x8,x9,x10,x11,x12,x13,x14,x15,x16,x17,x18,x19,y)

min<-min(x1,x2,x3,x4,x5,x6,x7,x8,x9,x10,x11,x12,x13,x14,x15,x16,x17,x18,x19,y)

x1n<-((x1-min)/(max-min))

x2n<-((x2-min)/(max-min))

x3n<-((x3-min)/(max-min))

x4n<-((x4-min)/(max-min))

x5n<-((x5-min)/(max-min))

x6n<-((x6-min)/(max-min))

x7n<-((x7-min)/(max-min))

x8n<-((x8-min)/(max-min))

x9n<-((x9-min)/(max-min))

x10n<-((x10-min)/(max-min))

x11n<-((x11-min)/(max-min))

x12n<-((x12-min)/(max-min))

x13n<-((x13-min)/(max-min))

x14n<-((x14-min)/(max-min))

x15n<-((x15-min)/(max-min))

x16n<-((x16-min)/(max-min))

x17n<-((x17-min)/(max-min))

x18n<-((x18-min)/(max-min))

x19n<-((x19-min)/(max-min))

yn<-((y-min)/(max-min))

xf1n<-x1n[1:132]

xf2n<-x2n[1:132]

xf3n<-x3n[1:132]

xf4n<-x4n[1:132]

xf5n<-x5n[1:132]

xf6n<-x6n[1:132]

xf7n<-x7n[1:132]

xf8n<-x8n[1:132]

xf9n<-x9n[1:132]

xf10n<-x10n[1:132]

xf11n<-x11n[1:132]

xf12n<-x12n[1:132]

xf13n<-x13n[1:132]

xf14n<-x14n[1:132]

xf15n<-x15n[1:132]

xf16n<-x16n[1:132]

xf17n<-x17n[1:132]

xf18n<-x18n[1:132]

xf19n<-x19n[1:132]

yfn<-yn[1:132]

xt1n<-x1n[133:144]

xt2n<-x2n[133:144]

xt3n<-x3n[133:144]

xt4n<-x4n[133:144]

xt5n<-x5n[133:144]

xt6n<-x6n[133:144]

xt7n<-x7n[133:144]

xt8n<-x8n[133:144]

xt9n<-x9n[133:144]

xt10n<-x10n[133:144]

xt11n<-x11n[133:144]

xt12n<-x12n[133:144]

xt13n<-x13n[133:144]

xt14n<-x14n[133:144]

xt15n<-x15n[133:144]

xt16n<-x16n[133:144]

xt17n<-x17n[133:144]

xt18n<-x18n[133:144]

xt19n<-x19n[133:144]

yta<-y[133:144]

xp1n<-x1n[145:156]

xp2n<-x2n[145:156]

xp3n<-x3n[145:156]

xp4n<-x4n[145:156]

xp5n<-x5n[145:156]

xp6n<-x6n[145:156]

xp7n<-x7n[145:156]

xp8n<-x8n[145:156]

xp9n<-x9n[145:156]

xp10n<-x10n[145:156]

xp11n<-x11n[145:156]

xp12n<-x12n[145:156]

xp13n<-x13n[145:156]

xp14n<-x14n[145:156]

xp15n<-x15n[145:156]

xp16n<-x16n[145:156]

xp17n<-x17n[145:156]

xp18n<-x18n[145:156]

xp19n<-x19n[145:156]

ypa<-y[145:156]

xf<-array(c(xf1n,xf2n,xf3n,xf4n,xf5n,xf6n,xf7n,xf8n,xf9n,xf10n,xf11n,xf12n,

xf13n,xf14n,xf15n,xf16n,xf17n,xf18n,xf19n),dim = c(132,1,19))

yf<-array(yfn,dim = c(132,1,1))

xt<-array(c(xt1n,xt2n,xt3n,xt4n,xt5n,xt6n,xt7n,xt8n,xt9n,xt10n,xt11n,xt12n,

xt13n,xt14n,xt15n,xt16n,xt17n,xt18n,xt19n),dim = c(12,1,19))

xp<-array(c(xp1n,xp2n,xp3n,xp4n,xp5n,xp6n,xp7n,xp8n,xp9n,xp10n,xp11n,xp12n,

xp13n,xp14n,xp15n,xp16n,xp17n,xp18n,xp19n),dim = c(12,1,19))

model9.1<-trainr(yf,xf,learningrate = 0.05,hidden_dim = 3,numepochs = 800,batch_size = 10,network_type = "rnn")

par(mfrow=c(1,1))

plot(colMeans(model9.1$error),type = 'l',xlab = 'Epoch',ylab = 'Errors')

ytn<-(predictr(model9.1,xt))

yt<-round(ytn*(max-min)+min)

yt

mapet<-mean(abs((yta-yt)/yta*100))

mapet

ypn<-(predictr(model9.1,xp))

yp<-round(ypn*(max-min)+min)

yp

mapep<-mean(abs((ypa-yp)/ypa*100))

mapep

model9.2<-trainr(yf,xf,learningrate = 0.05,hidden_dim = 5,numepochs = 700,batch_size = 10,network_type = "rnn")

par(mfrow=c(1,1))

plot(colMeans(model9.2$error),type = 'l',xlab = 'Epoch',ylab = 'Errors')

ytn<-(predictr(model9.2,xt))

yt<-round(ytn*(max-min)+min)

yt

mapet<-mean(abs((yta-yt)/yta*100))

mapet

ypn<-(predictr(model9.2,xp))

yp<-round(ypn*(max-min)+min)

yp

mapep<-mean(abs((ypa-yp)/ypa*100))

mapep

model9.3<-trainr(yf,xf,learningrate = 0.05,hidden_dim = 10,numepochs = 600,batch_size = 10,network_type = "rnn")

par(mfrow=c(1,1))

plot(colMeans(model9.3$error),type = 'l',xlab = 'Epoch',ylab = 'Errors')

ytn<-(predictr(model9.3,xt))

yt<-round(ytn*(max-min)+min)

yt

mapet<-mean(abs((yta-yt)/yta*100))

mapet

ypn<-(predictr(model9.3,xp))

yp<-round(ypn*(max-min)+min)

yp

mapep<-mean(abs((ypa-yp)/ypa*100))

mapep

model9.4<-trainr(yf,xf,learningrate = 0.1,hidden_dim = 3,numepochs = 400,batch_size = 10,network_type = "rnn")

par(mfrow=c(1,1))

plot(colMeans(model9.4$error),type = 'l',xlab = 'Epoch',ylab = 'Errors')

ytn<-(predictr(model9.4,xt))

yt<-round(ytn*(max-min)+min)

yt

mapet<-mean(abs((yta-yt)/yta*100))

mapet

ypn<-(predictr(model9.4,xp))

yp<-round(ypn*(max-min)+min)

yp

mapep<-mean(abs((ypa-yp)/ypa*100))

mapep

model9.5<-trainr(yf,xf,learningrate = 0.1,hidden_dim = 5,numepochs = 300,batch_size = 10,network_type = "rnn")

par(mfrow=c(1,1))

plot(colMeans(model9.5$error),type = 'l',xlab = 'Epoch',ylab = 'Errors')

ytn<-(predictr(model9.5,xt))

yt<-round(ytn*(max-min)+min)

yt

mapet<-mean(abs((yta-yt)/yta*100))

mapet

ypn<-(predictr(model9.5,xp))

yp<-round(ypn*(max-min)+min)

yp

mapep<-mean(abs((ypa-yp)/ypa*100))

mapep

model9.6<-trainr(yf,xf,learningrate = 0.1,hidden_dim = 10,numepochs = 250,batch_size = 10,network_type = "rnn")

par(mfrow=c(1,1))

plot(colMeans(model9.6$error),type = 'l',xlab = 'Epoch',ylab = 'Errors')

ytn<-(predictr(model9.6,xt))

yt<-round(ytn*(max-min)+min)

yt

mapet<-mean(abs((yta-yt)/yta*100))

mapet

ypn<-(predictr(model9.6,xp))

yp<-round(ypn*(max-min)+min)

yp

mapep<-mean(abs((ypa-yp)/ypa*100))

mapep

model9.7<-trainr(yf,xf,learningrate = 0.2,hidden_dim = 3,numepochs = 200,batch_size = 10,network_type = "rnn")

par(mfrow=c(1,1))

plot(colMeans(model9.7$error),type = 'l',xlab = 'Epoch',ylab = 'Errors')

ytn<-(predictr(model9.7,xt))

yt<-round(ytn*(max-min)+min)

yt

mapet<-mean(abs((yta-yt)/yta*100))

mapet

ypn<-(predictr(model9.7,xp))

yp<-round(ypn*(max-min)+min)

yp

mapep<-mean(abs((ypa-yp)/ypa*100))

mapep

model9.8<-trainr(yf,xf,learningrate = 0.2,hidden_dim = 5,numepochs = 150,batch_size = 10,network_type = "rnn")

par(mfrow=c(1,1))

plot(colMeans(model9.8$error),type = 'l',xlab = 'Epoch',ylab = 'Errors')

ytn<-(predictr(model9.8,xt))

yt<-round(ytn*(max-min)+min)

yt

mapet<-mean(abs((yta-yt)/yta*100))

mapet

ypn<-(predictr(model9.8,xp))

yp<-round(ypn*(max-min)+min)

yp

mapep<-mean(abs((ypa-yp)/ypa*100))

mapep

model9.9<-trainr(yf,xf,learningrate = 0.2,hidden_dim = 10,numepochs = 100,batch_size = 10,network_type = "rnn")

par(mfrow=c(1,1))

plot(colMeans(model9.9$error),type = 'l',xlab = 'Epoch',ylab = 'Errors')

ytn<-(predictr(model9.9,xt))

yt<-round(ytn*(max-min)+min)

yt

mapet<-mean(abs((yta-yt)/yta*100))

mapet

ypn<-(predictr(model9.9,xp))

yp<-round(ypn*(max-min)+min)

yp

mapep<-mean(abs((ypa-yp)/ypa*100))

mapep

##Nantong

#1

x1<-ntrnn1$x1

y<-ntrnn1$y

max<-max(x1,y)

min<-min(x1,y)

x1n<-((x1-min)/(max-min))

yn<-((y-min)/(max-min))

xf1n<-x1n[1:143]

yfn<-yn[1:143]

xt1n<-x1n[144:155]

yta<-y[144:155]

xp1n<-x1n[156:167]

ypa<-y[156:167]

xf<-array(xf1n,dim = c(143,1,1))

yf<-array(yfn,dim = c(143,1,1))

xt<-array(xt1n,dim = c(12,1,1))

xp<-array(xp1n,dim = c(12,1,1))

model1.1<-trainr(yf,xf,learningrate = 0.05,hidden_dim = 3,numepochs = 500,batch_size = 10,network_type = "rnn")

par(mfrow=c(1,1))

plot(colMeans(model1.1$error),type = 'l',xlab = 'Epoch',ylab = 'Errors')

ytn<-(predictr(model1.1,xt))

yt<-round(ytn*(max-min)+min)

yt

mapet<-mean(abs((yta-yt)/yta*100))

mapet

ypn<-(predictr(model1.1,xp))

yp<-round(ypn*(max-min)+min)

yp

mapep<-mean(abs((ypa-yp)/ypa*100))

mapep

model1.2<-trainr(yf,xf,learningrate = 0.05,hidden_dim = 5,numepochs = 400,batch_size = 10,network_type = "rnn")

par(mfrow=c(1,1))

plot(colMeans(model1.2$error),type = 'l',xlab = 'Epoch',ylab = 'Errors')

ytn<-(predictr(model1.2,xt))

yt<-round(ytn*(max-min)+min)

yt

mapet<-mean(abs((yta-yt)/yta*100))

mapet

ypn<-(predictr(model1.2,xp))

yp<-round(ypn*(max-min)+min)

yp

mapep<-mean(abs((ypa-yp)/ypa*100))

mapep

model1.3<-trainr(yf,xf,learningrate = 0.05,hidden_dim = 10,numepochs = 250,batch_size = 10,network_type = "rnn")

par(mfrow=c(1,1))

plot(colMeans(model1.3$error),type = 'l',xlab = 'Epoch',ylab = 'Errors')

ytn<-(predictr(model1.3,xt))

yt<-round(ytn*(max-min)+min)

yt

mapet<-mean(abs((yta-yt)/yta*100))

mapet

ypn<-(predictr(model1.3,xp))

yp<-round(ypn*(max-min)+min)

yp

mapep<-mean(abs((ypa-yp)/ypa*100))

mapep

model1.4<-trainr(yf,xf,learningrate = 0.1,hidden_dim = 3,numepochs = 200,batch_size = 10,network_type = "rnn")

par(mfrow=c(1,1))

plot(colMeans(model1.4$error),type = 'l',xlab = 'Epoch',ylab = 'Errors')

ytn<-(predictr(model1.4,xt))

yt<-round(ytn*(max-min)+min)

yt

mapet<-mean(abs((yta-yt)/yta*100))

mapet

ypn<-(predictr(model1.4,xp))

yp<-round(ypn*(max-min)+min)

yp

mapep<-mean(abs((ypa-yp)/ypa*100))

mapep

model1.5<-trainr(yf,xf,learningrate = 0.1,hidden_dim = 5,numepochs = 150,batch_size = 10,network_type = "rnn")

par(mfrow=c(1,1))

plot(colMeans(model1.5$error),type = 'l',xlab = 'Epoch',ylab = 'Errors')

ytn<-(predictr(model1.5,xt))

yt<-round(ytn*(max-min)+min)

yt

mapet<-mean(abs((yta-yt)/yta*100))

mapet

ypn<-(predictr(model1.5,xp))

yp<-round(ypn*(max-min)+min)

yp

mapep<-mean(abs((ypa-yp)/ypa*100))

mapep

model1.6<-trainr(yf,xf,learningrate = 0.1,hidden_dim = 10,numepochs = 100,batch_size = 10,network_type = "rnn")

par(mfrow=c(1,1))

plot(colMeans(model1.6$error),type = 'l',xlab = 'Epoch',ylab = 'Errors')

ytn<-(predictr(model1.6,xt))

yt<-round(ytn*(max-min)+min)

yt

mapet<-mean(abs((yta-yt)/yta*100))

mapet

ypn<-(predictr(model1.6,xp))

yp<-round(ypn*(max-min)+min)

yp

mapep<-mean(abs((ypa-yp)/ypa*100))

mapep

model1.7<-trainr(yf,xf,learningrate = 0.2,hidden_dim = 3,numepochs = 100,batch_size = 10,network_type = "rnn")

par(mfrow=c(1,1))

plot(colMeans(model1.7$error),type = 'l',xlab = 'Epoch',ylab = 'Errors')

ytn<-(predictr(model1.7,xt))

yt<-round(ytn*(max-min)+min)

yt

mapet<-mean(abs((yta-yt)/yta*100))

mapet

ypn<-(predictr(model1.7,xp))

yp<-round(ypn*(max-min)+min)

yp

mapep<-mean(abs((ypa-yp)/ypa*100))

mapep

model1.8<-trainr(yf,xf,learningrate = 0.2,hidden_dim = 5,numepochs = 80,batch_size = 10,network_type = "rnn")

par(mfrow=c(1,1))

plot(colMeans(model1.8$error),type = 'l',xlab = 'Epoch',ylab = 'Errors')

ytn<-(predictr(model1.8,xt))

yt<-round(ytn*(max-min)+min)

yt

mapet<-mean(abs((yta-yt)/yta*100))

mapet

ypn<-(predictr(model1.8,xp))

yp<-round(ypn*(max-min)+min)

yp

mapep<-mean(abs((ypa-yp)/ypa*100))

mapep

model1.9<-trainr(yf,xf,learningrate = 0.2,hidden_dim = 10,numepochs = 60,batch_size = 10,network_type = "rnn")

par(mfrow=c(1,1))

plot(colMeans(model1.9$error),type = 'l',xlab = 'Epoch',ylab = 'Errors')

ytn<-(predictr(model1.9,xt))

yt<-round(ytn*(max-min)+min)

yt

mapet<-mean(abs((yta-yt)/yta*100))

mapet

ypn<-(predictr(model1.9,xp))

yp<-round(ypn*(max-min)+min)

yp

mapep<-mean(abs((ypa-yp)/ypa*100))

mapep

#2

x1<-ntrnn2$x1

x2<-ntrnn2$x2

y<-ntrnn2$y

max<-max(x1,x2,y)

min<-min(x1,x2,y)

x1n<-((x1-min)/(max-min))

x2n<-((x2-min)/(max-min))

yn<-((y-min)/(max-min))

xf1n<-x1n[1:142]

xf2n<-x2n[1:142]

yfn<-yn[1:142]

xt1n<-x1n[143:154]

xt2n<-x2n[143:154]

yta<-y[143:154]

xp1n<-x1n[155:166]

xp2n<-x2n[155:166]

ypa<-y[155:166]

xf<-array(c(xf1n,xf2n),dim = c(142,1,2))

yf<-array(yfn,dim = c(142,1,1))

xt<-array(c(xt1n,xt2n),dim = c(12,1,2))

xp<-array(c(xp1n,xp2n),dim = c(12,1,2))

model2.1<-trainr(yf,xf,learningrate = 0.05,hidden_dim = 3,numepochs = 500,batch_size = 10,network_type = "rnn")

par(mfrow=c(1,1))

plot(colMeans(model2.1$error),type = 'l',xlab = 'Epoch',ylab = 'Errors')

ytn<-(predictr(model2.1,xt))

yt<-round(ytn*(max-min)+min)

yt

mapet<-mean(abs((yta-yt)/yta*100))

mapet

ypn<-(predictr(model2.1,xp))

yp<-round(ypn*(max-min)+min)

yp

mapep<-mean(abs((ypa-yp)/ypa*100))

mapep

model2.2<-trainr(yf,xf,learningrate = 0.05,hidden_dim = 5,numepochs = 400,batch_size = 10,network_type = "rnn")

par(mfrow=c(1,1))

plot(colMeans(model2.2$error),type = 'l',xlab = 'Epoch',ylab = 'Errors')

ytn<-(predictr(model2.2,xt))

yt<-round(ytn*(max-min)+min)

yt

mapet<-mean(abs((yta-yt)/yta*100))

mapet

ypn<-(predictr(model2.2,xp))

yp<-round(ypn*(max-min)+min)

yp

mapep<-mean(abs((ypa-yp)/ypa*100))

mapep

model2.3<-trainr(yf,xf,learningrate = 0.05,hidden_dim = 10,numepochs = 250,batch_size = 10,network_type = "rnn")

par(mfrow=c(1,1))

plot(colMeans(model2.3$error),type = 'l',xlab = 'Epoch',ylab = 'Errors')

ytn<-(predictr(model2.3,xt))

yt<-round(ytn*(max-min)+min)

yt

mapet<-mean(abs((yta-yt)/yta*100))

mapet

ypn<-(predictr(model2.3,xp))

yp<-round(ypn*(max-min)+min)

yp

mapep<-mean(abs((ypa-yp)/ypa*100))

mapep

model2.4<-trainr(yf,xf,learningrate = 0.1,hidden_dim = 3,numepochs = 200,batch_size = 10,network_type = "rnn")

par(mfrow=c(1,1))

plot(colMeans(model2.4$error),type = 'l',xlab = 'Epoch',ylab = 'Errors')

ytn<-(predictr(model2.4,xt))

yt<-round(ytn*(max-min)+min)

yt

mapet<-mean(abs((yta-yt)/yta*100))

mapet

ypn<-(predictr(model2.4,xp))

yp<-round(ypn*(max-min)+min)

yp

mapep<-mean(abs((ypa-yp)/ypa*100))

mapep

model2.5<-trainr(yf,xf,learningrate = 0.1,hidden_dim = 5,numepochs = 150,batch_size = 10,network_type = "rnn")

par(mfrow=c(1,1))

plot(colMeans(model2.5$error),type = 'l',xlab = 'Epoch',ylab = 'Errors')

ytn<-(predictr(model2.5,xt))

yt<-round(ytn*(max-min)+min)

yt

mapet<-mean(abs((yta-yt)/yta*100))

mapet

ypn<-(predictr(model2.5,xp))

yp<-round(ypn*(max-min)+min)

yp

mapep<-mean(abs((ypa-yp)/ypa*100))

mapep

model2.6<-trainr(yf,xf,learningrate = 0.1,hidden_dim = 10,numepochs = 100,batch_size = 10,network_type = "rnn")

par(mfrow=c(1,1))

plot(colMeans(model2.6$error),type = 'l',xlab = 'Epoch',ylab = 'Errors')

ytn<-(predictr(model2.6,xt))

yt<-round(ytn*(max-min)+min)

yt

mapet<-mean(abs((yta-yt)/yta*100))

mapet

ypn<-(predictr(model2.6,xp))

yp<-round(ypn*(max-min)+min)

yp

mapep<-mean(abs((ypa-yp)/ypa*100))

mapep

model2.7<-trainr(yf,xf,learningrate = 0.2,hidden_dim = 3,numepochs = 100,batch_size = 10,network_type = "rnn")

par(mfrow=c(1,1))

plot(colMeans(model2.7$error),type = 'l',xlab = 'Epoch',ylab = 'Errors')

ytn<-(predictr(model2.7,xt))

yt<-round(ytn*(max-min)+min)

yt

mapet<-mean(abs((yta-yt)/yta*100))

mapet

ypn<-(predictr(model2.7,xp))

yp<-round(ypn*(max-min)+min)

yp

mapep<-mean(abs((ypa-yp)/ypa*100))

mapep

model2.8<-trainr(yf,xf,learningrate = 0.2,hidden_dim = 5,numepochs = 80,batch_size = 10,network_type = "rnn")

par(mfrow=c(1,1))

plot(colMeans(model2.8$error),type = 'l',xlab = 'Epoch',ylab = 'Errors')

ytn<-(predictr(model2.8,xt))

yt<-round(ytn*(max-min)+min)

yt

mapet<-mean(abs((yta-yt)/yta*100))

mapet

ypn<-(predictr(model2.8,xp))

yp<-round(ypn*(max-min)+min)

yp

mapep<-mean(abs((ypa-yp)/ypa*100))

mapep

model2.9<-trainr(yf,xf,learningrate = 0.2,hidden_dim = 10,numepochs = 60,batch_size = 10,network_type = "rnn")

par(mfrow=c(1,1))

plot(colMeans(model2.9$error),type = 'l',xlab = 'Epoch',ylab = 'Errors')

ytn<-(predictr(model2.9,xt))

yt<-round(ytn*(max-min)+min)

yt

mapet<-mean(abs((yta-yt)/yta*100))

mapet

ypn<-(predictr(model2.9,xp))

yp<-round(ypn*(max-min)+min)

yp

mapep<-mean(abs((ypa-yp)/ypa*100))

mapep

#3

x1<-ntrnn3$x1

x2<-ntrnn3$x2

x3<-ntrnn3$x3

y<-ntrnn3$y

max<-max(x1,x2,x3,y)

min<-min(x1,x2,x3,y)

x1n<-((x1-min)/(max-min))

x2n<-((x2-min)/(max-min))

x3n<-((x3-min)/(max-min))

yn<-((y-min)/(max-min))

xf1n<-x1n[1:141]

xf2n<-x2n[1:141]

xf3n<-x3n[1:141]

yfn<-yn[1:141]

xt1n<-x1n[142:153]

xt2n<-x2n[142:153]

xt3n<-x3n[142:153]

yta<-y[142:153]

xp1n<-x1n[154:165]

xp2n<-x2n[154:165]

xp3n<-x3n[154:165]

ypa<-y[154:165]

xf<-array(c(xf1n,xf2n,xf3n),dim = c(141,1,3))

yf<-array(yfn,dim = c(141,1,1))

xt<-array(c(xt1n,xt2n,xt3n),dim = c(12,1,3))

xp<-array(c(xp1n,xp2n,xp3n),dim = c(12,1,3))

model3.1<-trainr(yf,xf,learningrate = 0.05,hidden_dim = 3,numepochs = 500,batch_size = 10,network_type = "rnn")

par(mfrow=c(1,1))

plot(colMeans(model3.1$error),type = 'l',xlab = 'Epoch',ylab = 'Errors')

ytn<-(predictr(model3.1,xt))

yt<-round(ytn*(max-min)+min)

yt

mapet<-mean(abs((yta-yt)/yta*100))

mapet

ypn<-(predictr(model3.1,xp))

yp<-round(ypn*(max-min)+min)

yp

mapep<-mean(abs((ypa-yp)/ypa*100))

mapep

model3.2<-trainr(yf,xf,learningrate = 0.05,hidden_dim = 5,numepochs = 400,batch_size = 10,network_type = "rnn")

par(mfrow=c(1,1))

plot(colMeans(model3.2$error),type = 'l',xlab = 'Epoch',ylab = 'Errors')

ytn<-(predictr(model3.2,xt))

yt<-round(ytn*(max-min)+min)

yt

mapet<-mean(abs((yta-yt)/yta*100))

mapet

ypn<-(predictr(model3.2,xp))

yp<-round(ypn*(max-min)+min)

yp

mapep<-mean(abs((ypa-yp)/ypa*100))

mapep

model3.3<-trainr(yf,xf,learningrate = 0.05,hidden_dim = 10,numepochs = 250,batch_size = 10,network_type = "rnn")

par(mfrow=c(1,1))

plot(colMeans(model3.3$error),type = 'l',xlab = 'Epoch',ylab = 'Errors')

ytn<-(predictr(model3.3,xt))

yt<-round(ytn*(max-min)+min)

yt

mapet<-mean(abs((yta-yt)/yta*100))

mapet

ypn<-(predictr(model3.3,xp))

yp<-round(ypn*(max-min)+min)

yp

mapep<-mean(abs((ypa-yp)/ypa*100))

mapep

model3.4<-trainr(yf,xf,learningrate = 0.1,hidden_dim = 3,numepochs = 250,batch_size = 10,network_type = "rnn")

par(mfrow=c(1,1))

plot(colMeans(model3.4$error),type = 'l',xlab = 'Epoch',ylab = 'Errors')

ytn<-(predictr(model3.4,xt))

yt<-round(ytn*(max-min)+min)

yt

mapet<-mean(abs((yta-yt)/yta*100))

mapet

ypn<-(predictr(model3.4,xp))

yp<-round(ypn*(max-min)+min)

yp

mapep<-mean(abs((ypa-yp)/ypa*100))

mapep

model3.5<-trainr(yf,xf,learningrate = 0.1,hidden_dim = 5,numepochs = 200,batch_size = 10,network_type = "rnn")

par(mfrow=c(1,1))

plot(colMeans(model3.5$error),type = 'l',xlab = 'Epoch',ylab = 'Errors')

ytn<-(predictr(model3.5,xt))

yt<-round(ytn*(max-min)+min)

yt

mapet<-mean(abs((yta-yt)/yta*100))

mapet

ypn<-(predictr(model3.5,xp))

yp<-round(ypn*(max-min)+min)

yp

mapep<-mean(abs((ypa-yp)/ypa*100))

mapep

model3.6<-trainr(yf,xf,learningrate = 0.1,hidden_dim = 10,numepochs = 150,batch_size = 10,network_type = "rnn")

par(mfrow=c(1,1))

plot(colMeans(model3.6$error),type = 'l',xlab = 'Epoch',ylab = 'Errors')

ytn<-(predictr(model3.6,xt))

yt<-round(ytn*(max-min)+min)

yt

mapet<-mean(abs((yta-yt)/yta*100))

mapet

ypn<-(predictr(model3.6,xp))

yp<-round(ypn*(max-min)+min)

yp

mapep<-mean(abs((ypa-yp)/ypa*100))

mapep

model3.7<-trainr(yf,xf,learningrate = 0.2,hidden_dim = 3,numepochs = 150,batch_size = 10,network_type = "rnn")

par(mfrow=c(1,1))

plot(colMeans(model3.7$error),type = 'l',xlab = 'Epoch',ylab = 'Errors')

ytn<-(predictr(model3.7,xt))

yt<-round(ytn*(max-min)+min)

yt

mapet<-mean(abs((yta-yt)/yta*100))

mapet

ypn<-(predictr(model3.7,xp))

yp<-round(ypn*(max-min)+min)

yp

mapep<-mean(abs((ypa-yp)/ypa*100))

mapep

model3.8<-trainr(yf,xf,learningrate = 0.2,hidden_dim = 5,numepochs = 100,batch_size = 10,network_type = "rnn")

par(mfrow=c(1,1))

plot(colMeans(model3.8$error),type = 'l',xlab = 'Epoch',ylab = 'Errors')

ytn<-(predictr(model3.8,xt))

yt<-round(ytn*(max-min)+min)

yt

mapet<-mean(abs((yta-yt)/yta*100))

mapet

ypn<-(predictr(model3.8,xp))

yp<-round(ypn*(max-min)+min)

yp

mapep<-mean(abs((ypa-yp)/ypa*100))

mapep

model3.9<-trainr(yf,xf,learningrate = 0.2,hidden_dim = 10,numepochs = 80,batch_size = 10,network_type = "rnn")

par(mfrow=c(1,1))

plot(colMeans(model3.9$error),type = 'l',xlab = 'Epoch',ylab = 'Errors')

ytn<-(predictr(model3.9,xt))

yt<-round(ytn*(max-min)+min)

yt

mapet<-mean(abs((yta-yt)/yta*100))

mapet

ypn<-(predictr(model3.9,xp))

yp<-round(ypn*(max-min)+min)

yp

mapep<-mean(abs((ypa-yp)/ypa*100))

mapep

#4

x1<-ntrnn4$x1

x2<-ntrnn4$x2

x3<-ntrnn4$x3

x4<-ntrnn4$x4

x5<-ntrnn4$x5

x6<-ntrnn4$x6

y<-ntrnn4$y

max<-max(x1,x2,x3,x4,x5,x6,y)

min<-min(x1,x2,x3,x4,x5,x6,y)

x1n<-((x1-min)/(max-min))

x2n<-((x2-min)/(max-min))

x3n<-((x3-min)/(max-min))

x4n<-((x4-min)/(max-min))

x5n<-((x5-min)/(max-min))

x6n<-((x6-min)/(max-min))

yn<-((y-min)/(max-min))

xf1n<-x1n[1:138]

xf2n<-x2n[1:138]

xf3n<-x3n[1:138]

xf4n<-x4n[1:138]

xf5n<-x5n[1:138]

xf6n<-x6n[1:138]

yfn<-yn[1:138]

xt1n<-x1n[139:150]

xt2n<-x2n[139:150]

xt3n<-x3n[139:150]

xt4n<-x4n[139:150]

xt5n<-x5n[139:150]

xt6n<-x6n[139:150]

yta<-y[139:150]

xp1n<-x1n[151:162]

xp2n<-x2n[151:162]

xp3n<-x3n[151:162]

xp4n<-x4n[151:162]

xp5n<-x5n[151:162]

xp6n<-x6n[151:162]

ypa<-y[151:162]

xf<-array(c(xf1n,xf2n,xf3n,xf4n,xf5n,xf6n),dim = c(138,1,6))

yf<-array(yfn,dim = c(138,1,1))

xt<-array(c(xt1n,xt2n,xt3n,xt4n,xt5n,xt6n),dim = c(12,1,6))

xp<-array(c(xp1n,xp2n,xp3n,xp4n,xp5n,xp6n),dim = c(12,1,6))

model4.1<-trainr(yf,xf,learningrate = 0.05,hidden_dim = 3,numepochs = 600,batch_size = 10,network_type = "rnn")

par(mfrow=c(1,1))

plot(colMeans(model4.1$error),type = 'l',xlab = 'Epoch',ylab = 'Errors')

ytn<-(predictr(model4.1,xt))

yt<-round(ytn*(max-min)+min)

yt

mapet<-mean(abs((yta-yt)/yta*100))

mapet

ypn<-(predictr(model4.1,xp))

yp<-round(ypn*(max-min)+min)

yp

mapep<-mean(abs((ypa-yp)/ypa*100))

mapep

model4.2<-trainr(yf,xf,learningrate = 0.05,hidden_dim = 5,numepochs = 500,batch_size = 10,network_type = "rnn")

par(mfrow=c(1,1))

plot(colMeans(model4.2$error),type = 'l',xlab = 'Epoch',ylab = 'Errors')

ytn<-(predictr(model4.2,xt))

yt<-round(ytn*(max-min)+min)

yt

mapet<-mean(abs((yta-yt)/yta*100))

mapet

ypn<-(predictr(model4.2,xp))

yp<-round(ypn*(max-min)+min)

yp

mapep<-mean(abs((ypa-yp)/ypa*100))

mapep

model4.3<-trainr(yf,xf,learningrate = 0.05,hidden_dim = 10,numepochs = 400,batch_size = 10,network_type = "rnn")

par(mfrow=c(1,1))

plot(colMeans(model4.3$error),type = 'l',xlab = 'Epoch',ylab = 'Errors')

ytn<-(predictr(model4.3,xt))

yt<-round(ytn*(max-min)+min)

yt

mapet<-mean(abs((yta-yt)/yta*100))

mapet

ypn<-(predictr(model4.3,xp))

yp<-round(ypn*(max-min)+min)

yp

mapep<-mean(abs((ypa-yp)/ypa*100))

mapep

model4.4<-trainr(yf,xf,learningrate = 0.1,hidden_dim = 3,numepochs = 250,batch_size = 10,network_type = "rnn")

par(mfrow=c(1,1))

plot(colMeans(model4.4$error),type = 'l',xlab = 'Epoch',ylab = 'Errors')

ytn<-(predictr(model4.4,xt))

yt<-round(ytn*(max-min)+min)

yt

mapet<-mean(abs((yta-yt)/yta*100))

mapet

ypn<-(predictr(model4.4,xp))

yp<-round(ypn*(max-min)+min)

yp

mapep<-mean(abs((ypa-yp)/ypa*100))

mapep

model4.5<-trainr(yf,xf,learningrate = 0.1,hidden_dim = 5,numepochs = 200,batch_size = 10,network_type = "rnn")

par(mfrow=c(1,1))

plot(colMeans(model4.5$error),type = 'l',xlab = 'Epoch',ylab = 'Errors')

ytn<-(predictr(model4.5,xt))

yt<-round(ytn*(max-min)+min)

yt

mapet<-mean(abs((yta-yt)/yta*100))

mapet

ypn<-(predictr(model4.5,xp))

yp<-round(ypn*(max-min)+min)

yp

mapep<-mean(abs((ypa-yp)/ypa*100))

mapep

model4.6<-trainr(yf,xf,learningrate = 0.1,hidden_dim = 10,numepochs = 150,batch_size = 10,network_type = "rnn")

par(mfrow=c(1,1))

plot(colMeans(model4.6$error),type = 'l',xlab = 'Epoch',ylab = 'Errors')

ytn<-(predictr(model4.6,xt))

yt<-round(ytn*(max-min)+min)

yt

mapet<-mean(abs((yta-yt)/yta*100))

mapet

ypn<-(predictr(model4.6,xp))

yp<-round(ypn*(max-min)+min)

yp

mapep<-mean(abs((ypa-yp)/ypa*100))

mapep

model4.7<-trainr(yf,xf,learningrate = 0.2,hidden_dim = 3,numepochs = 150,batch_size = 10,network_type = "rnn")

par(mfrow=c(1,1))

plot(colMeans(model4.7$error),type = 'l',xlab = 'Epoch',ylab = 'Errors')

ytn<-(predictr(model4.7,xt))

yt<-round(ytn*(max-min)+min)

yt

mapet<-mean(abs((yta-yt)/yta*100))

mapet

ypn<-(predictr(model4.7,xp))

yp<-round(ypn*(max-min)+min)

yp

mapep<-mean(abs((ypa-yp)/ypa*100))

mapep

model4.8<-trainr(yf,xf,learningrate = 0.2,hidden_dim = 5,numepochs = 100,batch_size = 10,network_type = "rnn")

par(mfrow=c(1,1))

plot(colMeans(model4.8$error),type = 'l',xlab = 'Epoch',ylab = 'Errors')

ytn<-(predictr(model4.8,xt))

yt<-round(ytn*(max-min)+min)

yt

mapet<-mean(abs((yta-yt)/yta*100))

mapet

ypn<-(predictr(model4.8,xp))

yp<-round(ypn*(max-min)+min)

yp

mapep<-mean(abs((ypa-yp)/ypa*100))

mapep

model4.9<-trainr(yf,xf,learningrate = 0.2,hidden_dim = 10,numepochs = 80,batch_size = 10,network_type = "rnn")

par(mfrow=c(1,1))

plot(colMeans(model4.9$error),type = 'l',xlab = 'Epoch',ylab = 'Errors')

ytn<-(predictr(model4.9,xt))

yt<-round(ytn*(max-min)+min)

yt

mapet<-mean(abs((yta-yt)/yta*100))

mapet

ypn<-(predictr(model4.9,xp))

yp<-round(ypn*(max-min)+min)

yp

mapep<-mean(abs((ypa-yp)/ypa*100))

mapep

#5

x1<-ntrnn5$x1

x2<-ntrnn5$x2

x3<-ntrnn5$x3

x4<-ntrnn5$x4

x5<-ntrnn5$x5

x6<-ntrnn5$x6

x7<-ntrnn5$x7

x8<-ntrnn5$x8

x9<-ntrnn5$x9

x10<-ntrnn5$x10

x11<-ntrnn5$x11

x12<-ntrnn5$x12

y<-ntrnn5$y

max<-max(x1,x2,x3,x4,x5,x6,x7,x8,x9,x10,x11,x12,y)

min<-min(x1,x2,x3,x4,x5,x6,x7,x8,x9,x10,x11,x12,y)

x1n<-((x1-min)/(max-min))

x2n<-((x2-min)/(max-min))

x3n<-((x3-min)/(max-min))

x4n<-((x4-min)/(max-min))

x5n<-((x5-min)/(max-min))

x6n<-((x6-min)/(max-min))

x7n<-((x7-min)/(max-min))

x8n<-((x8-min)/(max-min))

x9n<-((x9-min)/(max-min))

x10n<-((x10-min)/(max-min))

x11n<-((x11-min)/(max-min))

x12n<-((x12-min)/(max-min))

yn<-((y-min)/(max-min))

xf1n<-x1n[1:132]

xf2n<-x2n[1:132]

xf3n<-x3n[1:132]

xf4n<-x4n[1:132]

xf5n<-x5n[1:132]

xf6n<-x6n[1:132]

xf7n<-x7n[1:132]

xf8n<-x8n[1:132]

xf9n<-x9n[1:132]

xf10n<-x10n[1:132]

xf11n<-x11n[1:132]

xf12n<-x12n[1:132]

yfn<-yn[1:132]

xt1n<-x1n[133:144]

xt2n<-x2n[133:144]

xt3n<-x3n[133:144]

xt4n<-x4n[133:144]

xt5n<-x5n[133:144]

xt6n<-x6n[133:144]

xt7n<-x7n[133:144]

xt8n<-x8n[133:144]

xt9n<-x9n[133:144]

xt10n<-x10n[133:144]

xt11n<-x11n[133:144]

xt12n<-x12n[133:144]

yta<-y[133:144]

xp1n<-x1n[145:156]

xp2n<-x2n[145:156]

xp3n<-x3n[145:156]

xp4n<-x4n[145:156]

xp5n<-x5n[145:156]

xp6n<-x6n[145:156]

xp7n<-x7n[145:156]

xp8n<-x8n[145:156]

xp9n<-x9n[145:156]

xp10n<-x10n[145:156]

xp11n<-x11n[145:156]

xp12n<-x12n[145:156]

ypa<-y[145:156]

xf<-array(c(xf1n,xf2n,xf3n,xf4n,xf5n,xf6n,xf7n,xf8n,xf9n,xf10n,xf11n,xf12n),dim = c(132,1,12))

yf<-array(yfn,dim = c(132,1,1))

xt<-array(c(xt1n,xt2n,xt3n,xt4n,xt5n,xt6n,xt7n,xt8n,xt9n,xt10n,xt11n,xt12n),dim = c(12,1,12))

xp<-array(c(xp1n,xp2n,xp3n,xp4n,xp5n,xp6n,xp7n,xp8n,xp9n,xp10n,xp11n,xp12n),dim = c(12,1,12))

model5.1<-trainr(yf,xf,learningrate = 0.05,hidden_dim = 3,numepochs = 800,batch_size = 10,network_type = "rnn")

par(mfrow=c(1,1))

plot(colMeans(model5.1$error),type = 'l',xlab = 'Epoch',ylab = 'Errors')

ytn<-(predictr(model5.1,xt))

yt<-round(ytn*(max-min)+min)

yt

mapet<-mean(abs((yta-yt)/yta*100))

mapet

ypn<-(predictr(model5.1,xp))

yp<-round(ypn*(max-min)+min)

yp

mapep<-mean(abs((ypa-yp)/ypa*100))

mapep

model5.2<-trainr(yf,xf,learningrate = 0.05,hidden_dim = 5,numepochs = 600,batch_size = 10,network_type = "rnn")

par(mfrow=c(1,1))

plot(colMeans(model5.2$error),type = 'l',xlab = 'Epoch',ylab = 'Errors')

ytn<-(predictr(model5.2,xt))

yt<-round(ytn*(max-min)+min)

yt

mapet<-mean(abs((yta-yt)/yta*100))

mapet

ypn<-(predictr(model5.2,xp))

yp<-round(ypn*(max-min)+min)

yp

mapep<-mean(abs((ypa-yp)/ypa*100))

mapep

model5.3<-trainr(yf,xf,learningrate = 0.05,hidden_dim = 10,numepochs = 400,batch_size = 10,network_type = "rnn")

par(mfrow=c(1,1))

plot(colMeans(model5.3$error),type = 'l',xlab = 'Epoch',ylab = 'Errors')

ytn<-(predictr(model5.3,xt))

yt<-round(ytn*(max-min)+min)

yt

mapet<-mean(abs((yta-yt)/yta*100))

mapet

ypn<-(predictr(model5.3,xp))

yp<-round(ypn*(max-min)+min)

yp

mapep<-mean(abs((ypa-yp)/ypa*100))

mapep

model5.4<-trainr(yf,xf,learningrate = 0.1,hidden_dim = 3,numepochs = 400,batch_size = 10,network_type = "rnn")

par(mfrow=c(1,1))

plot(colMeans(model5.4$error),type = 'l',xlab = 'Epoch',ylab = 'Errors')

ytn<-(predictr(model5.4,xt))

yt<-round(ytn*(max-min)+min)

yt

mapet<-mean(abs((yta-yt)/yta*100))

mapet

ypn<-(predictr(model5.4,xp))

yp<-round(ypn*(max-min)+min)

yp

mapep<-mean(abs((ypa-yp)/ypa*100))

mapep

model5.5<-trainr(yf,xf,learningrate = 0.1,hidden_dim = 5,numepochs = 300,batch_size = 10,network_type = "rnn")

par(mfrow=c(1,1))

plot(colMeans(model5.5$error),type = 'l',xlab = 'Epoch',ylab = 'Errors')

ytn<-(predictr(model5.5,xt))

yt<-round(ytn*(max-min)+min)

yt

mapet<-mean(abs((yta-yt)/yta*100))

mapet

ypn<-(predictr(model5.5,xp))

yp<-round(ypn*(max-min)+min)

yp

mapep<-mean(abs((ypa-yp)/ypa*100))

mapep

model5.6<-trainr(yf,xf,learningrate = 0.1,hidden_dim = 10,numepochs = 200,batch_size = 10,network_type = "rnn")

par(mfrow=c(1,1))

plot(colMeans(model5.6$error),type = 'l',xlab = 'Epoch',ylab = 'Errors')

ytn<-(predictr(model5.6,xt))

yt<-round(ytn*(max-min)+min)

yt

mapet<-mean(abs((yta-yt)/yta*100))

mapet

ypn<-(predictr(model5.6,xp))

yp<-round(ypn*(max-min)+min)

yp

mapep<-mean(abs((ypa-yp)/ypa*100))

mapep

model5.7<-trainr(yf,xf,learningrate = 0.2,hidden_dim = 3,numepochs = 150,batch_size = 10,network_type = "rnn")

par(mfrow=c(1,1))

plot(colMeans(model5.7$error),type = 'l',xlab = 'Epoch',ylab = 'Errors')

ytn<-(predictr(model5.7,xt))

yt<-round(ytn*(max-min)+min)

yt

mapet<-mean(abs((yta-yt)/yta*100))

mapet

ypn<-(predictr(model5.7,xp))

yp<-round(ypn*(max-min)+min)

yp

mapep<-mean(abs((ypa-yp)/ypa*100))

mapep

model5.8<-trainr(yf,xf,learningrate = 0.2,hidden_dim = 5,numepochs = 100,batch_size = 10,network_type = "rnn")

par(mfrow=c(1,1))

plot(colMeans(model5.8$error),type = 'l',xlab = 'Epoch',ylab = 'Errors')

ytn<-(predictr(model5.8,xt))

yt<-round(ytn*(max-min)+min)

yt

mapet<-mean(abs((yta-yt)/yta*100))

mapet

ypn<-(predictr(model5.8,xp))

yp<-round(ypn*(max-min)+min)

yp

mapep<-mean(abs((ypa-yp)/ypa*100))

mapep

model5.9<-trainr(yf,xf,learningrate = 0.2,hidden_dim = 10,numepochs = 80,batch_size = 10,network_type = "rnn")

par(mfrow=c(1,1))

plot(colMeans(model5.9$error),type = 'l',xlab = 'Epoch',ylab = 'Errors')

ytn<-(predictr(model5.9,xt))

yt<-round(ytn*(max-min)+min)

yt

mapet<-mean(abs((yta-yt)/yta*100))

mapet

ypn<-(predictr(model5.9,xp))

yp<-round(ypn*(max-min)+min)

yp

mapep<-mean(abs((ypa-yp)/ypa*100))

mapep

#6

x1<-ntrnn6$x1

x2<-ntrnn6$x2

x3<-ntrnn6$x3

x4<-ntrnn6$x4

x5<-ntrnn6$x5

x6<-ntrnn6$x6

x7<-ntrnn6$x7

x8<-ntrnn6$x8

x9<-ntrnn6$x9

x10<-ntrnn6$x10

x11<-ntrnn6$x11

x12<-ntrnn6$x12

x13<-ntrnn6$x13

x14<-ntrnn6$x14

y<-ntrnn6$y

max<-max(x1,x2,x3,x4,x5,x6,x7,x8,x9,x10,x11,x12,x13,x14,y)

min<-min(x1,x2,x3,x4,x5,x6,x7,x8,x9,x10,x11,x12,x13,x14,y)

x1n<-((x1-min)/(max-min))

x2n<-((x2-min)/(max-min))

x3n<-((x3-min)/(max-min))

x4n<-((x4-min)/(max-min))

x5n<-((x5-min)/(max-min))

x6n<-((x6-min)/(max-min))

x7n<-((x7-min)/(max-min))

x8n<-((x8-min)/(max-min))

x9n<-((x9-min)/(max-min))

x10n<-((x10-min)/(max-min))

x11n<-((x11-min)/(max-min))

x12n<-((x12-min)/(max-min))

x13n<-((x13-min)/(max-min))

x14n<-((x14-min)/(max-min))

yn<-((y-min)/(max-min))

xf1n<-x1n[1:132]

xf2n<-x2n[1:132]

xf3n<-x3n[1:132]

xf4n<-x4n[1:132]

xf5n<-x5n[1:132]

xf6n<-x6n[1:132]

xf7n<-x7n[1:132]

xf8n<-x8n[1:132]

xf9n<-x9n[1:132]

xf10n<-x10n[1:132]

xf11n<-x11n[1:132]

xf12n<-x12n[1:132]

xf13n<-x13n[1:132]

xf14n<-x14n[1:132]

yfn<-yn[1:132]

xt1n<-x1n[133:144]

xt2n<-x2n[133:144]

xt3n<-x3n[133:144]

xt4n<-x4n[133:144]

xt5n<-x5n[133:144]

xt6n<-x6n[133:144]

xt7n<-x7n[133:144]

xt8n<-x8n[133:144]

xt9n<-x9n[133:144]

xt10n<-x10n[133:144]

xt11n<-x11n[133:144]

xt12n<-x12n[133:144]

xt13n<-x13n[133:144]

xt14n<-x14n[133:144]

yta<-y[133:144]

xp1n<-x1n[145:156]

xp2n<-x2n[145:156]

xp3n<-x3n[145:156]

xp4n<-x4n[145:156]

xp5n<-x5n[145:156]

xp6n<-x6n[145:156]

xp7n<-x7n[145:156]

xp8n<-x8n[145:156]

xp9n<-x9n[145:156]

xp10n<-x10n[145:156]

xp11n<-x11n[145:156]

xp12n<-x12n[145:156]

xp13n<-x13n[145:156]

xp14n<-x14n[145:156]

ypa<-y[145:156]

xf<-array(c(xf1n,xf2n,xf3n,xf4n,xf5n,xf6n,xf7n,xf8n,xf9n,xf10n,xf11n,xf12n,xf13n,xf14n),dim = c(132,1,14))

yf<-array(yfn,dim = c(132,1,1))

xt<-array(c(xt1n,xt2n,xt3n,xt4n,xt5n,xt6n,xt7n,xt8n,xt9n,xt10n,xt11n,xt12n,xt13n,xt14n),dim = c(12,1,14))

xp<-array(c(xp1n,xp2n,xp3n,xp4n,xp5n,xp6n,xp7n,xp8n,xp9n,xp10n,xp11n,xp12n,xp13n,xp14n),dim = c(12,1,14))

model6.1<-trainr(yf,xf,learningrate = 0.05,hidden_dim = 3,numepochs = 1200,batch_size = 10,network_type = "rnn")

par(mfrow=c(1,1))

plot(colMeans(model6.1$error),type = 'l',xlab = 'Epoch',ylab = 'Errors')

ytn<-(predictr(model6.1,xt))

yt<-round(ytn*(max-min)+min)

yt

mapet<-mean(abs((yta-yt)/yta*100))

mapet

ypn<-(predictr(model6.1,xp))

yp<-round(ypn*(max-min)+min)

yp

mapep<-mean(abs((ypa-yp)/ypa*100))

mapep

model6.2<-trainr(yf,xf,learningrate = 0.05,hidden_dim = 5,numepochs = 1000,batch_size = 10,network_type = "rnn")

par(mfrow=c(1,1))

plot(colMeans(model6.2$error),type = 'l',xlab = 'Epoch',ylab = 'Errors')

ytn<-(predictr(model6.2,xt))

yt<-round(ytn*(max-min)+min)

yt

mapet<-mean(abs((yta-yt)/yta*100))

mapet

ypn<-(predictr(model6.2,xp))

yp<-round(ypn*(max-min)+min)

yp

mapep<-mean(abs((ypa-yp)/ypa*100))

mapep

model6.3<-trainr(yf,xf,learningrate = 0.05,hidden_dim = 10,numepochs = 800,batch_size = 10,network_type = "rnn")

par(mfrow=c(1,1))

plot(colMeans(model6.3$error),type = 'l',xlab = 'Epoch',ylab = 'Errors')

ytn<-(predictr(model6.3,xt))

yt<-round(ytn*(max-min)+min)

yt

mapet<-mean(abs((yta-yt)/yta*100))

mapet

ypn<-(predictr(model6.3,xp))

yp<-round(ypn*(max-min)+min)

yp

mapep<-mean(abs((ypa-yp)/ypa*100))

mapep

model6.4<-trainr(yf,xf,learningrate = 0.1,hidden_dim = 3,numepochs = 600,batch_size = 10,network_type = "rnn")

par(mfrow=c(1,1))

plot(colMeans(model6.4$error),type = 'l',xlab = 'Epoch',ylab = 'Errors')

ytn<-(predictr(model6.4,xt))

yt<-round(ytn*(max-min)+min)

yt

mapet<-mean(abs((yta-yt)/yta*100))

mapet

ypn<-(predictr(model6.4,xp))

yp<-round(ypn*(max-min)+min)

yp

mapep<-mean(abs((ypa-yp)/ypa*100))

mapep

model6.5<-trainr(yf,xf,learningrate = 0.1,hidden_dim = 5,numepochs = 500,batch_size = 10,network_type = "rnn")

par(mfrow=c(1,1))

plot(colMeans(model6.5$error),type = 'l',xlab = 'Epoch',ylab = 'Errors')

ytn<-(predictr(model6.5,xt))

yt<-round(ytn*(max-min)+min)

yt

mapet<-mean(abs((yta-yt)/yta*100))

mapet

ypn<-(predictr(model6.5,xp))

yp<-round(ypn*(max-min)+min)

yp

mapep<-mean(abs((ypa-yp)/ypa*100))

mapep

model6.6<-trainr(yf,xf,learningrate = 0.1,hidden_dim = 10,numepochs = 400,batch_size = 10,network_type = "rnn")

par(mfrow=c(1,1))

plot(colMeans(model6.6$error),type = 'l',xlab = 'Epoch',ylab = 'Errors')

ytn<-(predictr(model6.6,xt))

yt<-round(ytn*(max-min)+min)

yt

mapet<-mean(abs((yta-yt)/yta*100))

mapet

ypn<-(predictr(model6.6,xp))

yp<-round(ypn*(max-min)+min)

yp

mapep<-mean(abs((ypa-yp)/ypa*100))

mapep

model6.7<-trainr(yf,xf,learningrate = 0.2,hidden_dim = 3,numepochs = 300,batch_size = 10,network_type = "rnn")

par(mfrow=c(1,1))

plot(colMeans(model6.7$error),type = 'l',xlab = 'Epoch',ylab = 'Errors')

ytn<-(predictr(model6.7,xt))

yt<-round(ytn*(max-min)+min)

yt

mapet<-mean(abs((yta-yt)/yta*100))

mapet

ypn<-(predictr(model6.7,xp))

yp<-round(ypn*(max-min)+min)

yp

mapep<-mean(abs((ypa-yp)/ypa*100))

mapep

model6.8<-trainr(yf,xf,learningrate = 0.2,hidden_dim = 5,numepochs = 200,batch_size = 10,network_type = "rnn")

par(mfrow=c(1,1))

plot(colMeans(model6.8$error),type = 'l',xlab = 'Epoch',ylab = 'Errors')

ytn<-(predictr(model6.8,xt))

yt<-round(ytn*(max-min)+min)

yt

mapet<-mean(abs((yta-yt)/yta*100))

mapet

ypn<-(predictr(model6.8,xp))

yp<-round(ypn*(max-min)+min)

yp

mapep<-mean(abs((ypa-yp)/ypa*100))

mapep

model6.9<-trainr(yf,xf,learningrate = 0.2,hidden_dim = 10,numepochs = 150,batch_size = 10,network_type = "rnn")

par(mfrow=c(1,1))

plot(colMeans(model6.9$error),type = 'l',xlab = 'Epoch',ylab = 'Errors')

ytn<-(predictr(model6.9,xt))

yt<-round(ytn*(max-min)+min)

yt

mapet<-mean(abs((yta-yt)/yta*100))

mapet

ypn<-(predictr(model6.9,xp))

yp<-round(ypn*(max-min)+min)

yp

mapep<-mean(abs((ypa-yp)/ypa*100))

mapep

#7

x1<-ntrnn7$x1

x2<-ntrnn7$x2

x3<-ntrnn7$x3

x4<-ntrnn7$x4

x5<-ntrnn7$x5

x6<-ntrnn7$x6

x7<-ntrnn7$x7

x8<-ntrnn7$x8

x9<-ntrnn7$x9

x10<-ntrnn7$x10

x11<-ntrnn7$x11

x12<-ntrnn7$x12

x13<-ntrnn7$x13

x14<-ntrnn7$x14

y<-ntrnn7$y

max<-max(x1,x2,x3,x4,x5,x6,x7,x8,x9,x10,x11,x12,x13,x14,y)

min<-min(x1,x2,x3,x4,x5,x6,x7,x8,x9,x10,x11,x12,x13,x14,y)

x1n<-((x1-min)/(max-min))

x2n<-((x2-min)/(max-min))

x3n<-((x3-min)/(max-min))

x4n<-((x4-min)/(max-min))

x5n<-((x5-min)/(max-min))

x6n<-((x6-min)/(max-min))

x7n<-((x7-min)/(max-min))

x8n<-((x8-min)/(max-min))

x9n<-((x9-min)/(max-min))

x10n<-((x10-min)/(max-min))

x11n<-((x11-min)/(max-min))

x12n<-((x12-min)/(max-min))

x13n<-((x13-min)/(max-min))

x14n<-((x14-min)/(max-min))

yn<-((y-min)/(max-min))

xf1n<-x1n[1:132]

xf2n<-x2n[1:132]

xf3n<-x3n[1:132]

xf4n<-x4n[1:132]

xf5n<-x5n[1:132]

xf6n<-x6n[1:132]

xf7n<-x7n[1:132]

xf8n<-x8n[1:132]

xf9n<-x9n[1:132]

xf10n<-x10n[1:132]

xf11n<-x11n[1:132]

xf12n<-x12n[1:132]

xf13n<-x13n[1:132]

xf14n<-x14n[1:132]

yfn<-yn[1:132]

xt1n<-x1n[133:144]

xt2n<-x2n[133:144]

xt3n<-x3n[133:144]

xt4n<-x4n[133:144]

xt5n<-x5n[133:144]

xt6n<-x6n[133:144]

xt7n<-x7n[133:144]

xt8n<-x8n[133:144]

xt9n<-x9n[133:144]

xt10n<-x10n[133:144]

xt11n<-x11n[133:144]

xt12n<-x12n[133:144]

xt13n<-x13n[133:144]

xt14n<-x14n[133:144]

yta<-y[133:144]

xp1n<-x1n[145:156]

xp2n<-x2n[145:156]

xp3n<-x3n[145:156]

xp4n<-x4n[145:156]

xp5n<-x5n[145:156]

xp6n<-x6n[145:156]

xp7n<-x7n[145:156]

xp8n<-x8n[145:156]

xp9n<-x9n[145:156]

xp10n<-x10n[145:156]

xp11n<-x11n[145:156]

xp12n<-x12n[145:156]

xp13n<-x13n[145:156]

xp14n<-x14n[145:156]

ypa<-y[145:156]

xf<-array(c(xf1n,xf2n,xf3n,xf4n,xf5n,xf6n,xf7n,xf8n,xf9n,xf10n,xf11n,xf12n,xf13n,xf14n),dim = c(132,1,14))

yf<-array(yfn,dim = c(132,1,1))

xt<-array(c(xt1n,xt2n,xt3n,xt4n,xt5n,xt6n,xt7n,xt8n,xt9n,xt10n,xt11n,xt12n,xt13n,xt14n),dim = c(12,1,14))

xp<-array(c(xp1n,xp2n,xp3n,xp4n,xp5n,xp6n,xp7n,xp8n,xp9n,xp10n,xp11n,xp12n,xp13n,xp14n),dim = c(12,1,14))

model7.1<-trainr(yf,xf,learningrate = 0.05,hidden_dim = 3,numepochs = 1200,batch_size = 10,network_type = "rnn")

par(mfrow=c(1,1))

plot(colMeans(model7.1$error),type = 'l',xlab = 'Epoch',ylab = 'Errors')

ytn<-(predictr(model7.1,xt))

yt<-round(ytn*(max-min)+min)

yt

mapet<-mean(abs((yta-yt)/yta*100))

mapet

ypn<-(predictr(model7.1,xp))

yp<-round(ypn*(max-min)+min)

yp

mapep<-mean(abs((ypa-yp)/ypa*100))

mapep

model7.2.1<-trainr(yf,xf,learningrate = 0.05,hidden_dim = 5,numepochs = 1000,batch_size = 10,network_type = "rnn")

par(mfrow=c(1,1))

plot(colMeans(model7.2.1$error),type = 'l',xlab = 'Epoch',ylab = 'Errors',main = 'B1')

ytn1<-(predictr(model7.2.1,xt))

yt1<-round(ytn1*(max-min)+min)

yt1

mapet1<-mean(abs((yta-yt1)/yta*100))

mapet1

ypn1<-(predictr(model7.2.1,xp))

yp1<-round(ypn1*(max-min)+min)

yp1

mapep1<-mean(abs((ypa-yp1)/ypa*100))

mapep1

model7.2.2<-trainr(yf,xf,learningrate = 0.05,hidden_dim = 5,numepochs = 1000,batch_size = 10,network_type = "rnn")

par(mfrow=c(1,1))

plot(colMeans(model7.2.2$error),type = 'l',xlab = 'Epoch',ylab = 'Errors',main = 'B2')

ytn2<-(predictr(model7.2.2,xt))

yt2<-round(ytn2*(max-min)+min)

yt2

mapet2<-mean(abs((yta-yt2)/yta*100))

mapet2

ypn2<-(predictr(model7.2.2,xp))

yp2<-round(ypn2*(max-min)+min)

yp2

mapep2<-mean(abs((ypa-yp2)/ypa*100))

mapep2

yyn2<-(predictr(model7.2.2,xf))

yy2<-round(yyn2*(max-min)+min)

yy2

model7.2.3<-trainr(yf,xf,learningrate = 0.05,hidden_dim = 5,numepochs = 1000,batch_size = 10,network_type = "rnn")

par(mfrow=c(1,1))

plot(colMeans(model7.2.3$error),type = 'l',xlab = 'Epoch',ylab = 'Errors',main = 'B3')

ytn3<-(predictr(model7.2.3,xt))

yt3<-round(ytn3*(max-min)+min)

yt3

mapet3<-mean(abs((yta-yt3)/yta*100))

mapet3

ypn3<-(predictr(model7.2.3,xp))

yp3<-round(ypn3*(max-min)+min)

yp3

mapep3<-mean(abs((ypa-yp3)/ypa*100))

mapep3

par(mfrow=c(1,3))

plot(colMeans(model7.2.1$error),type = 'l',xlab = 'Epoch',ylab = 'Errors',main = 'B1')

plot(colMeans(model7.2.2$error),type = 'l',xlab = 'Epoch',ylab = 'Errors',main = 'B2')

plot(colMeans(model7.2.3$error),type = 'l',xlab = 'Epoch',ylab = 'Errors',main = 'B3')

model7.3<-trainr(yf,xf,learningrate = 0.05,hidden_dim = 10,numepochs = 800,batch_size = 10,network_type = "rnn")

par(mfrow=c(1,1))

plot(colMeans(model7.3$error),type = 'l',xlab = 'Epoch',ylab = 'Errors')

ytn<-(predictr(model7.3,xt))

yt<-round(ytn*(max-min)+min)

yt

mapet<-mean(abs((yta-yt)/yta*100))

mapet

ypn<-(predictr(model7.3,xp))

yp<-round(ypn*(max-min)+min)

yp

mapep<-mean(abs((ypa-yp)/ypa*100))

mapep

model7.4<-trainr(yf,xf,learningrate = 0.1,hidden_dim = 3,numepochs = 600,batch_size = 10,network_type = "rnn")

par(mfrow=c(1,1))

plot(colMeans(model7.4$error),type = 'l',xlab = 'Epoch',ylab = 'Errors')

ytn<-(predictr(model7.4,xt))

yt<-round(ytn*(max-min)+min)

yt

mapet<-mean(abs((yta-yt)/yta*100))

mapet

ypn<-(predictr(model7.4,xp))

yp<-round(ypn*(max-min)+min)

yp

mapep<-mean(abs((ypa-yp)/ypa*100))

mapep

model7.5<-trainr(yf,xf,learningrate = 0.1,hidden_dim = 5,numepochs = 500,batch_size = 10,network_type = "rnn")

par(mfrow=c(1,1))

plot(colMeans(model7.5$error),type = 'l',xlab = 'Epoch',ylab = 'Errors')

ytn<-(predictr(model7.5,xt))

yt<-round(ytn*(max-min)+min)

yt

mapet<-mean(abs((yta-yt)/yta*100))

mapet

ypn<-(predictr(model7.5,xp))

yp<-round(ypn*(max-min)+min)

yp

mapep<-mean(abs((ypa-yp)/ypa*100))

mapep

model7.6<-trainr(yf,xf,learningrate = 0.1,hidden_dim = 10,numepochs = 400,batch_size = 10,network_type = "rnn")

par(mfrow=c(1,1))

plot(colMeans(model7.6$error),type = 'l',xlab = 'Epoch',ylab = 'Errors')

ytn<-(predictr(model7.6,xt))

yt<-round(ytn*(max-min)+min)

yt

mapet<-mean(abs((yta-yt)/yta*100))

mapet

ypn<-(predictr(model7.6,xp))

yp<-round(ypn*(max-min)+min)

yp

mapep<-mean(abs((ypa-yp)/ypa*100))

mapep

model7.7<-trainr(yf,xf,learningrate = 0.2,hidden_dim = 3,numepochs = 300,batch_size = 10,network_type = "rnn")

par(mfrow=c(1,1))

plot(colMeans(model7.7$error),type = 'l',xlab = 'Epoch',ylab = 'Errors')

ytn<-(predictr(model7.7,xt))

yt<-round(ytn*(max-min)+min)

yt

mapet<-mean(abs((yta-yt)/yta*100))

mapet

ypn<-(predictr(model7.7,xp))

yp<-round(ypn*(max-min)+min)

yp

mapep<-mean(abs((ypa-yp)/ypa*100))

mapep

model7.8<-trainr(yf,xf,learningrate = 0.2,hidden_dim = 5,numepochs = 200,batch_size = 10,network_type = "rnn")

par(mfrow=c(1,1))

plot(colMeans(model7.8$error),type = 'l',xlab = 'Epoch',ylab = 'Errors')

ytn<-(predictr(model7.8,xt))

yt<-round(ytn*(max-min)+min)

yt

mapet<-mean(abs((yta-yt)/yta*100))

mapet

ypn<-(predictr(model7.8,xp))

yp<-round(ypn*(max-min)+min)

yp

mapep<-mean(abs((ypa-yp)/ypa*100))

mapep

model7.9<-trainr(yf,xf,learningrate = 0.2,hidden_dim = 10,numepochs = 150,batch_size = 10,network_type = "rnn")

par(mfrow=c(1,1))

plot(colMeans(model7.9$error),type = 'l',xlab = 'Epoch',ylab = 'Errors')

ytn<-(predictr(model7.9,xt))

yt<-round(ytn*(max-min)+min)

yt

mapet<-mean(abs((yta-yt)/yta*100))

mapet

ypn<-(predictr(model7.9,xp))

yp<-round(ypn*(max-min)+min)

yp

mapep<-mean(abs((ypa-yp)/ypa*100))

mapep

#8

x1<-ntrnn8$x1

x2<-ntrnn8$x2

x3<-ntrnn8$x3

x4<-ntrnn8$x4

x5<-ntrnn8$x5

x6<-ntrnn8$x6

x7<-ntrnn8$x7

x8<-ntrnn8$x8

x9<-ntrnn8$x9

x10<-ntrnn8$x10

x11<-ntrnn8$x11

x12<-ntrnn8$x12

x13<-ntrnn8$x13

x14<-ntrnn8$x14

y<-ntrnn8$y

max<-max(x1,x2,x3,x4,x5,x6,x7,x8,x9,x10,x11,x12,x13,x14,y)

min<-min(x1,x2,x3,x4,x5,x6,x7,x8,x9,x10,x11,x12,x13,x14,y)

x1n<-((x1-min)/(max-min))

x2n<-((x2-min)/(max-min))

x3n<-((x3-min)/(max-min))

x4n<-((x4-min)/(max-min))

x5n<-((x5-min)/(max-min))

x6n<-((x6-min)/(max-min))

x7n<-((x7-min)/(max-min))

x8n<-((x8-min)/(max-min))

x9n<-((x9-min)/(max-min))

x10n<-((x10-min)/(max-min))

x11n<-((x11-min)/(max-min))

x12n<-((x12-min)/(max-min))

x13n<-((x13-min)/(max-min))

x14n<-((x14-min)/(max-min))

yn<-((y-min)/(max-min))

xf1n<-x1n[1:132]

xf2n<-x2n[1:132]

xf3n<-x3n[1:132]

xf4n<-x4n[1:132]

xf5n<-x5n[1:132]

xf6n<-x6n[1:132]

xf7n<-x7n[1:132]

xf8n<-x8n[1:132]

xf9n<-x9n[1:132]

xf10n<-x10n[1:132]

xf11n<-x11n[1:132]

xf12n<-x12n[1:132]

xf13n<-x13n[1:132]

xf14n<-x14n[1:132]

yfn<-yn[1:132]

xt1n<-x1n[133:144]

xt2n<-x2n[133:144]

xt3n<-x3n[133:144]

xt4n<-x4n[133:144]

xt5n<-x5n[133:144]

xt6n<-x6n[133:144]

xt7n<-x7n[133:144]

xt8n<-x8n[133:144]

xt9n<-x9n[133:144]

xt10n<-x10n[133:144]

xt11n<-x11n[133:144]

xt12n<-x12n[133:144]

xt13n<-x13n[133:144]

xt14n<-x14n[133:144]

yta<-y[133:144]

xp1n<-x1n[145:156]

xp2n<-x2n[145:156]

xp3n<-x3n[145:156]

xp4n<-x4n[145:156]

xp5n<-x5n[145:156]

xp6n<-x6n[145:156]

xp7n<-x7n[145:156]

xp8n<-x8n[145:156]

xp9n<-x9n[145:156]

xp10n<-x10n[145:156]

xp11n<-x11n[145:156]

xp12n<-x12n[145:156]

xp13n<-x13n[145:156]

xp14n<-x14n[145:156]

ypa<-y[145:156]

xf<-array(c(xf1n,xf2n,xf3n,xf4n,xf5n,xf6n,xf7n,xf8n,xf9n,xf10n,xf11n,xf12n,xf13n,xf14n),dim = c(132,1,14))

yf<-array(yfn,dim = c(132,1,1))

xt<-array(c(xt1n,xt2n,xt3n,xt4n,xt5n,xt6n,xt7n,xt8n,xt9n,xt10n,xt11n,xt12n,xt13n,xt14n),dim = c(12,1,14))

xp<-array(c(xp1n,xp2n,xp3n,xp4n,xp5n,xp6n,xp7n,xp8n,xp9n,xp10n,xp11n,xp12n,xp13n,xp14n),dim = c(12,1,14))

model8.1<-trainr(yf,xf,learningrate = 0.05,hidden_dim = 3,numepochs = 1200,batch_size = 10,network_type = "rnn")

par(mfrow=c(1,1))

plot(colMeans(model8.1$error),type = 'l',xlab = 'Epoch',ylab = 'Errors')

ytn<-(predictr(model8.1,xt))

yt<-round(ytn*(max-min)+min)

yt

mapet<-mean(abs((yta-yt)/yta*100))

mapet

ypn<-(predictr(model8.1,xp))

yp<-round(ypn*(max-min)+min)

yp

mapep<-mean(abs((ypa-yp)/ypa*100))

mapep

model8.2<-trainr(yf,xf,learningrate = 0.05,hidden_dim = 5,numepochs = 1000,batch_size = 10,network_type = "rnn")

par(mfrow=c(1,1))

plot(colMeans(model8.2$error),type = 'l',xlab = 'Epoch',ylab = 'Errors')

ytn<-(predictr(model8.2,xt))

yt<-round(ytn*(max-min)+min)

yt

mapet<-mean(abs((yta-yt)/yta*100))

mapet

ypn<-(predictr(model8.2,xp))

yp<-round(ypn*(max-min)+min)

yp

mapep<-mean(abs((ypa-yp)/ypa*100))

mapep

model8.3<-trainr(yf,xf,learningrate = 0.05,hidden_dim = 10,numepochs = 800,batch_size = 10,network_type = "rnn")

par(mfrow=c(1,1))

plot(colMeans(model8.3$error),type = 'l',xlab = 'Epoch',ylab = 'Errors')

ytn<-(predictr(model8.3,xt))

yt<-round(ytn*(max-min)+min)

yt

mapet<-mean(abs((yta-yt)/yta*100))

mapet

ypn<-(predictr(model8.3,xp))

yp<-round(ypn*(max-min)+min)

yp

mapep<-mean(abs((ypa-yp)/ypa*100))

mapep

model8.4<-trainr(yf,xf,learningrate = 0.1,hidden_dim = 3,numepochs = 600,batch_size = 10,network_type = "rnn")

par(mfrow=c(1,1))

plot(colMeans(model8.4$error),type = 'l',xlab = 'Epoch',ylab = 'Errors')

ytn<-(predictr(model8.4,xt))

yt<-round(ytn*(max-min)+min)

yt

mapet<-mean(abs((yta-yt)/yta*100))

mapet

ypn<-(predictr(model8.4,xp))

yp<-round(ypn*(max-min)+min)

yp

mapep<-mean(abs((ypa-yp)/ypa*100))

mapep

model8.5<-trainr(yf,xf,learningrate = 0.1,hidden_dim = 5,numepochs = 400,batch_size = 10,network_type = "rnn")

par(mfrow=c(1,1))

plot(colMeans(model8.5$error),type = 'l',xlab = 'Epoch',ylab = 'Errors')

ytn<-(predictr(model8.5,xt))

yt<-round(ytn*(max-min)+min)

yt

mapet<-mean(abs((yta-yt)/yta*100))

mapet

ypn<-(predictr(model8.5,xp))

yp<-round(ypn*(max-min)+min)

yp

mapep<-mean(abs((ypa-yp)/ypa*100))

mapep

model8.6<-trainr(yf,xf,learningrate = 0.1,hidden_dim = 10,numepochs = 300,batch_size = 10,network_type = "rnn")

par(mfrow=c(1,1))

plot(colMeans(model8.6$error),type = 'l',xlab = 'Epoch',ylab = 'Errors')

ytn<-(predictr(model8.6,xt))

yt<-round(ytn*(max-min)+min)

yt

mapet<-mean(abs((yta-yt)/yta*100))

mapet

ypn<-(predictr(model8.6,xp))

yp<-round(ypn*(max-min)+min)

yp

mapep<-mean(abs((ypa-yp)/ypa*100))

mapep

model8.7<-trainr(yf,xf,learningrate = 0.2,hidden_dim = 3,numepochs = 250,batch_size = 10,network_type = "rnn")

par(mfrow=c(1,1))

plot(colMeans(model8.7$error),type = 'l',xlab = 'Epoch',ylab = 'Errors')

ytn<-(predictr(model8.7,xt))

yt<-round(ytn*(max-min)+min)

yt

mapet<-mean(abs((yta-yt)/yta*100))

mapet

ypn<-(predictr(model8.7,xp))

yp<-round(ypn*(max-min)+min)

yp

mapep<-mean(abs((ypa-yp)/ypa*100))

mapep

model8.8<-trainr(yf,xf,learningrate = 0.2,hidden_dim = 5,numepochs = 200,batch_size = 10,network_type = "rnn")

par(mfrow=c(1,1))

plot(colMeans(model8.8$error),type = 'l',xlab = 'Epoch',ylab = 'Errors')

ytn<-(predictr(model8.8,xt))

yt<-round(ytn*(max-min)+min)

yt

mapet<-mean(abs((yta-yt)/yta*100))

mapet

ypn<-(predictr(model8.8,xp))

yp<-round(ypn*(max-min)+min)

yp

mapep<-mean(abs((ypa-yp)/ypa*100))

mapep

model8.9<-trainr(yf,xf,learningrate = 0.2,hidden_dim = 10,numepochs = 150,batch_size = 10,network_type = "rnn")

par(mfrow=c(1,1))

plot(colMeans(model8.9$error),type = 'l',xlab = 'Epoch',ylab = 'Errors')

ytn<-(predictr(model8.9,xt))

yt<-round(ytn*(max-min)+min)

yt

mapet<-mean(abs((yta-yt)/yta*100))

mapet

ypn<-(predictr(model8.9,xp))

yp<-round(ypn*(max-min)+min)

yp

mapep<-mean(abs((ypa-yp)/ypa*100))

mapep

#9

x1<-ntrnn9$x1

x2<-ntrnn9$x2

x3<-ntrnn9$x3

x4<-ntrnn9$x4

x5<-ntrnn9$x5

x6<-ntrnn9$x6

x7<-ntrnn9$x7

x8<-ntrnn9$x8

x9<-ntrnn9$x9

x10<-ntrnn9$x10

x11<-ntrnn9$x11

x12<-ntrnn9$x12

x13<-ntrnn9$x13

x14<-ntrnn9$x14

x15<-ntrnn9$x15

x16<-ntrnn9$x16

x17<-ntrnn9$x17

x18<-ntrnn9$x18

y<-ntrnn9$y

max<-max(x1,x2,x3,x4,x5,x6,x7,x8,x9,x10,x11,x12,x13,x14,x15,x16,x17,x18,y)

min<-min(x1,x2,x3,x4,x5,x6,x7,x8,x9,x10,x11,x12,x13,x14,x15,x16,x17,x18,y)

x1n<-((x1-min)/(max-min))

x2n<-((x2-min)/(max-min))

x3n<-((x3-min)/(max-min))

x4n<-((x4-min)/(max-min))

x5n<-((x5-min)/(max-min))

x6n<-((x6-min)/(max-min))

x7n<-((x7-min)/(max-min))

x8n<-((x8-min)/(max-min))

x9n<-((x9-min)/(max-min))

x10n<-((x10-min)/(max-min))

x11n<-((x11-min)/(max-min))

x12n<-((x12-min)/(max-min))

x13n<-((x13-min)/(max-min))

x14n<-((x14-min)/(max-min))

x15n<-((x15-min)/(max-min))

x16n<-((x16-min)/(max-min))

x17n<-((x17-min)/(max-min))

x18n<-((x18-min)/(max-min))

yn<-((y-min)/(max-min))

xf1n<-x1n[1:132]

xf2n<-x2n[1:132]

xf3n<-x3n[1:132]

xf4n<-x4n[1:132]

xf5n<-x5n[1:132]

xf6n<-x6n[1:132]

xf7n<-x7n[1:132]

xf8n<-x8n[1:132]

xf9n<-x9n[1:132]

xf10n<-x10n[1:132]

xf11n<-x11n[1:132]

xf12n<-x12n[1:132]

xf13n<-x13n[1:132]

xf14n<-x14n[1:132]

xf15n<-x15n[1:132]

xf16n<-x16n[1:132]

xf17n<-x17n[1:132]

xf18n<-x18n[1:132]

yfn<-yn[1:132]

xt1n<-x1n[133:144]

xt2n<-x2n[133:144]

xt3n<-x3n[133:144]

xt4n<-x4n[133:144]

xt5n<-x5n[133:144]

xt6n<-x6n[133:144]

xt7n<-x7n[133:144]

xt8n<-x8n[133:144]

xt9n<-x9n[133:144]

xt10n<-x10n[133:144]

xt11n<-x11n[133:144]

xt12n<-x12n[133:144]

xt13n<-x13n[133:144]

xt14n<-x14n[133:144]

xt15n<-x15n[133:144]

xt16n<-x16n[133:144]

xt17n<-x17n[133:144]

xt18n<-x18n[133:144]

yta<-y[133:144]

xp1n<-x1n[145:156]

xp2n<-x2n[145:156]

xp3n<-x3n[145:156]

xp4n<-x4n[145:156]

xp5n<-x5n[145:156]

xp6n<-x6n[145:156]

xp7n<-x7n[145:156]

xp8n<-x8n[145:156]

xp9n<-x9n[145:156]

xp10n<-x10n[145:156]

xp11n<-x11n[145:156]

xp12n<-x12n[145:156]

xp13n<-x13n[145:156]

xp14n<-x14n[145:156]

xp15n<-x15n[145:156]

xp16n<-x16n[145:156]

xp17n<-x17n[145:156]

xp18n<-x18n[145:156]

ypa<-y[145:156]

xf<-array(c(xf1n,xf2n,xf3n,xf4n,xf5n,xf6n,xf7n,xf8n,xf9n,xf10n,xf11n,xf12n,

xf13n,xf14n,xf15n,xf16n,xf17n,xf18n),dim = c(132,1,18))

yf<-array(yfn,dim = c(132,1,1))

xt<-array(c(xt1n,xt2n,xt3n,xt4n,xt5n,xt6n,xt7n,xt8n,xt9n,xt10n,xt11n,xt12n,

xt13n,xt14n,xt15n,xt16n,xt17n,xt18n),dim = c(12,1,18))

xp<-array(c(xp1n,xp2n,xp3n,xp4n,xp5n,xp6n,xp7n,xp8n,xp9n,xp10n,xp11n,xp12n,

xp13n,xp14n,xp15n,xp16n,xp17n,xp18n),dim = c(12,1,18))

model9.1<-trainr(yf,xf,learningrate = 0.05,hidden_dim = 3,numepochs = 1000,batch_size = 10,network_type = "rnn")

par(mfrow=c(1,1))

plot(colMeans(model9.1$error),type = 'l',xlab = 'Epoch',ylab = 'Errors')

ytn<-(predictr(model9.1,xt))

yt<-round(ytn*(max-min)+min)

yt

mapet<-mean(abs((yta-yt)/yta*100))

mapet

ypn<-(predictr(model9.1,xp))

yp<-round(ypn*(max-min)+min)

yp

mapep<-mean(abs((ypa-yp)/ypa*100))

mapep

model9.2<-trainr(yf,xf,learningrate = 0.05,hidden_dim = 5,numepochs = 800,batch_size = 10,network_type = "rnn")

par(mfrow=c(1,1))

plot(colMeans(model9.2$error),type = 'l',xlab = 'Epoch',ylab = 'Errors')

ytn<-(predictr(model9.2,xt))

yt<-round(ytn*(max-min)+min)

yt

mapet<-mean(abs((yta-yt)/yta*100))

mapet

ypn<-(predictr(model9.2,xp))

yp<-round(ypn*(max-min)+min)

yp

mapep<-mean(abs((ypa-yp)/ypa*100))

mapep

model9.3<-trainr(yf,xf,learningrate = 0.05,hidden_dim = 10,numepochs = 600,batch_size = 10,network_type = "rnn")

par(mfrow=c(1,1))

plot(colMeans(model9.3$error),type = 'l',xlab = 'Epoch',ylab = 'Errors')

ytn<-(predictr(model9.3,xt))

yt<-round(ytn*(max-min)+min)

yt

mapet<-mean(abs((yta-yt)/yta*100))

mapet

ypn<-(predictr(model9.3,xp))

yp<-round(ypn*(max-min)+min)

yp

mapep<-mean(abs((ypa-yp)/ypa*100))

mapep

model9.4<-trainr(yf,xf,learningrate = 0.1,hidden_dim = 3,numepochs = 600,batch_size = 10,network_type = "rnn")

par(mfrow=c(1,1))

plot(colMeans(model9.4$error),type = 'l',xlab = 'Epoch',ylab = 'Errors')

ytn<-(predictr(model9.4,xt))

yt<-round(ytn*(max-min)+min)

yt

mapet<-mean(abs((yta-yt)/yta*100))

mapet

ypn<-(predictr(model9.4,xp))

yp<-round(ypn*(max-min)+min)

yp

mapep<-mean(abs((ypa-yp)/ypa*100))

mapep

model9.5<-trainr(yf,xf,learningrate = 0.1,hidden_dim = 5,numepochs = 400,batch_size = 10,network_type = "rnn")

par(mfrow=c(1,1))

plot(colMeans(model9.5$error),type = 'l',xlab = 'Epoch',ylab = 'Errors')

ytn<-(predictr(model9.5,xt))

yt<-round(ytn*(max-min)+min)

yt

mapet<-mean(abs((yta-yt)/yta*100))

mapet

ypn<-(predictr(model9.5,xp))

yp<-round(ypn*(max-min)+min)

yp

mapep<-mean(abs((ypa-yp)/ypa*100))

mapep

model9.6<-trainr(yf,xf,learningrate = 0.1,hidden_dim = 10,numepochs = 250,batch_size = 10,network_type = "rnn")

par(mfrow=c(1,1))

plot(colMeans(model9.6$error),type = 'l',xlab = 'Epoch',ylab = 'Errors')

ytn<-(predictr(model9.6,xt))

yt<-round(ytn*(max-min)+min)

yt

mapet<-mean(abs((yta-yt)/yta*100))

mapet

ypn<-(predictr(model9.6,xp))

yp<-round(ypn*(max-min)+min)

yp

mapep<-mean(abs((ypa-yp)/ypa*100))

mapep

model9.7<-trainr(yf,xf,learningrate = 0.2,hidden_dim = 3,numepochs = 250,batch_size = 10,network_type = "rnn")

par(mfrow=c(1,1))

plot(colMeans(model9.7$error),type = 'l',xlab = 'Epoch',ylab = 'Errors')

ytn<-(predictr(model9.7,xt))

yt<-round(ytn*(max-min)+min)

yt

mapet<-mean(abs((yta-yt)/yta*100))

mapet

ypn<-(predictr(model9.7,xp))

yp<-round(ypn*(max-min)+min)

yp

mapep<-mean(abs((ypa-yp)/ypa*100))

mapep

model9.8<-trainr(yf,xf,learningrate = 0.2,hidden_dim = 5,numepochs = 200,batch_size = 10,network_type = "rnn")

par(mfrow=c(1,1))

plot(colMeans(model9.8$error),type = 'l',xlab = 'Epoch',ylab = 'Errors')

ytn<-(predictr(model9.8,xt))

yt<-round(ytn*(max-min)+min)

yt

mapet<-mean(abs((yta-yt)/yta*100))

mapet

ypn<-(predictr(model9.8,xp))

yp<-round(ypn*(max-min)+min)

yp

mapep<-mean(abs((ypa-yp)/ypa*100))

mapep

model9.9<-trainr(yf,xf,learningrate = 0.2,hidden_dim = 10,numepochs = 150,batch_size = 10,network_type = "rnn")

par(mfrow=c(1,1))

plot(colMeans(model9.9$error),type = 'l',xlab = 'Epoch',ylab = 'Errors')

ytn<-(predictr(model9.9,xt))

yt<-round(ytn*(max-min)+min)

yt

mapet<-mean(abs((yta-yt)/yta*100))

mapet

ypn<-(predictr(model9.9,xp))

yp<-round(ypn*(max-min)+min)

yp

mapep<-mean(abs((ypa-yp)/ypa*100))

mapep

##Wuxi

#1

x1<-wxrnn1$x1

y<-wxrnn1$y

max<-max(x1,y)

min<-min(x1,y)

x1n<-((x1-min)/(max-min))

yn<-((y-min)/(max-min))

xf1n<-x1n[1:143]

yfn<-yn[1:143]

xt1n<-x1n[144:155]

yta<-y[144:155]

xp1n<-x1n[156:167]

ypa<-y[156:167]

xf<-array(xf1n,dim = c(143,1,1))

yf<-array(yfn,dim = c(143,1,1))

xt<-array(xt1n,dim = c(12,1,1))

xp<-array(xp1n,dim = c(12,1,1))

model1.1<-trainr(yf,xf,learningrate = 0.05,hidden_dim = 3,numepochs = 600,batch_size = 10,network_type = "rnn")

par(mfrow=c(1,1))

plot(colMeans(model1.1$error),type = 'l',xlab = 'Epoch',ylab = 'Errors')

ytn<-(predictr(model1.1,xt))

yt<-round(ytn*(max-min)+min)

yt

mapet<-mean(abs((yta-yt)/yta*100))

mapet

ypn<-(predictr(model1.1,xp))

yp<-round(ypn*(max-min)+min)

yp

mapep<-mean(abs((ypa-yp)/ypa*100))

mapep

model1.2<-trainr(yf,xf,learningrate = 0.05,hidden_dim = 5,numepochs = 400,batch_size = 10,network_type = "rnn")

par(mfrow=c(1,1))

plot(colMeans(model1.2$error),type = 'l',xlab = 'Epoch',ylab = 'Errors')

ytn<-(predictr(model1.2,xt))

yt<-round(ytn*(max-min)+min)

yt

mapet<-mean(abs((yta-yt)/yta*100))

mapet

ypn<-(predictr(model1.2,xp))

yp<-round(ypn*(max-min)+min)

yp

mapep<-mean(abs((ypa-yp)/ypa*100))

mapep

model1.3<-trainr(yf,xf,learningrate = 0.05,hidden_dim = 10,numepochs = 300,batch_size = 10,network_type = "rnn")

par(mfrow=c(1,1))

plot(colMeans(model1.3$error),type = 'l',xlab = 'Epoch',ylab = 'Errors')

ytn<-(predictr(model1.3,xt))

yt<-round(ytn*(max-min)+min)

yt

mapet<-mean(abs((yta-yt)/yta*100))

mapet

ypn<-(predictr(model1.3,xp))

yp<-round(ypn*(max-min)+min)

yp

mapep<-mean(abs((ypa-yp)/ypa*100))

mapep

model1.4<-trainr(yf,xf,learningrate = 0.1,hidden_dim = 3,numepochs = 250,batch_size = 10,network_type = "rnn")

par(mfrow=c(1,1))

plot(colMeans(model1.4$error),type = 'l',xlab = 'Epoch',ylab = 'Errors')

ytn<-(predictr(model1.4,xt))

yt<-round(ytn*(max-min)+min)

yt

mapet<-mean(abs((yta-yt)/yta*100))

mapet

ypn<-(predictr(model1.4,xp))

yp<-round(ypn*(max-min)+min)

yp

mapep<-mean(abs((ypa-yp)/ypa*100))

mapep

model1.5<-trainr(yf,xf,learningrate = 0.1,hidden_dim = 5,numepochs = 200,batch_size = 10,network_type = "rnn")

par(mfrow=c(1,1))

plot(colMeans(model1.5$error),type = 'l',xlab = 'Epoch',ylab = 'Errors')

ytn<-(predictr(model1.5,xt))

yt<-round(ytn*(max-min)+min)

yt

mapet<-mean(abs((yta-yt)/yta*100))

mapet

ypn<-(predictr(model1.5,xp))

yp<-round(ypn*(max-min)+min)

yp

mapep<-mean(abs((ypa-yp)/ypa*100))

mapep

model1.6<-trainr(yf,xf,learningrate = 0.1,hidden_dim = 10,numepochs = 150,batch_size = 10,network_type = "rnn")

par(mfrow=c(1,1))

plot(colMeans(model1.6$error),type = 'l',xlab = 'Epoch',ylab = 'Errors')

ytn<-(predictr(model1.6,xt))

yt<-round(ytn*(max-min)+min)

yt

mapet<-mean(abs((yta-yt)/yta*100))

mapet

ypn<-(predictr(model1.6,xp))

yp<-round(ypn*(max-min)+min)

yp

mapep<-mean(abs((ypa-yp)/ypa*100))

mapep

model1.7<-trainr(yf,xf,learningrate = 0.2,hidden_dim = 3,numepochs = 150,batch_size = 10,network_type = "rnn")

par(mfrow=c(1,1))

plot(colMeans(model1.7$error),type = 'l',xlab = 'Epoch',ylab = 'Errors')

ytn<-(predictr(model1.7,xt))

yt<-round(ytn*(max-min)+min)

yt

mapet<-mean(abs((yta-yt)/yta*100))

mapet

ypn<-(predictr(model1.7,xp))

yp<-round(ypn*(max-min)+min)

yp

mapep<-mean(abs((ypa-yp)/ypa*100))

mapep

model1.8<-trainr(yf,xf,learningrate = 0.2,hidden_dim = 5,numepochs = 100,batch_size = 10,network_type = "rnn")

par(mfrow=c(1,1))

plot(colMeans(model1.8$error),type = 'l',xlab = 'Epoch',ylab = 'Errors')

ytn<-(predictr(model1.8,xt))

yt<-round(ytn*(max-min)+min)

yt

mapet<-mean(abs((yta-yt)/yta*100))

mapet

ypn<-(predictr(model1.8,xp))

yp<-round(ypn*(max-min)+min)

yp

mapep<-mean(abs((ypa-yp)/ypa*100))

mapep

model1.9<-trainr(yf,xf,learningrate = 0.2,hidden_dim = 10,numepochs = 80,batch_size = 10,network_type = "rnn")

par(mfrow=c(1,1))

plot(colMeans(model1.9$error),type = 'l',xlab = 'Epoch',ylab = 'Errors')

ytn<-(predictr(model1.9,xt))

yt<-round(ytn*(max-min)+min)

yt

mapet<-mean(abs((yta-yt)/yta*100))

mapet

ypn<-(predictr(model1.9,xp))

yp<-round(ypn*(max-min)+min)

yp

mapep<-mean(abs((ypa-yp)/ypa*100))

mapep

#2

x1<-wxrnn2$x1

x2<-wxrnn2$x2

y<-wxrnn2$y

max<-max(x1,x2,y)

min<-min(x1,x2,y)

x1n<-((x1-min)/(max-min))

x2n<-((x2-min)/(max-min))

yn<-((y-min)/(max-min))

xf1n<-x1n[1:142]

xf2n<-x2n[1:142]

yfn<-yn[1:142]

xt1n<-x1n[143:154]

xt2n<-x2n[143:154]

yta<-y[143:154]

xp1n<-x1n[155:166]

xp2n<-x2n[155:166]

ypa<-y[155:166]

xf<-array(c(xf1n,xf2n),dim = c(142,1,2))

yf<-array(yfn,dim = c(142,1,1))

xt<-array(c(xt1n,xt2n),dim = c(12,1,2))

xp<-array(c(xp1n,xp2n),dim = c(12,1,2))

model2.1<-trainr(yf,xf,learningrate = 0.05,hidden_dim = 3,numepochs = 500,batch_size = 10,network_type = "rnn")

par(mfrow=c(1,1))

plot(colMeans(model2.1$error),type = 'l',xlab = 'Epoch',ylab = 'Errors')

ytn<-(predictr(model2.1,xt))

yt<-round(ytn*(max-min)+min)

yt

mapet<-mean(abs((yta-yt)/yta*100))

mapet

ypn<-(predictr(model2.1,xp))

yp<-round(ypn*(max-min)+min)

yp

mapep<-mean(abs((ypa-yp)/ypa*100))

mapep

model2.2<-trainr(yf,xf,learningrate = 0.05,hidden_dim = 5,numepochs = 400,batch_size = 10,network_type = "rnn")

par(mfrow=c(1,1))

plot(colMeans(model2.2$error),type = 'l',xlab = 'Epoch',ylab = 'Errors')

ytn<-(predictr(model2.2,xt))

yt<-round(ytn*(max-min)+min)

yt

mapet<-mean(abs((yta-yt)/yta*100))

mapet

ypn<-(predictr(model2.2,xp))

yp<-round(ypn*(max-min)+min)

yp

mapep<-mean(abs((ypa-yp)/ypa*100))

mapep

model2.3<-trainr(yf,xf,learningrate = 0.05,hidden_dim = 10,numepochs = 250,batch_size = 10,network_type = "rnn")

par(mfrow=c(1,1))

plot(colMeans(model2.3$error),type = 'l',xlab = 'Epoch',ylab = 'Errors')

ytn<-(predictr(model2.3,xt))

yt<-round(ytn*(max-min)+min)

yt

mapet<-mean(abs((yta-yt)/yta*100))

mapet

ypn<-(predictr(model2.3,xp))

yp<-round(ypn*(max-min)+min)

yp

mapep<-mean(abs((ypa-yp)/ypa*100))

mapep

model2.4<-trainr(yf,xf,learningrate = 0.1,hidden_dim = 3,numepochs = 250,batch_size = 10,network_type = "rnn")

par(mfrow=c(1,1))

plot(colMeans(model2.4$error),type = 'l',xlab = 'Epoch',ylab = 'Errors')

ytn<-(predictr(model2.4,xt))

yt<-round(ytn*(max-min)+min)

yt

mapet<-mean(abs((yta-yt)/yta*100))

mapet

ypn<-(predictr(model2.4,xp))

yp<-round(ypn*(max-min)+min)

yp

mapep<-mean(abs((ypa-yp)/ypa*100))

mapep

model2.5<-trainr(yf,xf,learningrate = 0.1,hidden_dim = 5,numepochs = 200,batch_size = 10,network_type = "rnn")

par(mfrow=c(1,1))

plot(colMeans(model2.5$error),type = 'l',xlab = 'Epoch',ylab = 'Errors')

ytn<-(predictr(model2.5,xt))

yt<-round(ytn*(max-min)+min)

yt

mapet<-mean(abs((yta-yt)/yta*100))

mapet

ypn<-(predictr(model2.5,xp))

yp<-round(ypn*(max-min)+min)

yp

mapep<-mean(abs((ypa-yp)/ypa*100))

mapep

model2.6<-trainr(yf,xf,learningrate = 0.1,hidden_dim = 10,numepochs = 150,batch_size = 10,network_type = "rnn")

par(mfrow=c(1,1))

plot(colMeans(model2.6$error),type = 'l',xlab = 'Epoch',ylab = 'Errors')

ytn<-(predictr(model2.6,xt))

yt<-round(ytn*(max-min)+min)

yt

mapet<-mean(abs((yta-yt)/yta*100))

mapet

ypn<-(predictr(model2.6,xp))

yp<-round(ypn*(max-min)+min)

yp

mapep<-mean(abs((ypa-yp)/ypa*100))

mapep

model2.7<-trainr(yf,xf,learningrate = 0.2,hidden_dim = 3,numepochs = 150,batch_size = 10,network_type = "rnn")

par(mfrow=c(1,1))

plot(colMeans(model2.7$error),type = 'l',xlab = 'Epoch',ylab = 'Errors')

ytn<-(predictr(model2.7,xt))

yt<-round(ytn*(max-min)+min)

yt

mapet<-mean(abs((yta-yt)/yta*100))

mapet

ypn<-(predictr(model2.7,xp))

yp<-round(ypn*(max-min)+min)

yp

mapep<-mean(abs((ypa-yp)/ypa*100))

mapep

model2.8<-trainr(yf,xf,learningrate = 0.2,hidden_dim = 5,numepochs = 100,batch_size = 10,network_type = "rnn")

par(mfrow=c(1,1))

plot(colMeans(model2.8$error),type = 'l',xlab = 'Epoch',ylab = 'Errors')

ytn<-(predictr(model2.8,xt))

yt<-round(ytn*(max-min)+min)

yt

mapet<-mean(abs((yta-yt)/yta*100))

mapet

ypn<-(predictr(model2.8,xp))

yp<-round(ypn*(max-min)+min)

yp

mapep<-mean(abs((ypa-yp)/ypa*100))

mapep

model2.9<-trainr(yf,xf,learningrate = 0.2,hidden_dim = 10,numepochs = 80,batch_size = 10,network_type = "rnn")

par(mfrow=c(1,1))

plot(colMeans(model2.9$error),type = 'l',xlab = 'Epoch',ylab = 'Errors')

ytn<-(predictr(model2.9,xt))

yt<-round(ytn*(max-min)+min)

yt

mapet<-mean(abs((yta-yt)/yta*100))

mapet

ypn<-(predictr(model2.9,xp))

yp<-round(ypn*(max-min)+min)

yp

mapep<-mean(abs((ypa-yp)/ypa*100))

mapep

#3

x1<-wxrnn3$x1

x2<-wxrnn3$x2

x3<-wxrnn3$x3

y<-wxrnn3$y

max<-max(x1,x2,x3,y)

min<-min(x1,x2,x3,y)

x1n<-((x1-min)/(max-min))

x2n<-((x2-min)/(max-min))

x3n<-((x3-min)/(max-min))

yn<-((y-min)/(max-min))

xf1n<-x1n[1:141]

xf2n<-x2n[1:141]

xf3n<-x3n[1:141]

yfn<-yn[1:141]

xt1n<-x1n[142:153]

xt2n<-x2n[142:153]

xt3n<-x3n[142:153]

yta<-y[142:153]

xp1n<-x1n[154:165]

xp2n<-x2n[154:165]

xp3n<-x3n[154:165]

ypa<-y[154:165]

xf<-array(c(xf1n,xf2n,xf3n),dim = c(141,1,3))

yf<-array(yfn,dim = c(141,1,1))

xt<-array(c(xt1n,xt2n,xt3n),dim = c(12,1,3))

xp<-array(c(xp1n,xp2n,xp3n),dim = c(12,1,3))

model3.1<-trainr(yf,xf,learningrate = 0.05,hidden_dim = 3,numepochs = 500,batch_size = 10,network_type = "rnn")

par(mfrow=c(1,1))

plot(colMeans(model3.1$error),type = 'l',xlab = 'Epoch',ylab = 'Errors')

ytn<-(predictr(model3.1,xt))

yt<-round(ytn*(max-min)+min)

yt

mapet<-mean(abs((yta-yt)/yta*100))

mapet

ypn<-(predictr(model3.1,xp))

yp<-round(ypn*(max-min)+min)

yp

mapep<-mean(abs((ypa-yp)/ypa*100))

mapep

model3.2<-trainr(yf,xf,learningrate = 0.05,hidden_dim = 5,numepochs = 400,batch_size = 10,network_type = "rnn")

par(mfrow=c(1,1))

plot(colMeans(model3.2$error),type = 'l',xlab = 'Epoch',ylab = 'Errors')

ytn<-(predictr(model3.2,xt))

yt<-round(ytn*(max-min)+min)

yt

mapet<-mean(abs((yta-yt)/yta*100))

mapet

ypn<-(predictr(model3.2,xp))

yp<-round(ypn*(max-min)+min)

yp

mapep<-mean(abs((ypa-yp)/ypa*100))

mapep

model3.3<-trainr(yf,xf,learningrate = 0.05,hidden_dim = 10,numepochs = 250,batch_size = 10,network_type = "rnn")

par(mfrow=c(1,1))

plot(colMeans(model3.3$error),type = 'l',xlab = 'Epoch',ylab = 'Errors')

ytn<-(predictr(model3.3,xt))

yt<-round(ytn*(max-min)+min)

yt

mapet<-mean(abs((yta-yt)/yta*100))

mapet

ypn<-(predictr(model3.3,xp))

yp<-round(ypn*(max-min)+min)

yp

mapep<-mean(abs((ypa-yp)/ypa*100))

mapep

model3.4<-trainr(yf,xf,learningrate = 0.1,hidden_dim = 3,numepochs = 250,batch_size = 10,network_type = "rnn")

par(mfrow=c(1,1))

plot(colMeans(model3.4$error),type = 'l',xlab = 'Epoch',ylab = 'Errors')

ytn<-(predictr(model3.4,xt))

yt<-round(ytn*(max-min)+min)

yt

mapet<-mean(abs((yta-yt)/yta*100))

mapet

ypn<-(predictr(model3.4,xp))

yp<-round(ypn*(max-min)+min)

yp

mapep<-mean(abs((ypa-yp)/ypa*100))

mapep

model3.5<-trainr(yf,xf,learningrate = 0.1,hidden_dim = 5,numepochs = 200,batch_size = 10,network_type = "rnn")

par(mfrow=c(1,1))

plot(colMeans(model3.5$error),type = 'l',xlab = 'Epoch',ylab = 'Errors')

ytn<-(predictr(model3.5,xt))

yt<-round(ytn*(max-min)+min)

yt

mapet<-mean(abs((yta-yt)/yta*100))

mapet

ypn<-(predictr(model3.5,xp))

yp<-round(ypn*(max-min)+min)

yp

mapep<-mean(abs((ypa-yp)/ypa*100))

mapep

model3.6<-trainr(yf,xf,learningrate = 0.1,hidden_dim = 10,numepochs = 150,batch_size = 10,network_type = "rnn")

par(mfrow=c(1,1))

plot(colMeans(model3.6$error),type = 'l',xlab = 'Epoch',ylab = 'Errors')

ytn<-(predictr(model3.6,xt))

yt<-round(ytn*(max-min)+min)

yt

mapet<-mean(abs((yta-yt)/yta*100))

mapet

ypn<-(predictr(model3.6,xp))

yp<-round(ypn*(max-min)+min)

yp

mapep<-mean(abs((ypa-yp)/ypa*100))

mapep

model3.7<-trainr(yf,xf,learningrate = 0.2,hidden_dim = 3,numepochs = 150,batch_size = 10,network_type = "rnn")

par(mfrow=c(1,1))

plot(colMeans(model3.7$error),type = 'l',xlab = 'Epoch',ylab = 'Errors')

ytn<-(predictr(model3.7,xt))

yt<-round(ytn*(max-min)+min)

yt

mapet<-mean(abs((yta-yt)/yta*100))

mapet

ypn<-(predictr(model3.7,xp))

yp<-round(ypn*(max-min)+min)

yp

mapep<-mean(abs((ypa-yp)/ypa*100))

mapep

model3.8<-trainr(yf,xf,learningrate = 0.2,hidden_dim = 5,numepochs = 100,batch_size = 10,network_type = "rnn")

par(mfrow=c(1,1))

plot(colMeans(model3.8$error),type = 'l',xlab = 'Epoch',ylab = 'Errors')

ytn<-(predictr(model3.8,xt))

yt<-round(ytn*(max-min)+min)

yt

mapet<-mean(abs((yta-yt)/yta*100))

mapet

ypn<-(predictr(model3.8,xp))

yp<-round(ypn*(max-min)+min)

yp

mapep<-mean(abs((ypa-yp)/ypa*100))

mapep

model3.9<-trainr(yf,xf,learningrate = 0.2,hidden_dim = 10,numepochs = 80,batch_size = 10,network_type = "rnn")

par(mfrow=c(1,1))

plot(colMeans(model3.9$error),type = 'l',xlab = 'Epoch',ylab = 'Errors')

ytn<-(predictr(model3.9,xt))

yt<-round(ytn*(max-min)+min)

yt

mapet<-mean(abs((yta-yt)/yta*100))

mapet

ypn<-(predictr(model3.9,xp))

yp<-round(ypn*(max-min)+min)

yp

mapep<-mean(abs((ypa-yp)/ypa*100))

mapep

#4

x1<-wxrnn4$x1

x2<-wxrnn4$x2

x3<-wxrnn4$x3

x4<-wxrnn4$x4

x5<-wxrnn4$x5

x6<-wxrnn4$x6

y<-wxrnn4$y

max<-max(x1,x2,x3,x4,x5,x6,y)

min<-min(x1,x2,x3,x4,x5,x6,y)

x1n<-((x1-min)/(max-min))

x2n<-((x2-min)/(max-min))

x3n<-((x3-min)/(max-min))

x4n<-((x4-min)/(max-min))

x5n<-((x5-min)/(max-min))

x6n<-((x6-min)/(max-min))

yn<-((y-min)/(max-min))

xf1n<-x1n[1:138]

xf2n<-x2n[1:138]

xf3n<-x3n[1:138]

xf4n<-x4n[1:138]

xf5n<-x5n[1:138]

xf6n<-x6n[1:138]

yfn<-yn[1:138]

xt1n<-x1n[139:150]

xt2n<-x2n[139:150]

xt3n<-x3n[139:150]

xt4n<-x4n[139:150]

xt5n<-x5n[139:150]

xt6n<-x6n[139:150]

yta<-y[139:150]

xp1n<-x1n[151:162]

xp2n<-x2n[151:162]

xp3n<-x3n[151:162]

xp4n<-x4n[151:162]

xp5n<-x5n[151:162]

xp6n<-x6n[151:162]

ypa<-y[151:162]

xf<-array(c(xf1n,xf2n,xf3n,xf4n,xf5n,xf6n),dim = c(138,1,6))

yf<-array(yfn,dim = c(138,1,1))

xt<-array(c(xt1n,xt2n,xt3n,xt4n,xt5n,xt6n),dim = c(12,1,6))

xp<-array(c(xp1n,xp2n,xp3n,xp4n,xp5n,xp6n),dim = c(12,1,6))

model4.1<-trainr(yf,xf,learningrate = 0.05,hidden_dim = 3,numepochs = 600,batch_size = 10,network_type = "rnn")

par(mfrow=c(1,1))

plot(colMeans(model4.1$error),type = 'l',xlab = 'Epoch',ylab = 'Errors')

ytn<-(predictr(model4.1,xt))

yt<-round(ytn*(max-min)+min)

yt

mapet<-mean(abs((yta-yt)/yta*100))

mapet

ypn<-(predictr(model4.1,xp))

yp<-round(ypn*(max-min)+min)

yp

mapep<-mean(abs((ypa-yp)/ypa*100))

mapep

model4.2<-trainr(yf,xf,learningrate = 0.05,hidden_dim = 5,numepochs = 500,batch_size = 10,network_type = "rnn")

par(mfrow=c(1,1))

plot(colMeans(model4.2$error),type = 'l',xlab = 'Epoch',ylab = 'Errors')

ytn<-(predictr(model4.2,xt))

yt<-round(ytn*(max-min)+min)

yt

mapet<-mean(abs((yta-yt)/yta*100))

mapet

ypn<-(predictr(model4.2,xp))

yp<-round(ypn*(max-min)+min)

yp

mapep<-mean(abs((ypa-yp)/ypa*100))

mapep

model4.3<-trainr(yf,xf,learningrate = 0.05,hidden_dim = 10,numepochs = 400,batch_size = 10,network_type = "rnn")

par(mfrow=c(1,1))

plot(colMeans(model4.3$error),type = 'l',xlab = 'Epoch',ylab = 'Errors')

ytn<-(predictr(model4.3,xt))

yt<-round(ytn*(max-min)+min)

yt

mapet<-mean(abs((yta-yt)/yta*100))

mapet

ypn<-(predictr(model4.3,xp))

yp<-round(ypn*(max-min)+min)

yp

mapep<-mean(abs((ypa-yp)/ypa*100))

mapep

model4.4<-trainr(yf,xf,learningrate = 0.1,hidden_dim = 3,numepochs = 300,batch_size = 10,network_type = "rnn")

par(mfrow=c(1,1))

plot(colMeans(model4.4$error),type = 'l',xlab = 'Epoch',ylab = 'Errors')

ytn<-(predictr(model4.4,xt))

yt<-round(ytn*(max-min)+min)

yt

mapet<-mean(abs((yta-yt)/yta*100))

mapet

ypn<-(predictr(model4.4,xp))

yp<-round(ypn*(max-min)+min)

yp

mapep<-mean(abs((ypa-yp)/ypa*100))

mapep

model4.5<-trainr(yf,xf,learningrate = 0.1,hidden_dim = 5,numepochs = 250,batch_size = 10,network_type = "rnn")

par(mfrow=c(1,1))

plot(colMeans(model4.5$error),type = 'l',xlab = 'Epoch',ylab = 'Errors')

ytn<-(predictr(model4.5,xt))

yt<-round(ytn*(max-min)+min)

yt

mapet<-mean(abs((yta-yt)/yta*100))

mapet

ypn<-(predictr(model4.5,xp))

yp<-round(ypn*(max-min)+min)

yp

mapep<-mean(abs((ypa-yp)/ypa*100))

mapep

model4.6<-trainr(yf,xf,learningrate = 0.1,hidden_dim = 10,numepochs = 200,batch_size = 10,network_type = "rnn")

par(mfrow=c(1,1))

plot(colMeans(model4.6$error),type = 'l',xlab = 'Epoch',ylab = 'Errors')

ytn<-(predictr(model4.6,xt))

yt<-round(ytn*(max-min)+min)

yt

mapet<-mean(abs((yta-yt)/yta*100))

mapet

ypn<-(predictr(model4.6,xp))

yp<-round(ypn*(max-min)+min)

yp

mapep<-mean(abs((ypa-yp)/ypa*100))

mapep

model4.7<-trainr(yf,xf,learningrate = 0.2,hidden_dim = 3,numepochs = 150,batch_size = 10,network_type = "rnn")

par(mfrow=c(1,1))

plot(colMeans(model4.7$error),type = 'l',xlab = 'Epoch',ylab = 'Errors')

ytn<-(predictr(model4.7,xt))

yt<-round(ytn*(max-min)+min)

yt

mapet<-mean(abs((yta-yt)/yta*100))

mapet

ypn<-(predictr(model4.7,xp))

yp<-round(ypn*(max-min)+min)

yp

mapep<-mean(abs((ypa-yp)/ypa*100))

mapep

model4.8<-trainr(yf,xf,learningrate = 0.2,hidden_dim = 5,numepochs = 100,batch_size = 10,network_type = "rnn")

par(mfrow=c(1,1))

plot(colMeans(model4.8$error),type = 'l',xlab = 'Epoch',ylab = 'Errors')

ytn<-(predictr(model4.8,xt))

yt<-round(ytn*(max-min)+min)

yt

mapet<-mean(abs((yta-yt)/yta*100))

mapet

ypn<-(predictr(model4.8,xp))

yp<-round(ypn*(max-min)+min)

yp

mapep<-mean(abs((ypa-yp)/ypa*100))

mapep

model4.9<-trainr(yf,xf,learningrate = 0.2,hidden_dim = 10,numepochs = 80,batch_size = 10,network_type = "rnn")

par(mfrow=c(1,1))

plot(colMeans(model4.9$error),type = 'l',xlab = 'Epoch',ylab = 'Errors')

ytn<-(predictr(model4.9,xt))

yt<-round(ytn*(max-min)+min)

yt

mapet<-mean(abs((yta-yt)/yta*100))

mapet

ypn<-(predictr(model4.9,xp))

yp<-round(ypn*(max-min)+min)

yp

mapep<-mean(abs((ypa-yp)/ypa*100))

mapep

#5

x1<-wxrnn5$x1

x2<-wxrnn5$x2

x3<-wxrnn5$x3

x4<-wxrnn5$x4

x5<-wxrnn5$x5

x6<-wxrnn5$x6

x7<-wxrnn5$x7

x8<-wxrnn5$x8

x9<-wxrnn5$x9

x10<-wxrnn5$x10

x11<-wxrnn5$x11

x12<-wxrnn5$x12

y<-wxrnn5$y

max<-max(x1,x2,x3,x4,x5,x6,x7,x8,x9,x10,x11,x12,y)

min<-min(x1,x2,x3,x4,x5,x6,x7,x8,x9,x10,x11,x12,y)

x1n<-((x1-min)/(max-min))

x2n<-((x2-min)/(max-min))

x3n<-((x3-min)/(max-min))

x4n<-((x4-min)/(max-min))

x5n<-((x5-min)/(max-min))

x6n<-((x6-min)/(max-min))

x7n<-((x7-min)/(max-min))

x8n<-((x8-min)/(max-min))

x9n<-((x9-min)/(max-min))

x10n<-((x10-min)/(max-min))

x11n<-((x11-min)/(max-min))

x12n<-((x12-min)/(max-min))

yn<-((y-min)/(max-min))

xf1n<-x1n[1:132]

xf2n<-x2n[1:132]

xf3n<-x3n[1:132]

xf4n<-x4n[1:132]

xf5n<-x5n[1:132]

xf6n<-x6n[1:132]

xf7n<-x7n[1:132]

xf8n<-x8n[1:132]

xf9n<-x9n[1:132]

xf10n<-x10n[1:132]

xf11n<-x11n[1:132]

xf12n<-x12n[1:132]

yfn<-yn[1:132]

xt1n<-x1n[133:144]

xt2n<-x2n[133:144]

xt3n<-x3n[133:144]

xt4n<-x4n[133:144]

xt5n<-x5n[133:144]

xt6n<-x6n[133:144]

xt7n<-x7n[133:144]

xt8n<-x8n[133:144]

xt9n<-x9n[133:144]

xt10n<-x10n[133:144]

xt11n<-x11n[133:144]

xt12n<-x12n[133:144]

yta<-y[133:144]

xp1n<-x1n[145:156]

xp2n<-x2n[145:156]

xp3n<-x3n[145:156]

xp4n<-x4n[145:156]

xp5n<-x5n[145:156]

xp6n<-x6n[145:156]

xp7n<-x7n[145:156]

xp8n<-x8n[145:156]

xp9n<-x9n[145:156]

xp10n<-x10n[145:156]

xp11n<-x11n[145:156]

xp12n<-x12n[145:156]

ypa<-y[145:156]

xf<-array(c(xf1n,xf2n,xf3n,xf4n,xf5n,xf6n,xf7n,xf8n,xf9n,xf10n,xf11n,xf12n),dim = c(132,1,12))

yf<-array(yfn,dim = c(132,1,1))

xt<-array(c(xt1n,xt2n,xt3n,xt4n,xt5n,xt6n,xt7n,xt8n,xt9n,xt10n,xt11n,xt12n),dim = c(12,1,12))

xp<-array(c(xp1n,xp2n,xp3n,xp4n,xp5n,xp6n,xp7n,xp8n,xp9n,xp10n,xp11n,xp12n),dim = c(12,1,12))

model5.1<-trainr(yf,xf,learningrate = 0.05,hidden_dim = 3,numepochs = 800,batch_size = 10,network_type = "rnn")

par(mfrow=c(1,1))

plot(colMeans(model5.1$error),type = 'l',xlab = 'Epoch',ylab = 'Errors')

ytn<-(predictr(model5.1,xt))

yt<-round(ytn*(max-min)+min)

yt

mapet<-mean(abs((yta-yt)/yta*100))

mapet

ypn<-(predictr(model5.1,xp))

yp<-round(ypn*(max-min)+min)

yp

mapep<-mean(abs((ypa-yp)/ypa*100))

mapep

model5.2<-trainr(yf,xf,learningrate = 0.05,hidden_dim = 5,numepochs = 600,batch_size = 10,network_type = "rnn")

par(mfrow=c(1,1))

plot(colMeans(model5.2$error),type = 'l',xlab = 'Epoch',ylab = 'Errors')

ytn<-(predictr(model5.2,xt))

yt<-round(ytn*(max-min)+min)

yt

mapet<-mean(abs((yta-yt)/yta*100))

mapet

ypn<-(predictr(model5.2,xp))

yp<-round(ypn*(max-min)+min)

yp

mapep<-mean(abs((ypa-yp)/ypa*100))

mapep

model5.3<-trainr(yf,xf,learningrate = 0.05,hidden_dim = 10,numepochs = 400,batch_size = 10,network_type = "rnn")

par(mfrow=c(1,1))

plot(colMeans(model5.3$error),type = 'l',xlab = 'Epoch',ylab = 'Errors')

ytn<-(predictr(model5.3,xt))

yt<-round(ytn*(max-min)+min)

yt

mapet<-mean(abs((yta-yt)/yta*100))

mapet

ypn<-(predictr(model5.3,xp))

yp<-round(ypn*(max-min)+min)

yp

mapep<-mean(abs((ypa-yp)/ypa*100))

mapep

model5.4<-trainr(yf,xf,learningrate = 0.1,hidden_dim = 3,numepochs = 400,batch_size = 10,network_type = "rnn")

par(mfrow=c(1,1))

plot(colMeans(model5.4$error),type = 'l',xlab = 'Epoch',ylab = 'Errors')

ytn<-(predictr(model5.4,xt))

yt<-round(ytn*(max-min)+min)

yt

mapet<-mean(abs((yta-yt)/yta*100))

mapet

ypn<-(predictr(model5.4,xp))

yp<-round(ypn*(max-min)+min)

yp

mapep<-mean(abs((ypa-yp)/ypa*100))

mapep

model5.5<-trainr(yf,xf,learningrate = 0.1,hidden_dim = 5,numepochs = 300,batch_size = 10,network_type = "rnn")

par(mfrow=c(1,1))

plot(colMeans(model5.5$error),type = 'l',xlab = 'Epoch',ylab = 'Errors')

ytn<-(predictr(model5.5,xt))

yt<-round(ytn*(max-min)+min)

yt

mapet<-mean(abs((yta-yt)/yta*100))

mapet

ypn<-(predictr(model5.5,xp))

yp<-round(ypn*(max-min)+min)

yp

mapep<-mean(abs((ypa-yp)/ypa*100))

mapep

model5.6<-trainr(yf,xf,learningrate = 0.1,hidden_dim = 10,numepochs = 200,batch_size = 10,network_type = "rnn")

par(mfrow=c(1,1))

plot(colMeans(model5.6$error),type = 'l',xlab = 'Epoch',ylab = 'Errors')

ytn<-(predictr(model5.6,xt))

yt<-round(ytn*(max-min)+min)

yt

mapet<-mean(abs((yta-yt)/yta*100))

mapet

ypn<-(predictr(model5.6,xp))

yp<-round(ypn*(max-min)+min)

yp

mapep<-mean(abs((ypa-yp)/ypa*100))

mapep

model5.7<-trainr(yf,xf,learningrate = 0.2,hidden_dim = 3,numepochs = 150,batch_size = 10,network_type = "rnn")

par(mfrow=c(1,1))

plot(colMeans(model5.7$error),type = 'l',xlab = 'Epoch',ylab = 'Errors')

ytn<-(predictr(model5.7,xt))

yt<-round(ytn*(max-min)+min)

yt

mapet<-mean(abs((yta-yt)/yta*100))

mapet

ypn<-(predictr(model5.7,xp))

yp<-round(ypn*(max-min)+min)

yp

mapep<-mean(abs((ypa-yp)/ypa*100))

mapep

model5.8<-trainr(yf,xf,learningrate = 0.2,hidden_dim = 5,numepochs = 100,batch_size = 10,network_type = "rnn")

par(mfrow=c(1,1))

plot(colMeans(model5.8$error),type = 'l',xlab = 'Epoch',ylab = 'Errors')

ytn<-(predictr(model5.8,xt))

yt<-round(ytn*(max-min)+min)

yt

mapet<-mean(abs((yta-yt)/yta*100))

mapet

ypn<-(predictr(model5.8,xp))

yp<-round(ypn*(max-min)+min)

yp

mapep<-mean(abs((ypa-yp)/ypa*100))

mapep

model5.9<-trainr(yf,xf,learningrate = 0.2,hidden_dim = 10,numepochs = 80,batch_size = 10,network_type = "rnn")

par(mfrow=c(1,1))

plot(colMeans(model5.9$error),type = 'l',xlab = 'Epoch',ylab = 'Errors')

ytn<-(predictr(model5.9,xt))

yt<-round(ytn*(max-min)+min)

yt

mapet<-mean(abs((yta-yt)/yta*100))

mapet

ypn<-(predictr(model5.9,xp))

yp<-round(ypn*(max-min)+min)

yp

mapep<-mean(abs((ypa-yp)/ypa*100))

mapep

#6

x1<-wxrnn6$x1

x2<-wxrnn6$x2

x3<-wxrnn6$x3

x4<-wxrnn6$x4

x5<-wxrnn6$x5

x6<-wxrnn6$x6

x7<-wxrnn6$x7

x8<-wxrnn6$x8

x9<-wxrnn6$x9

x10<-wxrnn6$x10

x11<-wxrnn6$x11

x12<-wxrnn6$x12

x13<-wxrnn6$x13

x14<-(wxrnn6$x14)/10

x15<-wxrnn6$x15

x16<-wxrnn6$x16

x17<-wxrnn6$x17

y<-wxrnn6$y

max<-max(x1,x2,x3,x4,x5,x6,x7,x8,x9,x10,x11,x12,x13,x14,x15,x16,x17,y)

min<-min(x1,x2,x3,x4,x5,x6,x7,x8,x9,x10,x11,x12,x13,x14,x15,x16,x17,y)

x1n<-((x1-min)/(max-min))

x2n<-((x2-min)/(max-min))

x3n<-((x3-min)/(max-min))

x4n<-((x4-min)/(max-min))

x5n<-((x5-min)/(max-min))

x6n<-((x6-min)/(max-min))

x7n<-((x7-min)/(max-min))

x8n<-((x8-min)/(max-min))

x9n<-((x9-min)/(max-min))

x10n<-((x10-min)/(max-min))

x11n<-((x11-min)/(max-min))

x12n<-((x12-min)/(max-min))

x13n<-((x13-min)/(max-min))

x14n<-((x14-min)/(max-min))

x15n<-((x15-min)/(max-min))

x16n<-((x16-min)/(max-min))

x17n<-((x17-min)/(max-min))

yn<-((y-min)/(max-min))

xf1n<-x1n[1:132]

xf2n<-x2n[1:132]

xf3n<-x3n[1:132]

xf4n<-x4n[1:132]

xf5n<-x5n[1:132]

xf6n<-x6n[1:132]

xf7n<-x7n[1:132]

xf8n<-x8n[1:132]

xf9n<-x9n[1:132]

xf10n<-x10n[1:132]

xf11n<-x11n[1:132]

xf12n<-x12n[1:132]

xf13n<-x13n[1:132]

xf14n<-x14n[1:132]

xf15n<-x15n[1:132]

xf16n<-x16n[1:132]

xf17n<-x17n[1:132]

yfn<-yn[1:132]

xt1n<-x1n[133:144]

xt2n<-x2n[133:144]

xt3n<-x3n[133:144]

xt4n<-x4n[133:144]

xt5n<-x5n[133:144]

xt6n<-x6n[133:144]

xt7n<-x7n[133:144]

xt8n<-x8n[133:144]

xt9n<-x9n[133:144]

xt10n<-x10n[133:144]

xt11n<-x11n[133:144]

xt12n<-x12n[133:144]

xt13n<-x13n[133:144]

xt14n<-x14n[133:144]

xt15n<-x15n[133:144]

xt16n<-x16n[133:144]

xt17n<-x17n[133:144]

yta<-y[133:144]

xp1n<-x1n[145:156]

xp2n<-x2n[145:156]

xp3n<-x3n[145:156]

xp4n<-x4n[145:156]

xp5n<-x5n[145:156]

xp6n<-x6n[145:156]

xp7n<-x7n[145:156]

xp8n<-x8n[145:156]

xp9n<-x9n[145:156]

xp10n<-x10n[145:156]

xp11n<-x11n[145:156]

xp12n<-x12n[145:156]

xp13n<-x13n[145:156]

xp14n<-x14n[145:156]

xp15n<-x15n[145:156]

xp16n<-x16n[145:156]

xp17n<-x17n[145:156]

ypa<-y[145:156]

xf<-array(c(xf1n,xf2n,xf3n,xf4n,xf5n,xf6n,xf7n,xf8n,xf9n,xf10n,xf11n,xf12n,xf13n,

xf14n,xf15n,xf16n,xf17n),dim = c(132,1,17))

yf<-array(yfn,dim = c(132,1,1))

xt<-array(c(xt1n,xt2n,xt3n,xt4n,xt5n,xt6n,xt7n,xt8n,xt9n,xt10n,xt11n,xt12n,xt13n,

xt14n,xt15n,xt16n,xt17n),dim = c(12,1,17))

xp<-array(c(xp1n,xp2n,xp3n,xp4n,xp5n,xp6n,xp7n,xp8n,xp9n,xp10n,xp11n,xp12n,xp13n,

xp14n,xp15n,xp16n,xp17n),dim = c(12,1,17))

model6.1<-trainr(yf,xf,learningrate = 0.05,hidden_dim = 3,numepochs = 1500,batch_size = 10,network_type = "rnn")

par(mfrow=c(1,1))

plot(colMeans(model6.1$error),type = 'l',xlab = 'Epoch',ylab = 'Errors')

ytn<-(predictr(model6.1,xt))

yt<-round(ytn*(max-min)+min)

yt

mapet<-mean(abs((yta-yt)/yta*100))

mapet

ypn<-(predictr(model6.1,xp))

yp<-round(ypn*(max-min)+min)

yp

mapep<-mean(abs((ypa-yp)/ypa*100))

mapep

model6.2<-trainr(yf,xf,learningrate = 0.05,hidden_dim = 5,numepochs = 1200,batch_size = 10,network_type = "rnn")

par(mfrow=c(1,1))

plot(colMeans(model6.2$error),type = 'l',xlab = 'Epoch',ylab = 'Errors')

ytn<-(predictr(model6.2,xt))

yt<-round(ytn*(max-min)+min)

yt

mapet<-mean(abs((yta-yt)/yta*100))

mapet

ypn<-(predictr(model6.2,xp))

yp<-round(ypn*(max-min)+min)

yp

mapep<-mean(abs((ypa-yp)/ypa*100))

mapep

model6.3<-trainr(yf,xf,learningrate = 0.05,hidden_dim = 10,numepochs = 1000,batch_size = 10,network_type = "rnn")

par(mfrow=c(1,1))

plot(colMeans(model6.3$error),type = 'l',xlab = 'Epoch',ylab = 'Errors')

ytn<-(predictr(model6.3,xt))

yt<-round(ytn*(max-min)+min)

yt

mapet<-mean(abs((yta-yt)/yta*100))

mapet

ypn<-(predictr(model6.3,xp))

yp<-round(ypn*(max-min)+min)

yp

mapep<-mean(abs((ypa-yp)/ypa*100))

mapep

model6.4<-trainr(yf,xf,learningrate = 0.1,hidden_dim = 3,numepochs = 1000,batch_size = 10,network_type = "rnn")

par(mfrow=c(1,1))

plot(colMeans(model6.4$error),type = 'l',xlab = 'Epoch',ylab = 'Errors')

ytn<-(predictr(model6.4,xt))

yt<-round(ytn*(max-min)+min)

yt

mapet<-mean(abs((yta-yt)/yta*100))

mapet

ypn<-(predictr(model6.4,xp))

yp<-round(ypn*(max-min)+min)

yp

mapep<-mean(abs((ypa-yp)/ypa*100))

mapep

model6.5<-trainr(yf,xf,learningrate = 0.1,hidden_dim = 5,numepochs = 800,batch_size = 10,network_type = "rnn")

par(mfrow=c(1,1))

plot(colMeans(model6.5$error),type = 'l',xlab = 'Epoch',ylab = 'Errors')

ytn<-(predictr(model6.5,xt))

yt<-round(ytn*(max-min)+min)

yt

mapet<-mean(abs((yta-yt)/yta*100))

mapet

ypn<-(predictr(model6.5,xp))

yp<-round(ypn*(max-min)+min)

yp

mapep<-mean(abs((ypa-yp)/ypa*100))

mapep

model6.6<-trainr(yf,xf,learningrate = 0.1,hidden_dim = 10,numepochs = 500,batch_size = 10,network_type = "rnn")

par(mfrow=c(1,1))

plot(colMeans(model6.6$error),type = 'l',xlab = 'Epoch',ylab = 'Errors')

ytn<-(predictr(model6.6,xt))

yt<-round(ytn*(max-min)+min)

yt

mapet<-mean(abs((yta-yt)/yta*100))

mapet

ypn<-(predictr(model6.6,xp))

yp<-round(ypn*(max-min)+min)

yp

mapep<-mean(abs((ypa-yp)/ypa*100))

mapep

model6.7<-trainr(yf,xf,learningrate = 0.2,hidden_dim = 3,numepochs = 400,batch_size = 10,network_type = "rnn")

par(mfrow=c(1,1))

plot(colMeans(model6.7$error),type = 'l',xlab = 'Epoch',ylab = 'Errors')

ytn<-(predictr(model6.7,xt))

yt<-round(ytn*(max-min)+min)

yt

mapet<-mean(abs((yta-yt)/yta*100))

mapet

ypn<-(predictr(model6.7,xp))

yp<-round(ypn*(max-min)+min)

yp

mapep<-mean(abs((ypa-yp)/ypa*100))

mapep

model6.8<-trainr(yf,xf,learningrate = 0.2,hidden_dim = 5,numepochs = 300,batch_size = 10,network_type = "rnn")

par(mfrow=c(1,1))

plot(colMeans(model6.8$error),type = 'l',xlab = 'Epoch',ylab = 'Errors')

ytn<-(predictr(model6.8,xt))

yt<-round(ytn*(max-min)+min)

yt

mapet<-mean(abs((yta-yt)/yta*100))

mapet

ypn<-(predictr(model6.8,xp))

yp<-round(ypn*(max-min)+min)

yp

mapep<-mean(abs((ypa-yp)/ypa*100))

mapep

model6.9<-trainr(yf,xf,learningrate = 0.2,hidden_dim = 10,numepochs = 200,batch_size = 10,network_type = "rnn")

par(mfrow=c(1,1))

plot(colMeans(model6.9$error),type = 'l',xlab = 'Epoch',ylab = 'Errors')

ytn<-(predictr(model6.9,xt))

yt<-round(ytn*(max-min)+min)

yt

mapet<-mean(abs((yta-yt)/yta*100))

mapet

ypn<-(predictr(model6.9,xp))

yp<-round(ypn*(max-min)+min)

yp

mapep<-mean(abs((ypa-yp)/ypa*100))

mapep

#7

x1<-wxrnn7$x1

x2<-wxrnn7$x2

x3<-wxrnn7$x3

x4<-wxrnn7$x4

x5<-wxrnn7$x5

x6<-wxrnn7$x6

x7<-wxrnn7$x7

x8<-wxrnn7$x8

x9<-wxrnn7$x9

x10<-wxrnn7$x10

x11<-wxrnn7$x11

x12<-wxrnn7$x12

x13<-wxrnn7$x13

y<-wxrnn7$y

max<-max(x1,x2,x3,x4,x5,x6,x7,x8,x9,x10,x11,x12,x13,y)

min<-min(x1,x2,x3,x4,x5,x6,x7,x8,x9,x10,x11,x12,x13,y)

x1n<-((x1-min)/(max-min))

x2n<-((x2-min)/(max-min))

x3n<-((x3-min)/(max-min))

x4n<-((x4-min)/(max-min))

x5n<-((x5-min)/(max-min))

x6n<-((x6-min)/(max-min))

x7n<-((x7-min)/(max-min))

x8n<-((x8-min)/(max-min))

x9n<-((x9-min)/(max-min))

x10n<-((x10-min)/(max-min))

x11n<-((x11-min)/(max-min))

x12n<-((x12-min)/(max-min))

x13n<-((x13-min)/(max-min))

yn<-((y-min)/(max-min))

xf1n<-x1n[1:132]

xf2n<-x2n[1:132]

xf3n<-x3n[1:132]

xf4n<-x4n[1:132]

xf5n<-x5n[1:132]

xf6n<-x6n[1:132]

xf7n<-x7n[1:132]

xf8n<-x8n[1:132]

xf9n<-x9n[1:132]

xf10n<-x10n[1:132]

xf11n<-x11n[1:132]

xf12n<-x12n[1:132]

xf13n<-x13n[1:132]

yfn<-yn[1:132]

xt1n<-x1n[133:144]

xt2n<-x2n[133:144]

xt3n<-x3n[133:144]

xt4n<-x4n[133:144]

xt5n<-x5n[133:144]

xt6n<-x6n[133:144]

xt7n<-x7n[133:144]

xt8n<-x8n[133:144]

xt9n<-x9n[133:144]

xt10n<-x10n[133:144]

xt11n<-x11n[133:144]

xt12n<-x12n[133:144]

xt13n<-x13n[133:144]

yta<-y[133:144]

xp1n<-x1n[145:156]

xp2n<-x2n[145:156]

xp3n<-x3n[145:156]

xp4n<-x4n[145:156]

xp5n<-x5n[145:156]

xp6n<-x6n[145:156]

xp7n<-x7n[145:156]

xp8n<-x8n[145:156]

xp9n<-x9n[145:156]

xp10n<-x10n[145:156]

xp11n<-x11n[145:156]

xp12n<-x12n[145:156]

xp13n<-x13n[145:156]

ypa<-y[145:156]

xf<-array(c(xf1n,xf2n,xf3n,xf4n,xf5n,xf6n,xf7n,xf8n,xf9n,xf10n,xf11n,xf12n,xf13n),dim = c(132,1,13))

yf<-array(yfn,dim = c(132,1,1))

xt<-array(c(xt1n,xt2n,xt3n,xt4n,xt5n,xt6n,xt7n,xt8n,xt9n,xt10n,xt11n,xt12n,xt13n),dim = c(12,1,13))

xp<-array(c(xp1n,xp2n,xp3n,xp4n,xp5n,xp6n,xp7n,xp8n,xp9n,xp10n,xp11n,xp12n,xp13n),dim = c(12,1,13))

model7.1<-trainr(yf,xf,learningrate = 0.05,hidden_dim = 3,numepochs = 1500,batch_size = 10,network_type = "rnn")

par(mfrow=c(1,1))

plot(colMeans(model7.1$error),type = 'l',xlab = 'Epoch',ylab = 'Errors')

ytn<-(predictr(model7.1,xt))

yt<-round(ytn*(max-min)+min)

yt

mapet<-mean(abs((yta-yt)/yta*100))

mapet

ypn<-(predictr(model7.1,xp))

yp<-round(ypn*(max-min)+min)

yp

mapep<-mean(abs((ypa-yp)/ypa*100))

mapep

model7.2<-trainr(yf,xf,learningrate = 0.05,hidden_dim = 5,numepochs = 1400,batch_size = 10,network_type = "rnn")

par(mfrow=c(1,1))

plot(colMeans(model7.2$error),type = 'l',xlab = 'Epoch',ylab = 'Errors')

ytn<-(predictr(model7.2,xt))

yt<-round(ytn*(max-min)+min)

yt

mapet<-mean(abs((yta-yt)/yta*100))

mapet

ypn<-(predictr(model7.2,xp))

yp<-round(ypn*(max-min)+min)

yp

mapep<-mean(abs((ypa-yp)/ypa*100))

mapep

model7.3<-trainr(yf,xf,learningrate = 0.05,hidden_dim = 10,numepochs = 1200,batch_size = 10,network_type = "rnn")

par(mfrow=c(1,1))

plot(colMeans(model7.3$error),type = 'l',xlab = 'Epoch',ylab = 'Errors')

ytn<-(predictr(model7.3,xt))

yt<-round(ytn*(max-min)+min)

yt

mapet<-mean(abs((yta-yt)/yta*100))

mapet

ypn<-(predictr(model7.3,xp))

yp<-round(ypn*(max-min)+min)

yp

mapep<-mean(abs((ypa-yp)/ypa*100))

mapep

model7.4<-trainr(yf,xf,learningrate = 0.1,hidden_dim = 3,numepochs = 1000,batch_size = 10,network_type = "rnn")

par(mfrow=c(1,1))

plot(colMeans(model7.4$error),type = 'l',xlab = 'Epoch',ylab = 'Errors')

ytn<-(predictr(model7.4,xt))

yt<-round(ytn*(max-min)+min)

yt

mapet<-mean(abs((yta-yt)/yta*100))

mapet

ypn<-(predictr(model7.4,xp))

yp<-round(ypn*(max-min)+min)

yp

mapep<-mean(abs((ypa-yp)/ypa*100))

mapep

model7.5.1<-trainr(yf,xf,learningrate = 0.1,hidden_dim = 5,numepochs = 800,batch_size = 10,network_type = "rnn")

par(mfrow=c(1,1))

plot(colMeans(model7.5.1$error),type = 'l',xlab = 'Epoch',ylab = 'Errors',main = 'C1')

ytn1<-(predictr(model7.5.1,xt))

yt1<-round(ytn1*(max-min)+min)

yt1

mapet1<-mean(abs((yta-yt1)/yta*100))

mapet1

ypn1<-(predictr(model7.5.1,xp))

yp1<-round(ypn1*(max-min)+min)

yp1

mapep1<-mean(abs((ypa-yp1)/ypa*100))

mapep1

model7.5.2<-trainr(yf,xf,learningrate = 0.1,hidden_dim = 5,numepochs = 800,batch_size = 10,network_type = "rnn")

par(mfrow=c(1,1))

plot(colMeans(model7.5.2$error),type = 'l',xlab = 'Epoch',ylab = 'Errors',main = 'C2')

ytn2<-(predictr(model7.5.2,xt))

yt2<-round(ytn2*(max-min)+min)

yt2

mapet2<-mean(abs((yta-yt2)/yta*100))

mapet2

ypn2<-(predictr(model7.5.2,xp))

yp2<-round(ypn2*(max-min)+min)

yp2

mapep2<-mean(abs((ypa-yp2)/ypa*100))

mapep2

yyn2<-(predictr(model7.5.2,xf))

yy2<-round(yyn2*(max-min)+min)

yy2

model7.5.3<-trainr(yf,xf,learningrate = 0.1,hidden_dim = 5,numepochs = 800,batch_size = 10,network_type = "rnn")

par(mfrow=c(1,1))

plot(colMeans(model7.5.3$error),type = 'l',xlab = 'Epoch',ylab = 'Errors',main = 'C3')

ytn3<-(predictr(model7.5.3,xt))

yt3<-round(ytn3*(max-min)+min)

yt3

mapet3<-mean(abs((yta-yt3)/yta*100))

mapet3

ypn3<-(predictr(model7.5.3,xp))

yp3<-round(ypn3*(max-min)+min)

yp3

mapep3<-mean(abs((ypa-yp3)/ypa*100))

mapep3

par(mfrow=c(1,3))

plot(colMeans(model7.5.1$error),type = 'l',xlab = 'Epoch',ylab = 'Errors',main = 'C1')

plot(colMeans(model7.5.2$error),type = 'l',xlab = 'Epoch',ylab = 'Errors',main = 'C2')

plot(colMeans(model7.5.3$error),type = 'l',xlab = 'Epoch',ylab = 'Errors',main = 'C3')

model7.6<-trainr(yf,xf,learningrate = 0.1,hidden_dim = 10,numepochs = 500,batch_size = 10,network_type = "rnn")

par(mfrow=c(1,1))

plot(colMeans(model7.6$error),type = 'l',xlab = 'Epoch',ylab = 'Errors')

ytn<-(predictr(model7.6,xt))

yt<-round(ytn*(max-min)+min)

yt

mapet<-mean(abs((yta-yt)/yta*100))

mapet

ypn<-(predictr(model7.6,xp))

yp<-round(ypn*(max-min)+min)

yp

mapep<-mean(abs((ypa-yp)/ypa*100))

mapep

model7.7<-trainr(yf,xf,learningrate = 0.2,hidden_dim = 3,numepochs = 400,batch_size = 10,network_type = "rnn")

par(mfrow=c(1,1))

plot(colMeans(model7.7$error),type = 'l',xlab = 'Epoch',ylab = 'Errors')

ytn<-(predictr(model7.7,xt))

yt<-round(ytn*(max-min)+min)

yt

mapet<-mean(abs((yta-yt)/yta*100))

mapet

ypn<-(predictr(model7.7,xp))

yp<-round(ypn*(max-min)+min)

yp

mapep<-mean(abs((ypa-yp)/ypa*100))

mapep

model7.8<-trainr(yf,xf,learningrate = 0.2,hidden_dim = 5,numepochs = 300,batch_size = 10,network_type = "rnn")

par(mfrow=c(1,1))

plot(colMeans(model7.8$error),type = 'l',xlab = 'Epoch',ylab = 'Errors')

ytn<-(predictr(model7.8,xt))

yt<-round(ytn*(max-min)+min)

yt

mapet<-mean(abs((yta-yt)/yta*100))

mapet

ypn<-(predictr(model7.8,xp))

yp<-round(ypn*(max-min)+min)

yp

mapep<-mean(abs((ypa-yp)/ypa*100))

mapep

model7.9<-trainr(yf,xf,learningrate = 0.2,hidden_dim = 10,numepochs = 200,batch_size = 10,network_type = "rnn")

par(mfrow=c(1,1))

plot(colMeans(model7.9$error),type = 'l',xlab = 'Epoch',ylab = 'Errors')

ytn<-(predictr(model7.9,xt))

yt<-round(ytn*(max-min)+min)

yt

mapet<-mean(abs((yta-yt)/yta*100))

mapet

ypn<-(predictr(model7.9,xp))

yp<-round(ypn*(max-min)+min)

yp

mapep<-mean(abs((ypa-yp)/ypa*100))

mapep

#8

x1<-wxrnn8$x1

x2<-wxrnn8$x2

x3<-wxrnn8$x3

x4<-wxrnn8$x4

x5<-wxrnn8$x5

x6<-wxrnn8$x6

x7<-wxrnn8$x7

x8<-wxrnn8$x8

x9<-wxrnn8$x9

x10<-wxrnn8$x10

x11<-wxrnn8$x11

x12<-wxrnn8$x12

x13<-wxrnn8$x13

x14<-wxrnn8$x14

x15<-wxrnn8$x15

y<-wxrnn8$y

max<-max(x1,x2,x3,x4,x5,x6,x7,x8,x9,x10,x11,x12,x13,x14,x15,y)

min<-min(x1,x2,x3,x4,x5,x6,x7,x8,x9,x10,x11,x12,x13,x14,x15,y)

x1n<-((x1-min)/(max-min))

x2n<-((x2-min)/(max-min))

x3n<-((x3-min)/(max-min))

x4n<-((x4-min)/(max-min))

x5n<-((x5-min)/(max-min))

x6n<-((x6-min)/(max-min))

x7n<-((x7-min)/(max-min))

x8n<-((x8-min)/(max-min))

x9n<-((x9-min)/(max-min))

x10n<-((x10-min)/(max-min))

x11n<-((x11-min)/(max-min))

x12n<-((x12-min)/(max-min))

x13n<-((x13-min)/(max-min))

x14n<-((x14-min)/(max-min))

x15n<-((x15-min)/(max-min))

yn<-((y-min)/(max-min))

xf1n<-x1n[1:132]

xf2n<-x2n[1:132]

xf3n<-x3n[1:132]

xf4n<-x4n[1:132]

xf5n<-x5n[1:132]

xf6n<-x6n[1:132]

xf7n<-x7n[1:132]

xf8n<-x8n[1:132]

xf9n<-x9n[1:132]

xf10n<-x10n[1:132]

xf11n<-x11n[1:132]

xf12n<-x12n[1:132]

xf13n<-x13n[1:132]

xf14n<-x14n[1:132]

xf15n<-x15n[1:132]

yfn<-yn[1:132]

xt1n<-x1n[133:144]

xt2n<-x2n[133:144]

xt3n<-x3n[133:144]

xt4n<-x4n[133:144]

xt5n<-x5n[133:144]

xt6n<-x6n[133:144]

xt7n<-x7n[133:144]

xt8n<-x8n[133:144]

xt9n<-x9n[133:144]

xt10n<-x10n[133:144]

xt11n<-x11n[133:144]

xt12n<-x12n[133:144]

xt13n<-x13n[133:144]

xt14n<-x14n[133:144]

xt15n<-x15n[133:144]

yta<-y[133:144]

xp1n<-x1n[145:156]

xp2n<-x2n[145:156]

xp3n<-x3n[145:156]

xp4n<-x4n[145:156]

xp5n<-x5n[145:156]

xp6n<-x6n[145:156]

xp7n<-x7n[145:156]

xp8n<-x8n[145:156]

xp9n<-x9n[145:156]

xp10n<-x10n[145:156]

xp11n<-x11n[145:156]

xp12n<-x12n[145:156]

xp13n<-x13n[145:156]

xp14n<-x14n[145:156]

xp15n<-x15n[145:156]

ypa<-y[145:156]

xf<-array(c(xf1n,xf2n,xf3n,xf4n,xf5n,xf6n,xf7n,xf8n,xf9n,xf10n,xf11n,xf12n,xf13n,xf14n,xf15n),dim = c(132,1,15))

yf<-array(yfn,dim = c(132,1,1))

xt<-array(c(xt1n,xt2n,xt3n,xt4n,xt5n,xt6n,xt7n,xt8n,xt9n,xt10n,xt11n,xt12n,xt13n,xt14n,xt15n),dim = c(12,1,15))

xp<-array(c(xp1n,xp2n,xp3n,xp4n,xp5n,xp6n,xp7n,xp8n,xp9n,xp10n,xp11n,xp12n,xp13n,xp14n,xp15n),dim = c(12,1,15))

model8.1<-trainr(yf,xf,learningrate = 0.05,hidden_dim = 3,numepochs = 1500,batch_size = 10,network_type = "rnn")

par(mfrow=c(1,1))

plot(colMeans(model8.1$error),type = 'l',xlab = 'Epoch',ylab = 'Errors')

ytn<-(predictr(model8.1,xt))

yt<-round(ytn*(max-min)+min)

yt

mapet<-mean(abs((yta-yt)/yta*100))

mapet

ypn<-(predictr(model8.1,xp))

yp<-round(ypn*(max-min)+min)

yp

mapep<-mean(abs((ypa-yp)/ypa*100))

mapep

model8.2<-trainr(yf,xf,learningrate = 0.05,hidden_dim = 5,numepochs = 1400,batch_size = 10,network_type = "rnn")

par(mfrow=c(1,1))

plot(colMeans(model8.2$error),type = 'l',xlab = 'Epoch',ylab = 'Errors')

ytn<-(predictr(model8.2,xt))

yt<-round(ytn*(max-min)+min)

yt

mapet<-mean(abs((yta-yt)/yta*100))

mapet

ypn<-(predictr(model8.2,xp))

yp<-round(ypn*(max-min)+min)

yp

mapep<-mean(abs((ypa-yp)/ypa*100))

mapep

model8.3<-trainr(yf,xf,learningrate = 0.05,hidden_dim = 10,numepochs = 1000,batch_size = 10,network_type = "rnn")

par(mfrow=c(1,1))

plot(colMeans(model8.3$error),type = 'l',xlab = 'Epoch',ylab = 'Errors')

ytn<-(predictr(model8.3,xt))

yt<-round(ytn*(max-min)+min)

yt

mapet<-mean(abs((yta-yt)/yta*100))

mapet

ypn<-(predictr(model8.3,xp))

yp<-round(ypn*(max-min)+min)

yp

mapep<-mean(abs((ypa-yp)/ypa*100))

mapep

model8.4<-trainr(yf,xf,learningrate = 0.1,hidden_dim = 3,numepochs = 800,batch_size = 10,network_type = "rnn")

par(mfrow=c(1,1))

plot(colMeans(model8.4$error),type = 'l',xlab = 'Epoch',ylab = 'Errors')

ytn<-(predictr(model8.4,xt))

yt<-round(ytn*(max-min)+min)

yt

mapet<-mean(abs((yta-yt)/yta*100))

mapet

ypn<-(predictr(model8.4,xp))

yp<-round(ypn*(max-min)+min)

yp

mapep<-mean(abs((ypa-yp)/ypa*100))

mapep

model8.5<-trainr(yf,xf,learningrate = 0.1,hidden_dim = 5,numepochs = 600,batch_size = 10,network_type = "rnn")

par(mfrow=c(1,1))

plot(colMeans(model8.5$error),type = 'l',xlab = 'Epoch',ylab = 'Errors')

ytn<-(predictr(model8.5,xt))

yt<-round(ytn*(max-min)+min)

yt

mapet<-mean(abs((yta-yt)/yta*100))

mapet

ypn<-(predictr(model8.5,xp))

yp<-round(ypn*(max-min)+min)

yp

mapep<-mean(abs((ypa-yp)/ypa*100))

mapep

model8.6<-trainr(yf,xf,learningrate = 0.1,hidden_dim = 10,numepochs = 400,batch_size = 10,network_type = "rnn")

par(mfrow=c(1,1))

plot(colMeans(model8.6$error),type = 'l',xlab = 'Epoch',ylab = 'Errors')

ytn<-(predictr(model8.6,xt))

yt<-round(ytn*(max-min)+min)

yt

mapet<-mean(abs((yta-yt)/yta*100))

mapet

ypn<-(predictr(model8.6,xp))

yp<-round(ypn*(max-min)+min)

yp

mapep<-mean(abs((ypa-yp)/ypa*100))

mapep

model8.7<-trainr(yf,xf,learningrate = 0.2,hidden_dim = 3,numepochs = 300,batch_size = 10,network_type = "rnn")

par(mfrow=c(1,1))

plot(colMeans(model8.7$error),type = 'l',xlab = 'Epoch',ylab = 'Errors')

ytn<-(predictr(model8.7,xt))

yt<-round(ytn*(max-min)+min)

yt

mapet<-mean(abs((yta-yt)/yta*100))

mapet

ypn<-(predictr(model8.7,xp))

yp<-round(ypn*(max-min)+min)

yp

mapep<-mean(abs((ypa-yp)/ypa*100))

mapep

model8.8<-trainr(yf,xf,learningrate = 0.2,hidden_dim = 5,numepochs = 250,batch_size = 10,network_type = "rnn")

par(mfrow=c(1,1))

plot(colMeans(model8.8$error),type = 'l',xlab = 'Epoch',ylab = 'Errors')

ytn<-(predictr(model8.8,xt))

yt<-round(ytn*(max-min)+min)

yt

mapet<-mean(abs((yta-yt)/yta*100))

mapet

ypn<-(predictr(model8.8,xp))

yp<-round(ypn*(max-min)+min)

yp

mapep<-mean(abs((ypa-yp)/ypa*100))

mapep

model8.9<-trainr(yf,xf,learningrate = 0.2,hidden_dim = 10,numepochs = 200,batch_size = 10,network_type = "rnn")

par(mfrow=c(1,1))

plot(colMeans(model8.9$error),type = 'l',xlab = 'Epoch',ylab = 'Errors')

ytn<-(predictr(model8.9,xt))

yt<-round(ytn*(max-min)+min)

yt

mapet<-mean(abs((yta-yt)/yta*100))

mapet

ypn<-(predictr(model8.9,xp))

yp<-round(ypn*(max-min)+min)

yp

mapep<-mean(abs((ypa-yp)/ypa*100))

mapep

#9

x1<-wxrnn9$x1

x2<-wxrnn9$x2

x3<-wxrnn9$x3

x4<-wxrnn9$x4

x5<-wxrnn9$x5

x6<-wxrnn9$x6

x7<-wxrnn9$x7

x8<-wxrnn9$x8

x9<-wxrnn9$x9

x10<-wxrnn9$x10

x11<-wxrnn9$x11

x12<-wxrnn9$x12

x13<-wxrnn9$x13

x14<-(wxrnn9$x14)/10

x15<-wxrnn9$x15

x16<-wxrnn9$x16

x17<-wxrnn9$x17

x18<-wxrnn9$x18

x19<-wxrnn9$x19

x20<-wxrnn9$x20

x21<-wxrnn9$x21

y<-wxrnn9$y

max<-max(x1,x2,x3,x4,x5,x6,x7,x8,x9,x10,x11,x12,x13,x14,x15,x16,x17,x18,x19,x20,x21,y)

min<-min(x1,x2,x3,x4,x5,x6,x7,x8,x9,x10,x11,x12,x13,x14,x15,x16,x17,x18,x19,x20,x21,y)

x1n<-((x1-min)/(max-min))

x2n<-((x2-min)/(max-min))

x3n<-((x3-min)/(max-min))

x4n<-((x4-min)/(max-min))

x5n<-((x5-min)/(max-min))

x6n<-((x6-min)/(max-min))

x7n<-((x7-min)/(max-min))

x8n<-((x8-min)/(max-min))

x9n<-((x9-min)/(max-min))

x10n<-((x10-min)/(max-min))

x11n<-((x11-min)/(max-min))

x12n<-((x12-min)/(max-min))

x13n<-((x13-min)/(max-min))

x14n<-((x14-min)/(max-min))

x15n<-((x15-min)/(max-min))

x16n<-((x16-min)/(max-min))

x17n<-((x17-min)/(max-min))

x18n<-((x18-min)/(max-min))

x19n<-((x19-min)/(max-min))

x20n<-((x20-min)/(max-min))

x21n<-((x21-min)/(max-min))

yn<-((y-min)/(max-min))

xf1n<-x1n[1:132]

xf2n<-x2n[1:132]

xf3n<-x3n[1:132]

xf4n<-x4n[1:132]

xf5n<-x5n[1:132]

xf6n<-x6n[1:132]

xf7n<-x7n[1:132]

xf8n<-x8n[1:132]

xf9n<-x9n[1:132]

xf10n<-x10n[1:132]

xf11n<-x11n[1:132]

xf12n<-x12n[1:132]

xf13n<-x13n[1:132]

xf14n<-x14n[1:132]

xf15n<-x15n[1:132]

xf16n<-x16n[1:132]

xf17n<-x17n[1:132]

xf18n<-x18n[1:132]

xf19n<-x19n[1:132]

xf20n<-x20n[1:132]

xf21n<-x21n[1:132]

yfn<-yn[1:132]

xt1n<-x1n[133:144]

xt2n<-x2n[133:144]

xt3n<-x3n[133:144]

xt4n<-x4n[133:144]

xt5n<-x5n[133:144]

xt6n<-x6n[133:144]

xt7n<-x7n[133:144]

xt8n<-x8n[133:144]

xt9n<-x9n[133:144]

xt10n<-x10n[133:144]

xt11n<-x11n[133:144]

xt12n<-x12n[133:144]

xt13n<-x13n[133:144]

xt14n<-x14n[133:144]

xt15n<-x15n[133:144]

xt16n<-x16n[133:144]

xt17n<-x17n[133:144]

xt18n<-x18n[133:144]

xt19n<-x19n[133:144]

xt20n<-x20n[133:144]

xt21n<-x21n[133:144]

yta<-y[133:144]

xp1n<-x1n[145:156]

xp2n<-x2n[145:156]

xp3n<-x3n[145:156]

xp4n<-x4n[145:156]

xp5n<-x5n[145:156]

xp6n<-x6n[145:156]

xp7n<-x7n[145:156]

xp8n<-x8n[145:156]

xp9n<-x9n[145:156]

xp10n<-x10n[145:156]

xp11n<-x11n[145:156]

xp12n<-x12n[145:156]

xp13n<-x13n[145:156]

xp14n<-x14n[145:156]

xp15n<-x15n[145:156]

xp16n<-x16n[145:156]

xp17n<-x17n[145:156]

xp18n<-x18n[145:156]

xp19n<-x19n[145:156]

xp20n<-x20n[145:156]

xp21n<-x21n[145:156]

ypa<-y[145:156]

xf<-array(c(xf1n,xf2n,xf3n,xf4n,xf5n,xf6n,xf7n,xf8n,xf9n,xf10n,xf11n,xf12n,

xf13n,xf14n,xf15n,xf16n,xf17n,xf18n,xf19n,xf20n,xf21n),dim = c(132,1,21))

yf<-array(yfn,dim = c(132,1,1))

xt<-array(c(xt1n,xt2n,xt3n,xt4n,xt5n,xt6n,xt7n,xt8n,xt9n,xt10n,xt11n,xt12n,

xt13n,xt14n,xt15n,xt16n,xt17n,xt18n,xt19n,xt20n,xt21n),dim = c(12,1,21))

xp<-array(c(xp1n,xp2n,xp3n,xp4n,xp5n,xp6n,xp7n,xp8n,xp9n,xp10n,xp11n,xp12n,

xp13n,xp14n,xp15n,xp16n,xp17n,xp18n,xp19n,xp20n,xp21n),dim = c(12,1,21))

model9.1<-trainr(yf,xf,learningrate = 0.05,hidden_dim = 3,numepochs = 1500,batch_size = 10,network_type = "rnn")

par(mfrow=c(1,1))

plot(colMeans(model9.1$error),type = 'l',xlab = 'Epoch',ylab = 'Errors')

ytn<-(predictr(model9.1,xt))

yt<-round(ytn*(max-min)+min)

yt

mapet<-mean(abs((yta-yt)/yta*100))

mapet

ypn<-(predictr(model9.1,xp))

yp<-round(ypn*(max-min)+min)

yp

mapep<-mean(abs((ypa-yp)/ypa*100))

mapep

model9.2<-trainr(yf,xf,learningrate = 0.05,hidden_dim = 5,numepochs = 1400,batch_size = 10,network_type = "rnn")

par(mfrow=c(1,1))

plot(colMeans(model9.2$error),type = 'l',xlab = 'Epoch',ylab = 'Errors')

ytn<-(predictr(model9.2,xt))

yt<-round(ytn*(max-min)+min)

yt

mapet<-mean(abs((yta-yt)/yta*100))

mapet

ypn<-(predictr(model9.2,xp))

yp<-round(ypn*(max-min)+min)

yp

mapep<-mean(abs((ypa-yp)/ypa*100))

mapep

model9.3<-trainr(yf,xf,learningrate = 0.05,hidden_dim = 10,numepochs = 1200,batch_size = 10,network_type = "rnn")

par(mfrow=c(1,1))

plot(colMeans(model9.3$error),type = 'l',xlab = 'Epoch',ylab = 'Errors')

ytn<-(predictr(model9.3,xt))

yt<-round(ytn*(max-min)+min)

yt

mapet<-mean(abs((yta-yt)/yta*100))

mapet

ypn<-(predictr(model9.3,xp))

yp<-round(ypn*(max-min)+min)

yp

mapep<-mean(abs((ypa-yp)/ypa*100))

mapep

model9.4<-trainr(yf,xf,learningrate = 0.1,hidden_dim = 3,numepochs = 1000,batch_size = 10,network_type = "rnn")

par(mfrow=c(1,1))

plot(colMeans(model9.4$error),type = 'l',xlab = 'Epoch',ylab = 'Errors')

ytn<-(predictr(model9.4,xt))

yt<-round(ytn*(max-min)+min)

yt

mapet<-mean(abs((yta-yt)/yta*100))

mapet

ypn<-(predictr(model9.4,xp))

yp<-round(ypn*(max-min)+min)

yp

mapep<-mean(abs((ypa-yp)/ypa*100))

mapep

model9.5<-trainr(yf,xf,learningrate = 0.1,hidden_dim = 5,numepochs = 800,batch_size = 10,network_type = "rnn")

par(mfrow=c(1,1))

plot(colMeans(model9.5$error),type = 'l',xlab = 'Epoch',ylab = 'Errors')

ytn<-(predictr(model9.5,xt))

yt<-round(ytn*(max-min)+min)

yt

mapet<-mean(abs((yta-yt)/yta*100))

mapet

ypn<-(predictr(model9.5,xp))

yp<-round(ypn*(max-min)+min)

yp

mapep<-mean(abs((ypa-yp)/ypa*100))

mapep

model9.6<-trainr(yf,xf,learningrate = 0.1,hidden_dim = 10,numepochs = 500,batch_size = 10,network_type = "rnn")

par(mfrow=c(1,1))

plot(colMeans(model9.6$error),type = 'l',xlab = 'Epoch',ylab = 'Errors')

ytn<-(predictr(model9.6,xt))

yt<-round(ytn*(max-min)+min)

yt

mapet<-mean(abs((yta-yt)/yta*100))

mapet

ypn<-(predictr(model9.6,xp))

yp<-round(ypn*(max-min)+min)

yp

mapep<-mean(abs((ypa-yp)/ypa*100))

mapep

model9.7<-trainr(yf,xf,learningrate = 0.2,hidden_dim = 3,numepochs = 400,batch_size = 10,network_type = "rnn")

par(mfrow=c(1,1))

plot(colMeans(model9.7$error),type = 'l',xlab = 'Epoch',ylab = 'Errors')

ytn<-(predictr(model9.7,xt))

yt<-round(ytn*(max-min)+min)

yt

mapet<-mean(abs((yta-yt)/yta*100))

mapet

ypn<-(predictr(model9.7,xp))

yp<-round(ypn*(max-min)+min)

yp

mapep<-mean(abs((ypa-yp)/ypa*100))

mapep

model9.8<-trainr(yf,xf,learningrate = 0.2,hidden_dim = 5,numepochs = 300,batch_size = 10,network_type = "rnn")

par(mfrow=c(1,1))

plot(colMeans(model9.8$error),type = 'l',xlab = 'Epoch',ylab = 'Errors')

ytn<-(predictr(model9.8,xt))

yt<-round(ytn*(max-min)+min)

yt

mapet<-mean(abs((yta-yt)/yta*100))

mapet

ypn<-(predictr(model9.8,xp))

yp<-round(ypn*(max-min)+min)

yp

mapep<-mean(abs((ypa-yp)/ypa*100))

mapep

model9.9<-trainr(yf,xf,learningrate = 0.2,hidden_dim = 10,numepochs = 200,batch_size = 10,network_type = "rnn")

par(mfrow=c(1,1))

plot(colMeans(model9.9$error),type = 'l',xlab = 'Epoch',ylab = 'Errors')

ytn<-(predictr(model9.9,xt))

yt<-round(ytn*(max-min)+min)

yt

mapet<-mean(abs((yta-yt)/yta*100))

mapet

ypn<-(predictr(model9.9,xp))

yp<-round(ypn*(max-min)+min)

yp

mapep<-mean(abs((ypa-yp)/ypa*100))

mapep
